# Variability and implications of recurrent implantation failure definitions used in the scientific literature: a systematic review

**DOI:** 10.1093/hropen/hoaf033

**Published:** 2025-06-18

**Authors:** Jessica K Lu, Yin Jun Law, Ning Zhang, Evangelia T Katsika, Efstratios M Kolibianakis, Christos A Venetis

**Affiliations:** School of Women’s and Children’s Health, UNSW Medicine and Health, University of New South Wales, Sydney, Randwick, Australia; School of Women’s and Children’s Health, UNSW Medicine and Health, University of New South Wales, Sydney, Randwick, Australia; School of Women’s and Children’s Health, UNSW Medicine and Health, University of New South Wales, Sydney, Randwick, Australia; Unit for Human Reproduction, 1st Department of Obstetrics and Gynaecology, Medical School, Faculty of Health Sciences, Aristotle University of Thessaloniki, Papageorgiou General Hospital, Thessaloniki, Greece; Unit for Human Reproduction, 1st Department of Obstetrics and Gynaecology, Medical School, Faculty of Health Sciences, Aristotle University of Thessaloniki, Papageorgiou General Hospital, Thessaloniki, Greece; School of Women’s and Children’s Health, UNSW Medicine and Health, University of New South Wales, Sydney, Randwick, Australia; Unit for Human Reproduction, 1st Department of Obstetrics and Gynaecology, Medical School, Faculty of Health Sciences, Aristotle University of Thessaloniki, Papageorgiou General Hospital, Thessaloniki, Greece

**Keywords:** recurrent implantation failure, repeated implantation failure, definition, assisted reproductive technologies, ART, IVF

## Abstract

**STUDY QUESTION:**

How is recurrent implantation failure (RIF) defined in published literature and what is the prognostic agreement of these definitions with recently introduced RIF criteria by ESHRE?

**SUMMARY ANSWER:**

RIF definitions used in current clinical studies are highly variable and only a low proportion of published studies on RIF meet the ESHRE RIF diagnostic threshold.

**WHAT IS KNOWN ALREADY:**

RIF is a key cause of ART failure and growing focus of ART research. However, RIF remains poorly and inconsistently defined in published literature, thereby making the interpretation and clinical applicability of RIF research difficult and highly problematic.

**STUDY DESIGN, SIZE, DURATION:**

The electronic databases EMBASE (Ovid), PubMed, Cochrane Central Register Of Controlled Trials (CENTRAL), Scopus, and Web of Science were systematically searched up to 30 June 2024 using the search terms ‘recurrent implantation failure’ and ‘repeated implantation failure’ for original peer-reviewed journal articles that included RIF patients.

**PARTICIPANTS/MATERIALS, SETTING, METHODS:**

The following data were manually extracted from eligible full-text articles: study methodology and characteristics, ART characteristics, and the RIF definition used. Extracted RIF definitions were analysed according to predetermined specifiers. The prognostic profile of these RIF definitions was compared with the 2023 ESHRE-recommended threshold for RIF diagnosis.

**MAIN RESULTS AND THE ROLE OF CHANCE:**

The literature search identified 9853 studies, of which 748 were eligible for inclusion. Of these 748 studies, 589 studies (78.7%) provided one RIF definition, 83 studies (11.1%) used two definitions, three studies (0.4%) provided three or more definitions while 73 studies (9.8%) did not provide a definition for RIF. Of the 838 RIF definitions retrieved, there were a total of 503 unique RIF definitions. The three most common specifiers used to define RIF were embryo morphological quality (n = 491, 58.6% of RIF definitions), number of transfer events (n = 439, 52.4%), and cumulative number of embryos transferred (n = 326, 38.9%). RIF was most frequently diagnosed as ‘failure of ≥3 embryo transfer events’ (n = 26) and ‘failure of ≥3 stimulated cycles’ (n = 22). The threshold for defining RIF based on the cumulative number of embryos transferred in total was significantly higher for cleavage-stage embryos compared to blastocysts (incidence rate ratio 2.15, *P* < 0.001). In most cases, the RIF definitions used did not meet the ESHRE-recommended RIF diagnostic threshold of >60% cumulative predicted chance of implantation.

**LIMITATIONS, REASONS FOR CAUTION:**

This systematic review excluded abstracts and case-series. Several studies provided RIF definitions with limited detail or ambiguous terminology with potential for misclassification or misinterpretation.

**WIDER IMPLICATIONS OF THE FINDINGS:**

There remains a high degree of variability and discrepancy between RIF definitions used in current clinical studies on RIF. Furthermore, the low proportion of studies meeting the ESHRE RIF diagnostic threshold casts doubts on whether the populations in these studies were truly RIF patients. As such, published research findings should be interpreted with caution. To enable wider clinical applicability of future research on the aetiology of and therapeutic interventions for RIF, it is imperative that a standardized RIF definition is meticulously implemented.

**STUDY FUNDING/COMPETING INTEREST(S):**

No specific external funding was sought or obtained for this study. All authors report no conflicts of interest with regard to this study.

**TRIAL REGISTRATION NUMBER:**

This trial was registered in PROSPERO (CRD42022295349).

WHAT DOES THIS MEAN FOR PATIENTS? In assisted reproduction, the term ‘recurrent implantation failure (RIF)’ is used to describe the lack of pregnancy despite multiple IVF cycles and embryo transfer attempts. However, previous research has shown that the definition of RIF is highly variable in published studies on this topic.

This study has evaluated the extent of this variation and analysed how available scientific research defines RIF. Results demonstrate that the majority of published studies use their own unique definition for RIF and no widely accepted RIF definition currently exists. Furthermore, only a small proportion of published RIF definitions meet the recently introduced criteria recommended by the internationally recognized European Society for Human Reproduction and Embryology (ESHRE). This is problematic as it means that available RIF research cannot be widely applied to all RIF patients, who may be at risk of undergoing tests or treatments for RIF that are unnecessary or unhelpful. To enable more relevant and robust clinical research on RIF, a standardized RIF definition needs to be implemented.

## Introduction

Embryo implantation is a complex process that requires synchrony between a competent blastocyst and a receptive endometrium via the successful interplay of embryonic and maternal factors. Recurrent or repeated implantation failure (RIF) is a poorly defined clinical entity encompassing the lack of successful embryo implantation following multiple embryo transfers in ART cycles. Despite advancements in ART, RIF still poses a major clinical challenge as a key cause of recurrent IVF and ICSI treatment failure. RIF has been reported to affect up to 15% of women undergoing IVF treatment (Margalioth *et al.*, 2006; Busnelli *et al.*, 2020), including women who respond appropriately to controlled ovarian stimulation and produce morphologically good-quality embryos.

Not surprisingly, RIF has been the focus of an increasing body of research over the last two decades (Wang *et al.*, 2022), including studies examining its aetiology and relevant therapeutic interventions. However, the high inconsistency regarding its definition renders the interpretation of published evidence highly problematic.

This inconsistency was firstly described a decade ago by Polanski *et al.* (2014) in a systematic review assessing the use of RIF definitions in scientific literature, which found that the most commonly described definitions were ‘three or more failed treatment cycles’ or ‘two or more failed cycles’ (Polanski *et al.*, 2014). Since 2014, however, the number of studies in the field of RIF has increased exponentially and newer definitions of RIF have been proposed, taking into account modern therapeutic strategies, such as preimplantation genetic testing (PGT) for aneuploidy (Pagidas *et al.*, 2008; Pirtea *et al.*, 2021) and elective preservation of all embryos (Magdi *et al.*, 2016, 2017; Rigos *et al.*, 2021). Additionally, researchers have proposed individual algorithms for diagnosing a patient with RIF, on the basis of important prognostic factors such as age, number, and quality of embryos (Somigliana *et al.*, 2018; Ata *et al.*, 2021; Rozen *et al.*, 2021). Recently, ESHRE has introduced criteria which aim to standardize the diagnosis of RIF.

The present systematic review aims to provide an updated overview and analysis of the definitions of RIF used in published literature and to assess their prognostic agreement with the recently introduced RIF criteria by ESHRE.

## Methods

**Figure 1.**
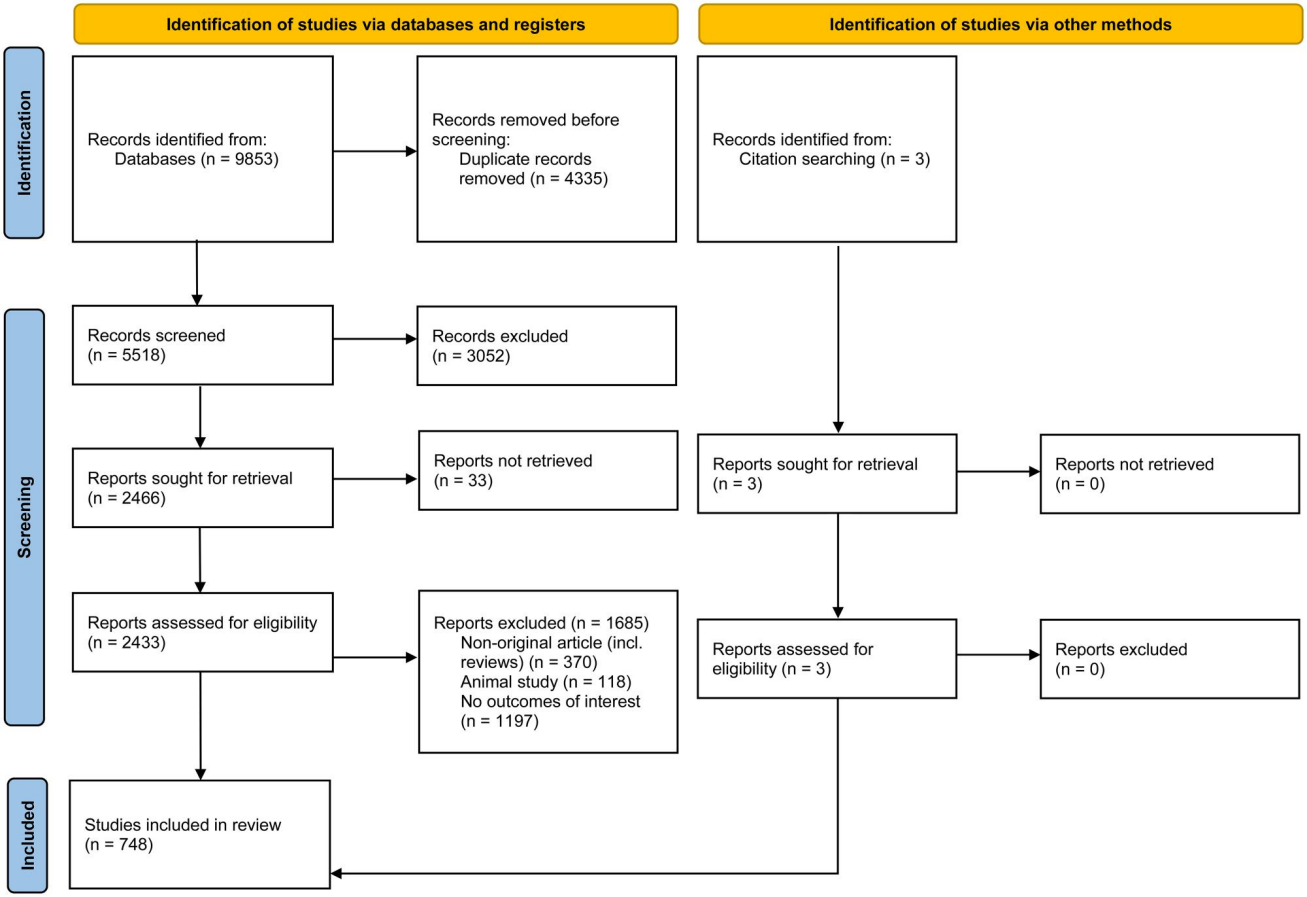
Preferred Reporting Items for Systematic Reviews and Meta-analyses (PRISMA) flow diagram.

This systematic review was registered in PROSPERO (CRD42022295349) and conducted in accordance with the PRISMA guidelines (Page *et al.*, 2021) (Fig. 1).

### Eligibility criteria

All original research articles published in peer-reviewed journals of any study design or language reporting to have included patients with RIF (either as the primary study group or subgroup) were included. Case reports, conference abstracts, reviews, and textbook chapters were excluded.

### Sources

Studies were identified by systematically searching the databases EMBASE (Ovid), PubMed, Cochrane Central Register of Controlled Trials (CENTRAL), Scopus, and Web of Science from their inception until 30 June 2024.

### Search strategy

The literature search was performed by three independent reviewers (J.K.L., Y.J.L., and N.Z.) using a predetermined search strategy with the following search terms: ‘recurrent implantation failure’, ‘repeated implantation failure’.

### Study selection

Search results were collated in reference management software (Endnote version 20; Clarivate Analytics; Philadelphia, PA, USA) and duplicates were removed prior to screening. Compiled studies were sequentially screened by three independent reviewers (J.K.L., Y.J.L., and N.Z.), first by scanning article titles for relevance to reproductive medicine and assisted reproductive technology, followed by scrutinizing abstracts and then the full-text article to select studies that met eligibility criteria for inclusion in this systematic review. Reference lists of relevant publications were manually searched for additional eligible references. Any disagreement between reviewers about study selection was resolved by a fourth reviewer (C.A.V.).

### Data extraction

Four independent reviewers (J.K.L., Y.J.L, N.Z., and E.T.K.) extracted the following predefined data from eligible full-text articles: methodology (journal, study type and design, study period, setting, country and region of origin, main research question), study characteristics (inclusion and exclusion criteria, maternal age limit, sample size, whether the study subjects comprised of RIF patients entirely or as a subgroup only, female age, cause of infertility if known, RIF incidence if reported), ART characteristics (fertilization method, embryo type, i.e. fresh and/or frozen, number of embryos transferred per patient, use of PGT, embryo developmental stage, and quality), and the RIF definition used. Studies were grouped by type (interventional, predictive, prognostic, diagnostic) according to the clinical question addressed: interventional studies analysed the effectiveness of treatments for RIF, predictive studies analysed aetiological and/or risk factors associated with RIF, prognostic studies analysed outcomes in women with RIF and diagnostic studies analysed modalities for diagnosing RIF. Only RIF definitions that were provided in the ‘Materials and methods’ section of eligible studies and/or were explicitly stated as being used for patient recruitment were accepted.

### Statistical analysis

To facilitate interpretation of and comparison between the RIF definitions extracted, worded definitions were deconstructed into their core components according to the following specifiers: cumulative number of embryos transferred, number of embryo transfer events, number of embryos transferred per cycle, embryo developmental stage at transfer and morphological quality, embryo type (fresh and/or frozen), whether genetic testing for aneuploidy was performed, number of stimulated cycles, whether failed cycles were consecutive, maternal age, maternal ovarian reserve, how failed implantation was defined, and any additional specifiers not mentioned above.

A set of predetermined rules was devised to ensure this process of interpreting and transcribing worded RIF definitions using these specifiers was consistent and reproducible (Supplementary File S1).

Statistical analysis was performed to determine the use and variability in RIF definitions described in the literature, including the proportion of studies using each of the core components listed above. Recorded combinations of RIF definition components were analysed to determine the most common RIF definitions applied in published literature.

To examine whether differing embryo developmental stage and maternal age were reflected in published RIF definitions, subgroup analyses were undertaken to compare RIF definitions using cleavage-stage versus blastocyst-stage embryos and for maternal age <40 versus ≥40 years.

Binomial logistic regression was used to assess for association between year of publication and embryo developmental stage for defining RIF. Multinomial logistic regression was conducted to quantify the effect of publication year on the embryo type (fresh and/or frozen) and developmental stage (cleavage or blastocyst) used and the region of study origin. Poisson regression was used to examine the effect of publication year on the number of specifiers used to define RIF, as well as the effect of embryo developmental stage on the minimum number of: (a) cumulative embryos transferred, (b) embryo transfer events, and (c) stimulated cycles required to meet the definition of RIF. The results were evaluated using odds ratios (ORs), relative risk ratios (RRRs), and incidence rate ratios (IRRs), respectively, and statistical significance was defined as *P* < 0.05.

To assess the prognostic profile and comparability of RIF definitions in the literature, we applied the thresholds for maternal age, total number of embryo transfer events, and embryo ploidy status used by published RIF definitions to aggregated ART data generated from European registries recently published by ESHRE (ESHRE Working Group on Recurrent Implantation Failure *et al.*, 2023) to estimate the cumulative likelihood of pregnancy for each reported definition using the following four models:

1. ‘Worst case scenario’ model—Maternal age was taken as the maximum reported age of included participants. For studies that did not specify cut-off values for maternal age or transfer events, maternal age was assumed to be ≥40 years and embryo transfer events ≥2 as the minimum thresholds for this analysis.
2. ‘Optimistic theoretical cohort’ model—Maternal age was assumed to be <35 years. The number of embryo transfer events was assumed to be ≥2 if not otherwise specified.
3. ‘Available data’ model—Only studies reporting both maximum maternal age and minimum number of embryo transfers were included.
4. ‘Optimistic theoretical minimum embryo transfer’ model—Maternal age was assumed to be <35 years for all studies reporting a minimum number of embryo transfers.

It should be noted that this additional analysis was a *post hoc* decision as the ESHRE criteria on RIF had been published in 2023 (ESHRE Working Group on Recurrent Implantation Failure *et al.*, 2023) (after protocol registration) and it was considered highly relevant to assess the concordance of the scientific literature with the newly introduced criteria.

## Results

### Characteristics of included studies

**Figure 2.**
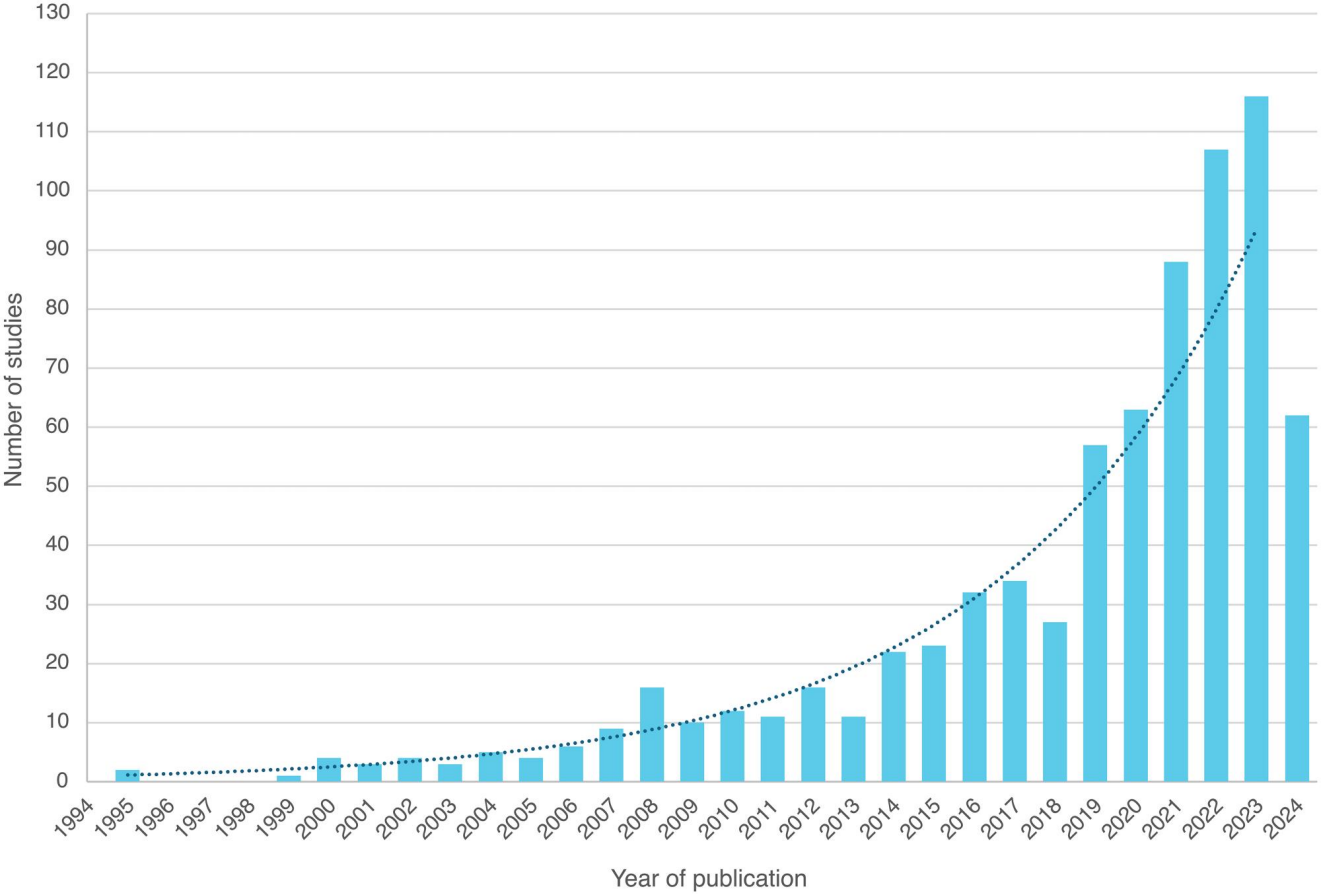
Number of articles published per year included in this systematic review. Note: In 2024, articles with publication date up to 30 June were included in this systematic review.

The systematic search resulted in 9853 studies and the full text of 2433 articles were assessed for eligibility. A total of 748 studies met the inclusion criteria and were included in the review (Supplementary File S2). The characteristics of these studies are summarized in Table 1. The number of included studies by publication year increased exponentially over time (averaging 17.0% per year) (Fig. 2), particularly in Asia, which has produced 71.9% of included studies published after 2014, and over half (50.2%) of the included studies published after 2022 have originated from China (Supplementary Fig. S1).

**Table 1.** Characteristics of included studies.

Study characteristics	Number of studies (n)	%
Total included studies	748	100
***Country***		
China	236	31.5
Iran	88	11.8
Turkey	52	6.9
Spain	29	3.9
Other (51 other countries)	343	46.0
***Region of origin***		
Africa	13	1.7
Asia	497	66.5
Europe	182	24.3
North America	32	4.3
Oceania	10	1.3
South America	12	1.7
Not specified	3	0.4
***Study design***		
Case-control	224	30.0
Cohort	287	38.4
Cross-sectional	14	1.9
Interventional (non-RCT)	32	4.3
Observational	82	11.0
Pilot	16	2.1
RCT	73	9.8
Other	20	2.7
***Study approach***		
Prospective	298	39.8
Retrospective	435	58.2
N/A (cross-sectional)	15	2.0
***Article type***		
Diagnostic	98	13.1
Interventional	287	38.4
Predictive	311	41.6
Prognostic	52	6.9
***Setting***		
Hospital	370	49.5
Fertility centre/clinic	267	35.7
Research institute	28	3.7
University	16	2.1
Database	12	1.6
NR	55	7.4
***Total sample size (n = 739, excluding nine studies that did not report)***	**Participants (n)**	
Minimum	8	
Maximum	45 921	
Mean	356.5	
Standard deviation	1896.7	
***RIF sample size (n = 716, excluding 32 studies that did not report)***	**Participants (n)**	
Minimum	3	
Maximum	45 921	
Mean	242.3	
Standard deviation	1822.4	

NR, not reported; RCT, randomized controlled trial; RIF, recurrent implantation failure.

**Figure 3.**
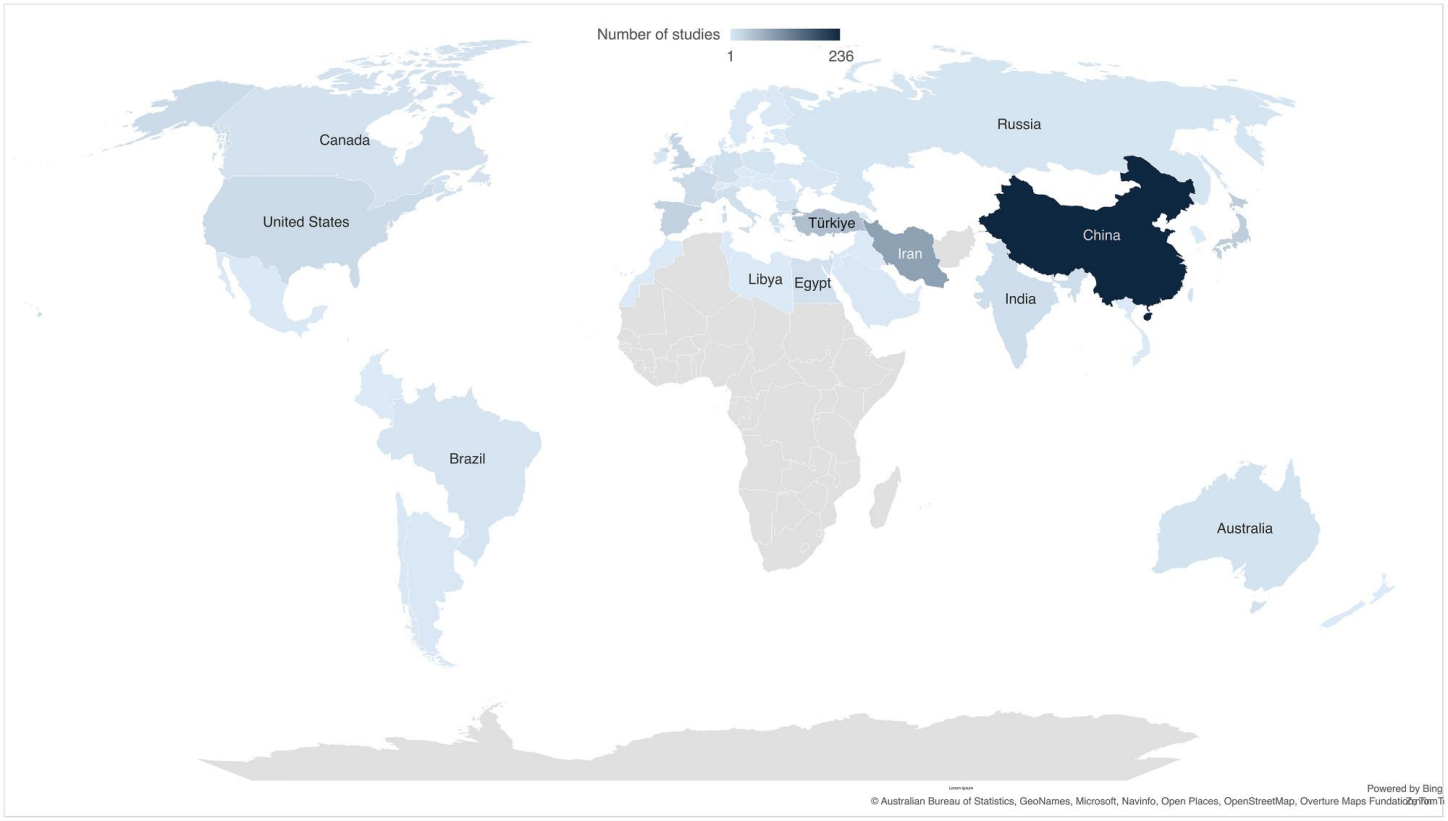
Distribution of country of origin for articles included in the review.

The studies were performed in 57 countries, most frequently originating from China (n = 236, 31.5%), Iran (n = 88, 11.8%), and Turkey (n = 52, 7.0%), as illustrated in Fig. 3. Of note, the most common study designs were cohort (n = 287, 38.4%) and case-control (n = 224, 30.0%), while randomized control trials (RCT) only comprised of 9.8% (n = 73) of the included studies.

### RIF definitions

Of the 748 included studies, 589 studies (78.7%) used a single definition, 83 studies (11.1%) used two definitions, three studies (0.4%) provided three or more definitions, while 73 studies (9.8%) did not provide a definition for RIF.

### Justification of RIF definition

Of the 675 studies that reported at least one definition (765 definitions provided in total), 520 studies (68.0%) did not justify their definition/s of RIF using published data while 245 studies (32.0%) based their definition/s on published literature.

### RIF definitions: analysis of criteria used

**Figure 4.**
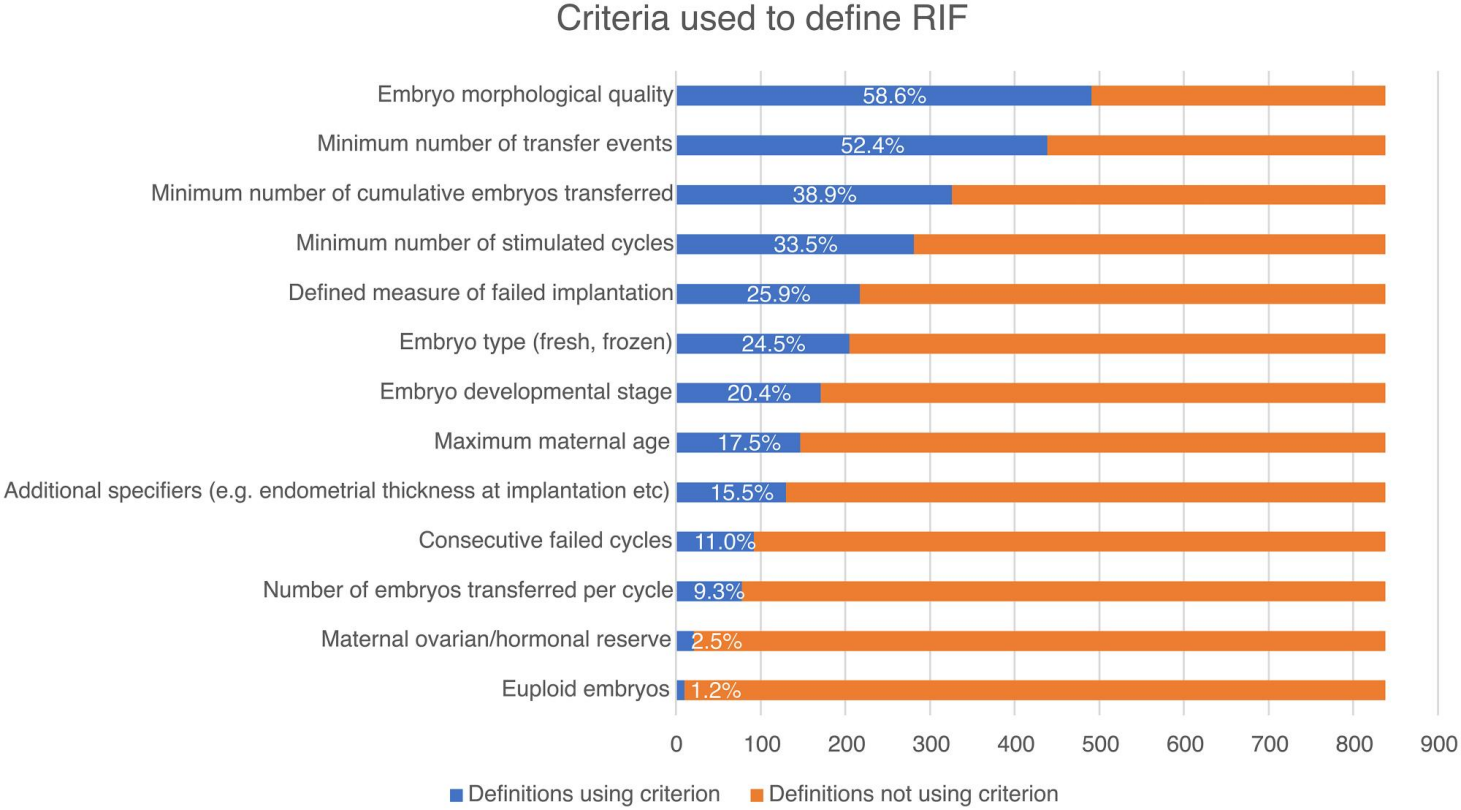
Specifiers used to define recurrent implantation failure.

**Figure 5.**
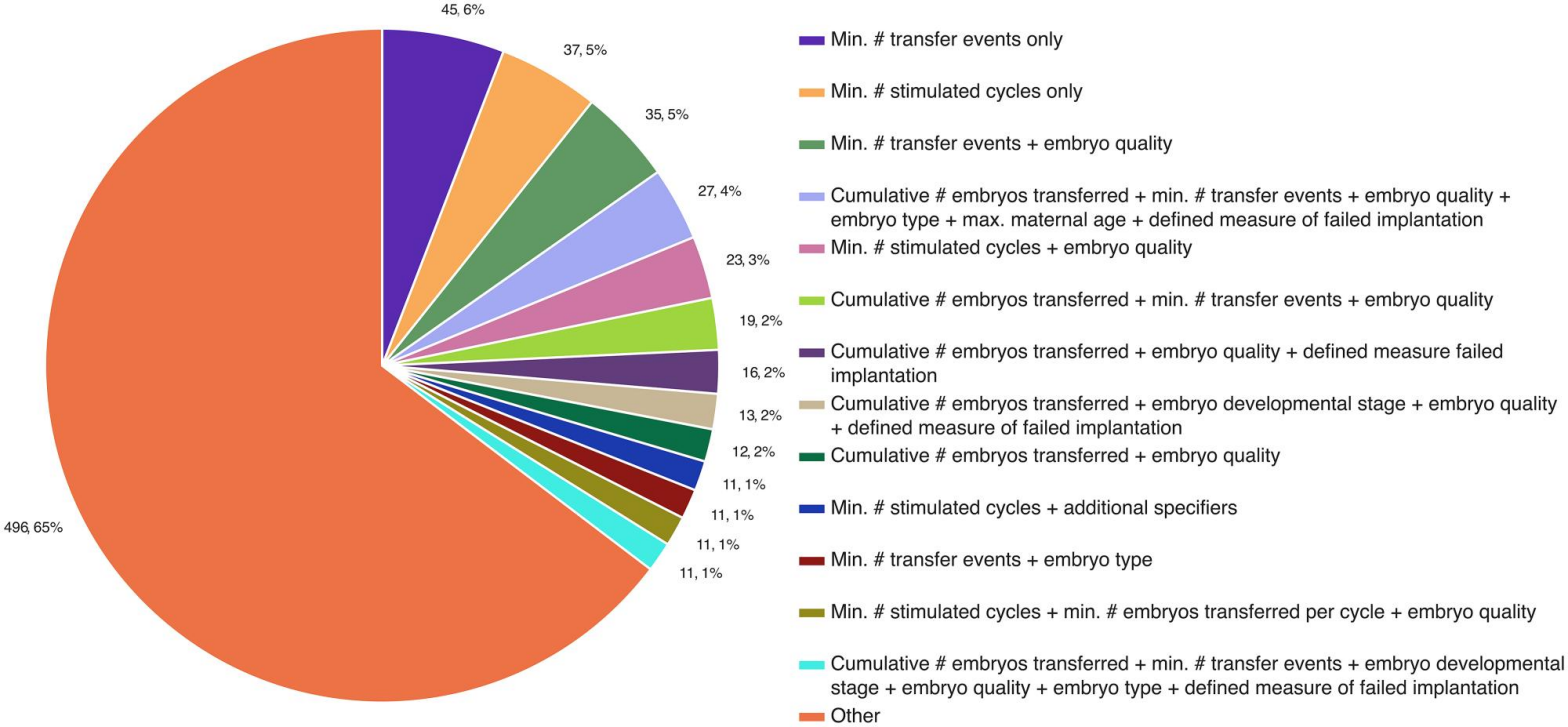
Common combinations of specifiers used to define RIF (n, %). Min., minimum; max., maximum; #, number of; RIF, recurrent implantation failure.

Details of the 838 extracted RIF definitions are summarized in Table 2. The three most common specifiers used to define RIF were embryo morphological quality (n = 491, 58.6%), followed by number of transfer events (n = 439, 52.4%) and cumulative number of embryos transferred (n = 326, 38.9%) (Fig. 4). The number of specifiers used to define RIF ranged from 0 to 9, with a median of 3 and standard deviation 1.8 (Supplementary Fig. S2). On analysis of all combinations of specifiers applied, RIF was most commonly defined by a minimum number of embryo transfer events or a minimum number of stimulated cycles alone (Fig. 5).

**Table 2.** Analysis of criteria used in RIF definitions provided by included studies.

Criteria used to define RIF	Outcome	n (%)	Range	Mean	Median	SD
***Min cumulative # embryos transferred***			2–15	5.0	4	2.7
	2 embryos	38 (4.5%)				
	3 embryos	37 (4.4%)				
	4 embryos	155 (18.5%)				
	≥5 embryos	96 (11.5%)				
	NR	512 (61.1%)				
***Min # embryo transfer events***			2–6	2.7	3	0.6
	2 transfers	157 (18.7%)				
	3 transfers	263 (31.4%)				
	≥4 transfers	19 (2.3%)				
	NR	399 (47.6%)				
***Min # embryos transferred per cycle***			1–4	1.4	1	0.7
	1 embryo	55 (6.6%)				
	2 embryos	17 (2.0%)				
	≥3 embryos	6 (0.7%)				
	NR	760 (90.7%)				
***Embryo developmental stage***						
	Cleavage only	59 (7.0%)				
	Blastocyst only	76 (9.1%)				
	Cleavage or blastocyst	36 (4.3%)				
	NR	667 (79.6%)				
***Embryo morphological quality***						
	Top quality	166 (19.8%)				
	At least good quality	319 (38.1%)				
	At least fair quality	5 (0.6%)				
	At least poor quality	1 (0.1%)				
	NR	347 (41.4%)				
***Embryo type (fresh, frozen)***						
	Fresh only	43 (5.1%)				
	Frozen only	9 (1.1%)				
	Fresh or frozen	153 (18.3%)				
	NR	633 (75.5%)				
***Embryo ploidy***						
	Euploid	10 (1.2%)				
	Not tested	1 (0.1%)				
	NR	827 (98.7%)				
***Min # stimulated cycles***						
	1 cycle	8 (1.0%)	1–6	2.6	3	0.7
	2 cycles	109 (13.0%)				
	3 cycles	150 (17.9%)				
	4 cycles	11 (1.3%)				
	≥5 cycles	3 (0.4%)				
	NR	557 (66.4%)				
***Consecutive failed ART cycles***						
	Required	92 (11.0%)				
	NR	746 (89.0%)				
***Maternal upper age limit (inclusive)***			30–49	39.0	39	2.4
	30–34	7 (0.8%)				
	35–39	105 (12.5%)				
	40–44	31 (3.7%)				
	45–49	4 (0.5%)				
	NR	691 (82.5%)				
***Good maternal ovarian/hormonal reserve***						
	Required	21 (2.5%)				
	NR	817 (97.5%)				
***Defined measure of failed implantation***						
	Lack of positive urine and/or serum hCG	64 (7.6%)				
	Lack of visible gestational sac on US	12 (1.4%)				
	Lack of clinical pregnancy	138 (16.5%)				
	Lack of viable pregnancy/delivery	3 (0.4%)				
	NR	621 (74.1%)				
***Additional specifiers (e.g. idiopathic RIF, endometrial thickness at implantation, etc.)***						
	Reported	130 (15.5%)				
	NR	708 (84.5%)				

NR, not reported; RIF, recurrent implantation failure; #, number of.

### Common RIF definitions

Of the 838 RIF definitions retrieved from eligible studies, there were a total of 503 unique RIF definitions. RIF was most frequently diagnosed as ‘failure of ≥3 transfer events’, occurring 26 times, followed by ‘failure of ≥3 stimulated cycles’ (22 times) and ‘failure to achieve clinical pregnancy after transfer of at least four good quality embryos in a minimum of three fresh or frozen transfer events in a woman of age <40 years’ (19 times). Other repeated RIF definitions included ‘failure of ≥2 transfer events’ (18 times), ‘failure of ≥3 transfer events with at least good quality embryos’ (14 times), and ‘failure of ≥3 stimulated cycles top quality embryos’ (14 times).

### Subgroup analyses

Subgroup analysis of RIF definitions was conducted hierarchically based on female age (less than 40 years) and embryo developmental stage (cleavage- versus blastocyst-only embryos), as detailed in Table 3. RIF definition criteria analysed included minimum cumulative number of embryos transferred, minimum number of embryo transfer events, minimum number of stimulated cycles, embryo developmental stage, and embryo morphological quality.

**Table 3.** Subgroup analysis of criteria used in RIF definitions provided by included studies.

Criteria used to define RIF	Outcome	n (%)	Range	Mean	Median	SD
***Female age <40 years (n = 112)***						
Min cumulative # embryos transferred			2–11	4.2	4	1.8
	2 embryos	7 (6.3%)				
	3 embryos	5 (4.5%)				
	4 embryos	55 (49.1%)				
	≥5 embryos	6 (5.4%)				
	NR	39 (34.8%)				
Min # embryo transfer events			2–6	2.9	3	0.7
	2 transfers	18 (16.1%)				
	3 transfers	52 (46.4%)				
	≥4 transfers	5 (4.5%)				
	NR	37 (33.0%)				
Min # stimulated cycles			2–4	2.7	3	0.6
	2 cycles	10 (8.9%)				
	3 cycles	15 (13.4%)				
	4 cycles	2 (1.8%)				
	NR	85 (75.9%)				
Embryo developmental stage						
	Cleavage only	6 (5.4%)				
	Blastocyst only	10 (8.9%)				
	Cleavage or blastocyst	5 (4.5%)				
	NR	91 (81.2%)				
Embryo morphological quality						
	Top quality	25 (22.3%)				
	At least good quality	53 (47.3%)				
	NR	34 (30.4%)				
Defined measure of implantation failure						
	Lack of positive urine or serum hCG	1 (0.9%)				
	Lack of visible gestational sac on US	2 (1.8%)				
	Lack of clinical pregnancy	46 (41.1%)				
	NR	63 (56.2%)				
***Cleavage only embryos (n = 59)***						
Min cumulative # embryos transferred			4–11	5.9	4	2.6
	4–5 embryos	28 (47.4%)				
	6–7 embryos	4 (6.7%)				
	8–9 embryos	7 (11.9%)				
	≥10 embryos	8 (13.6%)				
	NR	12 (20.4%)				
Min # embryo transfer events			2–4	2.5	2	0.7
	2 transfers	19 (32.2%)				
	3 transfers	9 (15.3%)				
	4 transfers	3 (5.1%)				
	NR	28 (47.4%)				
Min # stimulated cycles			1–4	2.3	2	0.6
	1 cycle	1 (1.7%)				
	2 cycles	15 (25.4%)				
	3 cycles	5 (8.5%)				
	4 cycles	1 (1.7%)				
	NR	37 (62.7%)				
Embryo morphological quality						
	Top quality	11 (18.6%)				
	At least good quality	40 (67.8%)				
	NR	8 (13.6%)				
Defined measure of failed implantation						
	Lack of positive urine and/or serum hCG	19 (32.2)				
	Lack of clinical pregnancy	13 (22.0%)				
	NR	27 (45.8%)				
***Blastocyst only embryos (n = 76)***						
Min cumulative # embryos transferred			2–4	2.7	3	0.8
	2 embryos	25 (32.9%)				
	3 embryos	14 (18.4%)				
	4 embryos	12 (15.8%)				
	NR	25 (32.9%)				
Min # embryo transfer events			2–6	2.6	2	0.8
	2 transfers	30 (39.5%)				
	3 transfers	22 (29.0%)				
	≥4 transfers	4 (5.2%)				
	NR	20 (26.3%)				
Min # stimulated cycles			1–3	2.5	3	0.8
	1 cycle	1 (1.3%)				
	2 cycles	2 (2.6%)				
	3 cycles	5 (6.6%)				
	NR	68 (89.5%)				
Embryo morphological quality						
	Top quality	21 (27.6%)				
	At least good quality	35 (46.1%)				
	NR	20 (26.3%)				
Defined measure of implantation failure						
	Lack of positive urine and/or serum hCG	8 (10.5%)				
	Lack of clinical pregnancy	18 (23.7%)				
	NR	50 (65.8%)				

NR, not reported; RIF, recurrent implantation failure; US, ultrasound; #, number of.

One hundred and twelve RIF definitions included only females less than 40 years. Of these definitions, the minimum cumulative number of embryos transferred ranged from 2 to 11 embryos with a median of four embryos transferred (interquartile range (IQR)=0) and was not reported in 39 definitions. For cleavage-stage embryos only (59 definitions), the minimum cumulative number of embryos transferred ranged from 4 to 11 embryos with a median of four embryos (IQR = 4) and was not reported in 12 definitions. For blastocyst-stage embryos only (76 definitions), the minimum cumulative number of embryos transferred ranged from two to four embryos, with a median of three embryos (IQR = 1) and was not reported in 25 definitions.

### Regression analyses

Over time, studies were significantly more likely to use both fresh and frozen embryo transfers for RIF definition (RRR = 1.23, 95% CI 1.13–1.33, *P* < 0.001) compared to the use of only fresh embryo transfers (Table 4). There was no significant association between the year of publication and the number of studies using cleavage or blastocyst-stage embryo transfers in their RIF definition. Over time, significantly fewer studies originated from Europe, North America, Oceania, and South America compared to studies originating from Asia (Table 4).

**Table 4.** Multinomial regression analysis of factors associated with year of publication.

Variable	Relative risk ratio (95% CI)	*P*-value
***Embryo developmental stage***	***Year of publication***	
Blastocyst only (reference)		
Cleavage-stage only	0.945 (0.868–1.028)	0.189
***Embryo type***	***Year of publication***	
Fresh embryo transfer only (reference)		
Frozen embryo transfer only	1.425 (1.069–1.900)	0.016*
Fresh or frozen embryo transfer	1.225 (1.130–1.328)	<0.001*
***Region of origin***	***Year of publication***	
Asia (reference)		
Africa	0.984 (0.874–1.106)	0.783
Europe	0.914 (0.885–0.944)	<0.001*
North America	0.859 (0.813–0.907)	<0.001*
		
Oceania	0.843 (0.772–0.920)	<0.001*
South America	0.877 (0.805–0.955)	0.003*

* *P* < 0.05.

The threshold for defining RIF according to the cumulative number of embryos transferred was significantly higher for cleavage-stage embryos compared to blastocysts (IRR = 2.15, 95% CI 1.76–2.64, *P* < 0.001) (Table 5). No significant associations were detected between: (i) the year of publication and number of specifiers included in definitions used, (ii) the developmental stage of embryos transferred and the minimum number of embryo transfer events to meet the definition of RIF, and (iii) the developmental stage of embryos transferred and the minimum number of stimulated cycles required to meet the definition of RIF (Table 5).

**Table 5.** Poisson regression analyses between year of publication and number of specifiers of the RIF definitions, as well as between embryo developmental stage and (a) the minimum number of embryos transferred cumulatively, (b) the minimum number of embryo transfer events, and (c) the minimum number of stimulated cycles used to define RIF.

Variable	Incidence rate ratio (95% CI)	*P*-value
	***Number of specifiers used to define RIF***	
***Year of publication***	1.006 (0.998–1.013)	0.166
***Embryo developmental stage***	***Minimum number of embryos transferred cumulatively to define RIF***	
Blastocyst (reference)		
Cleavage-stage	2.155 (1.759–2.640)	<0.001*
	***Minimum number of embryo transfer events to define RIF***	
Blastocyst (reference)		
Cleavage-stage	0.959 (0.728–1.265)	0.768
	***Minimum number of stimulated cycles to define RIF***	
Blastocyst (reference)		
Cleavage-stage	0.909 (0.541–1.527)	0.719

RIF, recurrent implantation failure.

* *P* < 0.05.

### Prognostic models

Based on the four models we proposed to assess the prognostic profile of patients included in the published studies, the proportion of RIF definitions that met the ESHRE-recommended RIF diagnostic threshold of >60% cumulative likelihood of pregnancy (ESHRE Working Group on Recurrent Implantation Failure *et al.*, 2023) was 2.1% for Model 1, 34.5% for Model 2, 6.4% for Model 3, and 65.1% for Model 4 (Table 6).

**Table 6.** Prognostic models estimating the cumulative likelihood of pregnancy.

***Prognostic model 1** ^a^ **(n = 838)***	
*Cumulative likelihood of pregnancy (%)*	*Frequency (% of total)*
27.8	494 (59.0)
38.6	209 (24.9)
45.1	50 (6.0)
47.8	9 (1.1)
53.1	5 (0.6)
55.6	2 (0.2)
59.3	51 (6.1)
62.3	3 (0.4)
69.9	2 (0.2)
77.7	1 (0.1)
78	1 (0.1)
82.4	7 (0.9)
89.7	1 (0.1)
92.6	2 (0.2)
95.4	1 (0.1)

**Prognostic model 2** ^b^ **(n = 838)**	

*Cumulative likelihood of pregnancy (%)*	*Frequency (% of total)*
53.1	549 (65.5)
67.9	260 (31.0)
78	12 (1.4)
84.9	3 (0.4)
89.7	4 (0.5)
90	7 (0.8)
96.8	3 (0.4)

**Prognosis model 3** ^c^ **(n = 94)**	

*Cumulative likelihood of pregnancy (%)*	*Frequency (% of total)*
27.8	9 (9.6)
38.6	8 (8.5)
45.1	16 (17.0)
47.8	2 (2.1)
53.1	2 (2.1)
59.3	51 (54.2)
69.9	2 (2.1)
77.7	1 (1.1)
78	1 (1.1)
89.7	1 (1.1)
95.4	1 (1.1)

**Prognostic model 4** ^d^ **(n = 439)**	

*Cumulative likelihood of pregnancy (%)*	*Frequency (% of total)*
53.1	153 (34.9)
67.9	260 (59.2)
78	12 (2.7)
84.9	3 (0.7)
89.7	4 (0.9)
90	4 (0.9)
96.8	3 (0.7)

a Prognostic model 1: ‘Worst case scenario’—if not otherwise specified, maternal age was assumed to be ≥40 years and number of embryo transfers was assumed to be 2.

b Prognostic model 2: ‘Optimistic theoretical cohort’—maternal age was assumed <35 years and if not otherwise specified, the number of embryo transfers was assumed to be 2.

c Prognostic model 3: ‘Available data model’—only studies reporting maximum maternal age and minimum number of embryos transferred were included.

d Prognostic model 4: ‘Theoretical minimum embryo transfer’ model—maternal age was assumed to be <35 years for all studies reporting a minimum number of embryo transfers.

Cumulative likelihood of pregnancy based on aggregate data published by ESHRE (ESHRE Working Group on Recurrent Implantation Failure *et al.*, 2023).

## Discussion

RIF has been a major challenge for patients and clinicians since the early days of ART and substantial research efforts have been made to identify relevant therapeutic interventions. This systematic review analysed the research output regarding RIF, clearly demonstrating the increasing interest in this topic over the past decade. Importantly this systematic review showcased the marked variability in the definition of RIF and the evolution of the use of different components and descriptors over the last four decades.

The global interest in RIF is demonstrated by the widespread distribution of available studies originating from 55 different countries. Interestingly, Asia has had a major contribution in this field over the past decade, contributing annually more than 70% of published research since 2014 (Supplementary Fig. S1).

Despite the widely acknowledged importance of RIF, no consistent or internationally accepted clinical definition of RIF currently exists and RIF is not yet recognized in the International Committee Monitoring Assisted Reproductive Technologies (ICMART) glossary (Zegers-Hochschild *et al.*, 2017). While most studies (91.5%) provided some form of RIF definition, the majority of these definitions (62.3%) were described using terminology that was ambiguous or lacked clarity, requiring assumptions to be made (Supplementary File S1). For example, by using terms such as ‘IVF-embryo transfer/ICSI-embryo transfer attempts’ of ‘ART cycles’ without specifying whether this refers to the number of stimulation cycles or embryo transfer events, there exists potential disparity between the intended versus interpreted RIF definition. It should be noted also that the term ‘RIF’ is often erroneously used synonymously with ‘recurrent/repeated IVF failure’, the latter of which incorporates other causes of lack of clinical pregnancy/live birth following ART such as recurrent miscarriage, poor response to ovarian stimulation, poor embryo quality or development etc.—this further contributes to the confusion surrounding the definition of RIF.

Even more concerning though is the striking variability in the definitions of RIF used in the literature. Out of the 838 definitions used in the 748 reports analysed, there were 503 unique definitions reported, which renders the comparison of different interventions for RIF and the synthesis of available evidence an impossible task. This creates significant issues around the interpretation of the available literature, particularly as it pertains to the population sampled by each study, limiting its external validity (i.e. generalizability). This greatly diminishes the value of published research and represents an unfortunate waste of resources with minimal, if any, translational value. It is also not surprising that most studies (67.6%) did not explain how they formulated their definition of RIF, which raises of possibility of convenience sampling (while also permitting selection bias) and further devalues the available evidence.

The need for a homogeneous, *a priori* definition of conditions has been previously highlighted in the case of poor ovarian response in ART which eventually led to the introduction of the Bologna criteria (Ferraretti *et al.*, 2011) which, although not perfect, have represented a major step in the right direction. Similarly, recently the ESHRE working group on RIF recognized the need for a more standardized definition of RIF based on the cumulative probability of pregnancy depending on several prognostic factors such as female age, the developmental stage of the embryos (cleavage versus blastocyst), embryo ploidy, and the number of embryos transferred (Coughlan *et al.*, 2014; Ata *et al.*, 2021; Rozen *et al.*, 2021; ESHRE Working Group on Recurrent Implantation Failure *et al.*, 2023; Pirtea *et al.*, 2023).

In that respect, this systematic review identified that the most common specifier used in the definition of RIF was embryo morphological quality. This underlines the importance researchers place on the prognostic value of embryo morphology, which has been shown to have a correlation with embryo ploidy in blastocysts (Capalbo *et al.*, 2014) and even with the probability of ongoing pregnancy in case of euploid blastocysts (Irani *et al.*, 2017). Another important determinant of success is the developmental stage of the embryo, however, this seems to have been underutilized in the definitions of RIF in the literature. The most plausible explanation is the fact that extended blastocyst culture has been introduced only in the last two decades and has had variable uptake in certain areas of the world, particularly in Asia (Kushnir *et al.*, 2017; Glujovsky *et al.*, 2022). This is also the likely reason behind the infrequent use of the number of embryos transferred per embryo transfer event in the definition of RIF. Single embryo transfer has also been popularized in the last decade (particularly along with the establishment of blastocyst culture) (van Montfoort *et al.*, 2006; Practice Committee of the American Society for Reproductive Medicine, 2021), and, for this reason, the transfer of multiple cleavage stage embryos per transfer event has been the norm. Therefore, most researchers have opted for the use of cumulative number of embryos transferred, and this indeed has been found to be higher for cleavage-stage embryos compared to blastocysts.

Another important determinant of pregnancy achievement is embryo ploidy which has been repeatedly shown to be superior to embryo morphology (Yang *et al.*, 2012; Capalbo *et al.*, 2014; Munné *et al.*, 2019). The implantation potential of a euploid blastocyst has been reported to range between 50% and 70% (Forman *et al.*, 2013) and therefore the probability of not having achieved a pregnancy after the sequential transfer of three euploid blastocysts has been shown to be <7% (Pirtea *et al.*, 2021). Recently, an updated analysis suggested that the transfer of up to five euploid blastocysts (Gill *et al.*, 2023) leads to >98% cumulative probability of pregnancy. Alongside aneuploidy, the Pirtea *et al.* (2021) and Gill *et al.* (2023) studies both excluded other known factors affecting implantation potential, including intrauterine pathology and advanced maternal age (>45 years), to investigate the existence and estimate the prevalence of otherwise unexplained RIF. For this reason, it has been recently argued that in order to limit the burden of a false diagnosis, RIF should only be diagnosed after known causes of implantation failure have been investigated and excluded or, if euploidy status is unknown, after the cumulative probability of implantation has been calculated from an age-adjusted equivalent number of untested blastocysts (Ata *et al.*, 2021; Pirtea *et al.*, 2023).

Considering the lack of universal availability of PGT, its associated costs and the concerns that have been raised regarding embryo biopsy and the ability of PGT to enhance the cumulative chance of pregnancy, researchers have suggested introducing female age as a surrogate predictor of the probability of embryo euploidy (Ata *et al.*, 2021). Unfortunately, this has been used as a component of the RIF definition in less than one in seven of the studies. Recently proposed models to define RIF have incorporated female age (Coughlan *et al.*, 2014; Ata *et al.*, 2021; ESHRE Working Group on Recurrent Implantation Failure *et al.*, 2023) which is likely to reduce overdiagnosis and increase homogeneity in the population examined.

The diagnosis of RIF on the basis of prognosis has been a subject of debate recently, where several authors have proposed different cut-offs of cumulative chance of pregnancy over which diagnostic and therapeutic interventions are justified. Venetis and Kolibianakis (2019) proposed a cut off of 85%, Rozen *et al.* (2021) suggested a cut-off of 80%, and Ata *et al.* (2021) proposed a cut-off of 95%. ESHRE, following polling among the members of the working group, suggested a more relaxed definition by proposing a cut-off of 60%. All these cut-offs are arbitrary in nature and proper studies, including health economic assessments, need to be performed to ascertain what is the cut-off that has the best sensitivity and specificity to identify populations that benefit from further diagnostic and therapeutic interventions or their inclusion in clinical research.

One of the most revealing aspects of this systematic review is the assessment of the prognostic profile of the population included in each publication. Based on this analysis, for three out of four scenarios, very few of the studies satisfy the criteria of the RIF population according to the >60% cut-off proposed by ESHRE (ESHRE Working Group on Recurrent Implantation Failure *et al.*, 2023). This essentially could be translated to significant overdiagnosis of RIF both in previously published research and therefore, in everyday clinical practice. Further to the potential inappropriate use of diagnostic and therapeutic interventions in populations that do not need them, testing an intervention that might be beneficial for RIF in a sample of patients where very few are truly RIF will likely lead to effect dilution and therefore failure to demonstrate the benefit of the intervention (Ata *et al.*, 2021).

This systematic review is also characterized by limitations that need to be discussed. Abstracts and case-series were excluded as they were considered to provide limited information regarding RIF definition. Most importantly, even by including only published manuscripts, several studies had limited data and required certain assumptions to be made. Even though these were made on the basis on predetermined criteria to ensure consistency and reproducibility, the potential for misclassification cannot be excluded. For this reason, in the case of ascertaining the prognostic profile of the populations examined based on the reported definitions, several scenarios were utilized in order to evaluate the robustness of our findings when the assumptions made varied.

## Conclusion

In conclusion, the present systematic review clearly shows the clinical conundrum that RIF has been over the past few decades and the complete lack of a homogeneous definition in the relevant research. This, along the fact that in most studies the included population does not meet the RIF criteria of ESHRE, should caution the readers in how they should interpret the published evidence for RIF diagnostic and therapeutic interventions and emphasize the need for future research on the basis of the recent, more standardized definitions proposed.

## Supplementary Material

hoaf033_Supplementary_Data

## Data Availability

All data supporting the findings of this study are available within the manuscript and its Supplementary Material.
