# Supplementary material for: Variability and implications of recurrent implantation failure definitions used in the scientific literature: a systematic review
Source: Hum Reprod Open. 2025 Jun 18;2025(3):hoaf033. doi: 10.1093/hropen/hoaf033 (PMC12321291; doi:10.1093/hropen/hoaf033)
Supplement: hoaf033_Supplementary_Data [file hoaf033_supplementary_data.zip › Supplementary File S1.docx]

**Supplementary File S1**

The following rules were applied when transcribing RIF definitions:

(1) To distinguish between transfer events and stimulation cycles:

(1a) ‘Implantation failures’, ‘transplantation failures’, ‘transmission failures’ and ‘nidation failures’ were interpreted as transfer events.

(1b) ‘Fresh cycles’, ‘frozen cycles’, ‘IVF/ICSI or frozen embryo replacement cycles’ were interpreted as transfer events.

(1c) ‘IVF-embryo transfer (IVF-ET) cycles/attempts’, ‘ART-transfers’, ‘in vitro fertilisation (IVF) cycles/programs’, ‘intracytoplasmic sperm injection (ICSI) cycles’, ‘ICSI-ET cycles’, ‘oocyte donation cycles/programs’, ‘ART cycles/attempts’, ‘oocyte collections’ were interpreted as stimulation cycles.

(1d) ‘Cycles’, ‘attempts’ (with no other specifiers) were interpreted as stimulation cycles.

(2) If distinction between the RIF definition and inclusion criteria was not clear (i.e. not clearly defined in the materials and methods, not able to be elucidated from the abstract or introduction), RIF was assumed to be equivalent to the inclusion criteria for the RIF group.

(3) Unless a cut-off value was specified, plurals (e.g. ‘failures’ or ‘embryos’ or ‘cycles’, including with quantifiers such as ‘multiple’ or ‘several’) were assumed to be ≥2.
