# Supplementary material for: Variability and implications of recurrent implantation failure definitions used in the scientific literature: a systematic review
Source: Hum Reprod Open. 2025 Jun 18;2025(3):hoaf033. doi: 10.1093/hropen/hoaf033 (PMC12321291; doi:10.1093/hropen/hoaf033)
Supplement: hoaf033_Supplementary_Data [file hoaf033_supplementary_data.zip › Supplementary File S2_EO.docx]

**Supplementary File S2** References for articles included in the systematic review

Abdulhaleem LS, Abdul-Rasheed OF, Al-Awadi SJA, and Abdullah TH. The association between angiopoietin-2 and the risk of recurrent implantation failure. *Research Journal of Pharmacy and Technology* 2021: **14(4)**; 2031-2034.

Abou-El-Naga AM, El-Sayyad H, Youssef HM, and Mosbah AA. Effect of platelet-rich plasma intrauterine infusion on pregnancy outcomes in Egyptian infertile women. *Bioscience Research* 2022: **19(1)**; 164-170.

Acet F, Sahin G, Goker ENT, and Tavmergen E. The effect of hysteroscopy and conventional curretage versus no hysteroscopy on live birth rates in recurrent in vitro fertilisation failure: a retrospective cohort study from a single referral centre experience. *Journal of Obstetrics and Gynaecology* 2022: **42(6)**; 2134-2138.

Acet F, Sahin G, Goker ENT, and Tavmergen E. Reproductive and ICSI outcomes after hysteroscopic metroplasty for T-shaped uterus, a prospective follow up study. *Journal of Gynecology Obstetrics and Human Reproduction* 2022: **51(8)**:102450.

Achache H, Tsafrir A, Prus D, Reich R, and Revel A. Defective endometrial prostaglandin synthesis identified in patients with repeated implantation failure undergoing in vitro fertilization. *Fertility and sterility* 2010: **94(4)**; 1271-1278.

Aghajanpour S, Hosseini E, Amirchaghmaghi E, Zandieh Z, Amjadi F, Yahyaei A, Zolfaghari Z, Aflatoonian K, Ashrafi M, and Aflatoonian R. Differential expression of innate/adaptive immunity genes induced by endometrial scratching as a hopeful approach for implantation boosting in unexplained, repeated implantation failure: An RCT. *Journal of reproductive immunology* 2021: **148**:103426; 9.

Aghajanpour S, Mehraein F, Amjadi F, Zandieh Z, Ghaffari F, Aflatoonian K, Hosseini E, Bakhtiyari M, and Aflatoonian R. Endometrial scratching in unexplained repeated implantation failure causes two competing forces, angiogenesis and anti-angiogenesis: An RCT study. *International journal of reproductive biomedicine* 2024: **22(4)**; 253-268.

Aghajanzadeh F, Esmaeilzadeh S, Basirat Z, Mahouti T, Heidari FN, and Golsorkhtabaramiri M. Using autologous intrauterine platelet-rich plasma to improve the reproductive outcomes of women with recurrent implantation failure. *Jornal brasileiro de reproducao assistida* 2020: **24(1)**; 30-33.

Ahmadi M, Abdolmohamadi-vahid S, Ghaebi M, Dolati S, Abbaspour-Aghdam S, Danaii S, Berjis K, Madadi-Javid R, Nouri Z, Siahmansouri H*, et al.* Sirolimus as a new drug to treat RIF patients with elevated Th17/Treg ratio: A double-blind, phase II randomized clinical trial. *International immunopharmacology* 2019: **74**:105730.

Ahmadi M, Abdolmohammadi-Vahid S, Ghaebi M, Aghebati-Maleki L, Dolati S, Farzadi L, Ghasemzadeh A, Hamdi K, Younesi V, Nouri M*, et al.* Regulatory T cells improve pregnancy rate in RIF patients after additional IVIG treatment. *Systems Biology in Reproductive Medicine* 2017: **63(6)**; 350-359.

Ahmadi M, Pashangzadeh S, Moraghebi M, Sabetian S, Shekari M, Eini F, Salehi E, and Mousavi P. Construction of circRNA-miRNA-mRNA network in the pathogenesis of recurrent implantation failure using integrated bioinformatics study. *Journal of Cellular and Molecular Medicine* 2021: **26(6)**; 1853-1864.

Al-Lamee H, Ellison A, Drury J, Hill CJ, Drakeley AJ, Hapangama DK, and Tempest N. Altered endometrial oestrogen-responsiveness and recurrent reproductive failure. *Reprod Fertil* 2022: **3(1)**; 30-38.

Al-Turki HA. Hysteroscopy as an investigation tool in recurrent implantation failure in vitro fertilization. *Saudi Medical Journal* 2018: **39(3)**; 243-246.

Al-Zebeidi J, Agdi M, Lary S, Al-Obaid S, Salim G, and Al-Jaroudi D. Effect of empiric intravenous intralipid therapy on pregnancy outcome in women with unexplained recurrent implantation failure undergoing intracytoplasmic sperm injection-embryo transfer cycle: a randomized controlled trial. *Gynecological endocrinology* 2020: **36(2)**; 131-134.

Alaee S, Ghaffari Novin M, Noroozian M, Yeganeh F, Pakravesh J, Heidari MH, and Salehpour S. Evaluation of progesterone receptor, FKBP51 and FKBP52, associated with uterine receptivity, in endometrial tissue of women with repeated implantation failure. *Acta Endocrinologica* 2014: **10(3)**; 329-339.

Albayrak İ G, Azhari F, Çolak EN, Balcı BK, Ülgen E, Sezerman U, Baştu E, and Günel T. Endometrial gene expression profiling of recurrent implantation failure after in vitro fertilization. *Mol Biol Rep* 2021: **48(6)**; 5075-5082.

Alecsandru D, Barrio A, Andia V, Cruz E, Aparicio P, Serna J, Cruz M, Pellicer A, and Garcia-Velasco JA. Pancreatic autoimmunity: An unknown etiology on patients with assisted reproductive techniques (ART)-recurrent reproductive failure. *PLoS One* 2018: **13(10)**; e0203446.

Alecsandru D, Garrido N, Vicario JL, Barrio A, Aparicio P, Requena A, and García-Velasco JA. Maternal KIR haplotype influences live birth rate after double embryo transfer in IVF cycles in patients with recurrent miscarriages and implantation failure. *Human Reproduction* 2014: **29(12)**; 2637-2643.

Alecsandru D, López-Palacios N, Castaño M, Aparicio P, García-Velasco JA, and Núñez C. Exploring undiagnosed celiac disease in women with recurrent reproductive failure: The gluten-free diet could improve reproductive outcomes. *American journal of reproductive immunology* 2020: **83 (2)**; e13209.

Aletebi F. Hysteroscopy in women with implantation failures after in vitro fertilization: Findings and effect on subsequent pregnancy rates. *Middle east fertility society journal* 2010: **15(4)**; 288-291.

Allafan S, Nazarabadi MH, Enghelabifar M, Khayatzadeh J, Abadi KS, Jalali M, Moradi F, Musavifar N, Reza JZ, and Mojarrad M. No association between recurrent implantation failure (RIF) following in vitro fertilization (IVF) with gene polymorphism (P53 Arg72pro). *Iranian Journal of Obstetrics, Gynecology and Infertility* 2015: **18(172)**; 18-25.

Altmae S, Mendoza-Tesarik R, Mendoza C, Mendoza N, Cucinelli F, and Tesarik J. Effect of growth hormone on uterine receptivity in women with repeated implantation failure in an oocyte donation program: a randomized controlled trial. *Journal of the endocrine society* 2018: **2(1)**; 96‐105.

Altmäe S, Tamm-Rosenstein K, Esteban FJ, Simm J, Kolberg L, Peterson H, Metsis M, Haldre K, Horcajadas JA, Salumets A*, et al.* Endometrial transcriptome analysis indicates superiority of natural over artificial cycles in recurrent implantation failure patients undergoing frozen embryo transfer. *Reproductive biomedicine online* 2016: **32(6)**; 597-613.

Amin J, Sr., Patel R, JayeshAmin G, Gomedhikam J, Surakala S, and Kota M. Personalized Embryo Transfer Outcomes in Recurrent Implantation Failure Patients Following Endometrial Receptivity Array With Pre-Implantation Genetic Testing. *Cureus Journal of Medical Science* 2022: **14(6)**:e26248; 8.

Amini M, Ranjkesh M, Nikanfar S, Fattahi A, Farzadi L, and Hamdi K. Alterations of uterine blood flow during the follicular phase in patients with recurrent implantation failure: A doppler ultrasonographic study. *International Journal of Women's Health and Reproduction Sciences* 2021: **9(3)**; 217-221.

Amjadi F, Zandieh Z, Mehdizadeh M, Aghajanpour S, Raoufi E, Aghamajidi A, and Aflatoonian R. The uterine immunological changes may be responsible for repeated implantation failure. *Journal of reproductive immunology* 2020: **138**:103080; 4.

Arefi S, Ataei M, Maleki N, Yari N, Razi S, and Amirajam S. Sequential (two-step) day 3/day 5 frozen-thawed embryo transfer: does it improve the pregnancy rate of patients suffering recurrent implantation failure? *Journal of medicine and life* 2022: **15(11)**; 1365-1370.

Arefi S, Hoseini A, Farifteh F, and Zeraati H. Modified natural cycle frozen-thawed embryo transfer in patients with repeated implantation failure: An observational study. *International journal of reproductive biomedicine* 2016: **14(7)**; 465-470.

Armine T, Manik G, and Eduard H. Single and double endometrial scratching (ES) in infertile women with strict criteria of recurrent implantation failure (RIF). *Gynecological endocrinology* 2019: **35(sup1)**; 11-14.

Asgari F, Gavahi A, Karimi M, Vatannejad A, Amjadi F, Aflatoonian R, and Zandieh Z. Risk of embryo aneuploidy is affected by the increase in sperm DNA damage in recurrent implantation failure patients under ICSI-CGH array cycles. *Human fertility* 2021; 1-9.

Aslan D, Elizur SE, Levron J, Shulman A, Lerner-Geva L, Bider D, and Dor J. Comparison of zygote intrafallopian tube transfer and transcervical uterine embryo transfer in patients with repeated implantation failure. *European journal of obstetrics, gynecology, and reproductive biology* 2005: **122(2)**; 191-194.

Aslan K, Kasapoglu I, Cinar C, Cakir C, Avci B, and Uncu G. Low Molecular Weight Heparin-Aspirin-Prednisolone Combination Does Not Increase the Live Birth Rate in Recurrent Implantation Failure: A Retrospective Cohort Study. *Reproductive Sciences* 2023: **30(11)**; 3253-3260.

Aydeniz EG, Sari U, and Dilek TUK. Pregnancy success rate at recurrent implantation failure patients after hysteroscopic endometrial injury: preliminary study. *Clinical and Experimental Obstetrics and Gynecology* 2019: **46(5)**; 709-712.

Azarpoor A, Ardeshirylajimi A, Yeganeh SM, Matrood EP, Dehghan Z, and Salehi M. The expression of mir-31 and its target gene foxp3 in recurrent implantation failure patients. *International Journal of Women's Health and Reproduction Sciences* 2020: **8(4)**; 389-395.

Azarpoor A, Ardeshirylajimi A, Yeganeh SM, Pourmatrood E, Dehghan Z, Nobijari FF, and Salehi M. Expressions of miR-199a-5p and miR-125b-5p and their target genes in the endometrium of recurrent implantation failure patients follow-ing in uterus infusion of autologous peripheral blood mononuclear cells. *Trends in Immunotherapy* 2022: **6(2)**; 50-63.

Azhari F, Pence S, Hosseini MK, Balci BK, Cevik N, Bastu E, and Gunel T. The role of the serum exosomal and endometrial microRNAs in recurrent implantation failure. *Journal of Maternal-Fetal & Neonatal Medicine* 2020; 11.

Azhari F, Pence S, Hosseini MK, Balci BK, Cevik N, Bastu E, and Gunel T. The role of the serum exosomal and endometrial microRNAs in recurrent implantation failure. *Journal of Maternal-Fetal and Neonatal Medicine* 2022: **35(5)**; 815-825.

Azouz YS, Eid MA, Shehata MR, and El-Rahman HAA. The Effect Of Culture System On Embryonic Development and Aneuploidy Rate For Icsi Cases. *Egyptian Journal of Hospital Medicine* 2021: **85(2)**:86; 3960-3966.

Babayeva G, Purut YE, Giray B, Oltulu P, Alakuş R, and Çolakoğlu MC. Endometrial CD56+ natural killer cells in women with recurrent implantation failure: An immunohistochemical study. *Turk J Obstet Gynecol* 2020: **17(4)**; 236-239.

Babian S, Salehpour S, Nazari L, and Ghorbanmehr N. The expression level of mir-21-3p in platelet-rich plasma: A potential effective factor and predictive biomarker in recurrent implantation failure. *Molecular Reproduction and Development* 2022: **89(10)**; 498-505.

Bahar L, Kahraman S, Eras N, and Pirkevi C. Comparison of endometrial biopsies of fertile women and women with repeated implantation failure at the ultrastructural level. *Turk J Med Sci* 2015: **45(3)**; 706-713.

Bahar L, Kahraman S, Reşitoglu B, and Baykal T. Ultrastructural and immunohistochemical evaluation of endometrial tissues of infertile women with recurrent implantation failure. *Eastern Journal of Medicine* 2013: **18(1)**; 1-7.

Bahrami-Asl Z, Farzadi L, Fattahi A, Yousefi M, Quinonero A, Hakimi P, Latifi Z, Nejabati HR, Ghasemnejad T, Sadigh AR*, et al.* Tacrolimus Improves the Implantation Rate in Patients with Elevated Th1/2 Helper Cell Ratio and Repeated Implantation Failure (RIF). *Geburtshilfe Frauenheilkd* 2020: **80(8)**; 851-862.

Bakhsh AS, Maleki N, Sadeghi MR, SadeghiTabar A, Tavakoli M, Zafardoust S, Karimi A, Askari S, Jouhari S, and Mohammadzadeh A. Effects of Autologous Platelet-Rich Plasma in women with repeated implantation failure undergoing assisted reproduction. *JBRA Assist Reprod* 2021.

Bakhsh AS, Maleki N, Sadeghi MR, SadeghiTabar A, Tavakoli M, Zafardoust S, Karimi A, Askari S, Jouhari S, and Mohammadzadeh A. Effects of Autologous Platelet-Rich Plasma in women with repeated implantation failure undergoing assisted reproduction. *JBRA assisted reproduction* 2021.

Bakry MS, Eldesouky E, Alghazaly MM, farag E, Sultan EEK, Elazzazy H, Mohamed A, Ali SMS, Anwar A, Elrashedy AA*, et al.* Granulocyte colony stimulating factor versus human chorionic gonadotropin for recurrent implantation failure in intra cytoplasmic sperm injection: a randomized clinical trial. *BMC Pregnancy and Childbirth* 2022: **22(1)**:881.

Ban Y, Yang X, Xing Y, Que W, Yu Z, Gui W, Chen Y, and Liu X. Intrauterine Infusion of Leukocyte-Poor Platelet-Rich Plasma Is an Effective Therapeutic Protocol for Patients with Recurrent Implantation Failure: A Retrospective Cohort Study. *Journal of Clinical Medicine* 2023: **12(8)**; 2823.

Bansal R, Ford B, Bhaskaran S, Thum M, and Bansal A. Elevated Levels of Serum Vascular Endothelial Growth Factor-A Are Not Related to NK Cell Parameters in Recurrent IVF Failure. *J Reprod Infertil* 2017: **18(3)**; 280-287.

Bar G, Harlev A, Alfayumi-Zeadna S, Zeadna A, Bord I, Har-Vardi I, Lunenfeld E, and Levitas E. Recurrent implantation failure: which patients benefit from endometrial scratching prior to IVF? *Arch Gynecol Obstet* 2020: **301(3)**; 817-822.

Barri PN, Coroleu B, Clua E, Tur R, Boada M, and Rodriguez I. Investigations into implantation failure in oocyte-donation recipients. *Reprod Biomed Online* 2014: **28(1)**; 99-105.

Barritt JA, Brenner CA, Willadsen S, and Cohen J. Spontaneous and artificial changes in human ooplasmic mitochondria. *Hum Reprod* 2000: **15 Suppl 2**; 207-217.

Basatvat S, Russell JM, Saare M, Thurston LM, Salumets A, and Fazeli A. Potential innate immunity-related markers of endometrial receptivity and recurrent implantation failure (RIF). *Reprod Biol* 2021: **21(4)**; 100569.

Basirat Z, Kashifard M, Aghaei Z, Mahouti T, Jorsaraei SGA, and Golsorakhtabar Amiri M. Assessment of effective factors in recurrent implantation failure (RIF) following assisted reproductive technology (ART). *Journal of Babol University of Medical Sciences* 2019: **21(1)**; 383-389.

Bastu E, Demiral I, Gunel T, Ulgen E, Gumusoglu E, Hosseini MK, Sezerman U, Buyru F, and Yeh J. Potential Marker Pathways in the Endometrium That May Cause Recurrent Implantation Failure. *Reprod Sci* 2019: **26(7)**; 879-890.

Bastu E, Mutlu MF, Yasa C, Dural O, Aytan AN, Celik C, Buyru F, and Yeh J. Role of Mucin 1 and Glycodelin A in recurrent implantation failure. *Fertility and sterility* 2015: **103(4)**; 1059-1064.e1052.

Baum M, Yerushalmi GM, Maman E, Kedem A, Machtinger R, Hourvitz A, and Dor J. Does local injury to the endometrium before IVF cycle really affect treatment outcome? Results of a randomized placebo controlled trial. *Gynecological endocrinology* 2012: **28(12)**; 933‐936.

Bayati F, Eftekhar M, Homayoon N, and Fatehi H. Comparison of Doppler ultrasound indices of uterine artery and sub endometrial blood supply in frozen embryo transfer with and without repeated implantation failure: A cross-sectional study. *International journal of reproductive biomedicine* 2023: **21(11)**; 937-942.

Baybordi E, Mohseni J, and Mosapour P. The effect of platelet-rich plasma on the improvement of pregnancy results in repeated implantation failure: A randomized controlled trial. *International journal of reproductive biomedicine* 2022: **20(9)**; 753-760.

Benkhalifa M, Demirol A, Sari T, Balashova E, Tsouroupaki M, Giakoumakis Y, and Gurgan T. Autologous embryo-cumulus cells co-culture and blastocyst transfer in repeated implantation failures: a collaborative prospective randomized study. *Zygote (Cambridge, England)* 2012: **20(2)**; 173‐180.

Benkhalifa M, Zidi W, Bahri H, Mahjoub S, Boudhraa K, Sanhaji H, Khorsi-Cauet H, Feki M, Benkhalifa M, and Allal-Elasmi M. Circulating MMP-7 and VEGF as potential predictive biomarkers for recurrent implantation failures. *Zygote* 2021: **29(5)**; 365-371.

Berdiaki A, Vergadi E, Makrygiannakis F, Vrekoussis T, and Makrigiannakis A. Repeated implantation failure is associated with increased Th17/Treg cell ratio, during the secretory phase of the human endometrium. *Journal of reproductive immunology* 2024: **161**:104170.

Berkay EG, Şoroğlu CV, Kalaycı T, Uyguner ZO, Akçapınar GB, and Başaran S. A new enrichment approach for candidate gene detection in unexplained recurrent pregnancy loss and implantation failure. *Molecular Genetics and Genomics* 2023: **298(1)**; 253-272.

Berker B, Taşkin S, Kahraman K, Taşkin EA, Atabekoğlu C, and Sönmezer M. The role of low-molecular-weight heparin in recurrent implantation failure: a prospective, quasi-randomized, controlled study. *Fertility and sterility* 2011: **95(8)**; 2499‐2502.

Berkhout RP, Lambalk CB, Repping S, Hamer G, and Mastenbroek S. Premature expression of the decidualization marker prolactin is associated with repeated implantation failure. *Gynecol Endocrinol* 2020: **36(4)**; 360-364.

Best JC, Kohn T, Patel P, Blachman-Braun R, de Quadros E, Beyhan Z, Jacobs M, and Ramasamy R. Elevated sperm DNA fragmentation does not predict recurrent implantation failure. *Andrologia* 2021: **53(7)**; e14094.

Bielfeld AP, Pour SJ, Poschmann G, Stühler K, Krüssel JS, and Baston-Büst DM. A proteome approach reveals differences between fertile women and patients with repeated implantation failure on endometrial level—Does hCG render the endometrium of RIF patients? *International Journal of Molecular Sciences* 2019: **20(2)**:425.

Bildaci TB, Haydardedeoglu B, Karakaya BK, Bolat FA, and Zeyneloglu HB. The importance of CD56 and CD98 levels in patients with recurrent implantation failure. *Journal of Clinical and Analytical Medicine* 2017: **8(3)**; 216-218.

Bilgory A, Shalom-Paz E, Atzmon Y, Aslih N, Shibli Y, Estrada D, and Haimovich S. Diode Laser Hysteroscopic Metroplasty for Dysmorphic Uterus: a Pilot Study. *Reproductive Sciences* 2022: **29(2)**; 506-512.

Blazheva S, Pachkova S, Bodurska T, Ivanov P, Blazhev A, Lukanov T, and Konova E. Unlocking the Uterine Code: Microbiota, Immune Cells, and Therapy for Recurrent Reproductive Failure. *Microorganisms* 2024: **12(3)**:547.

Blázquez A, García D, Rodríguez A, Vassena R, and Vernaeve V. Use of donor sperm in addition to oocyte donation after repeated implantation failure in normozoospermic patients does not improve live birth rates. *Hum Reprod* 2016: **31(11)**; 2549-2553.

Blessmann-Roset J, Rives N, Clavier B, Milazzo JP, Mazurier S, Mousset-Siméon N, and MacÉ B. Laser assisted hatching: Rouen University Hospital outcomesé valuation des indications au CHU de Rouen. *Gynecologie Obstetrique et Fertilite* 2009: **37(4)**; 313-320.

Blockeel C, Schutyser V, De Vos A, Verpoest W, De Vos M, Staessen C, Haenti̧ens P, Van der Elst J, and Devroey P. Prospectively randomized controlled trial of PGS in IVF/ICSI patients with poor implantation. *Reproductive biomedicine online* 2008: **17(6)**; 848-854.

Bord I, Tamir B, Harlev A, Har-Vardi I, Lunenfeld E, Friger M, and Levitas E. Recurrent implantation failure in IVF: features of cycles that eventually ended in conception. *Arch Gynecol Obstet* 2016: **293(4)**; 893-900.

Boudjenah R, Molina-Gomes D, Wainer R, De Mazancourt P, Selva J, and Vialard F. The vascular endothelial growth factor (VEGF) +405 G/C polymorphism and its relationship with recurrent implantation failure in women in an IVF programme with ICSI. *Journal of assisted reproduction and genetics* 2012: **29(12)**; 1415-1420.

Bouet PE, El Hachem H, Monceau E, Gariépy G, Kadoch IJ, and Sylvestre C. Chronic endometritis in women with recurrent pregnancy loss and recurrent implantation failure: prevalence and role of office hysteroscopy and immunohistochemistry in diagnosis. *Fertil Steril* 2016: **105(1)**; 106-110.

Braun AS, Vomstein K, Reiser E, Tollinger S, Kyvelidou C, Feil K, and Toth B. NK and T Cell Subtypes in the Endometrium of Patients with Recurrent Pregnancy Loss and Recurrent Implantation Failure: Implications for Pregnancy Success. *Journal of Clinical Medicine* 2023: **12(17)**:5585.

Brosens JJ, Hodgetts A, Feroze-Zaidi F, Sherwin JRA, Fusi L, Salker MS, Higham J, Rose GL, Kajihara T, Young SL*, et al.* Proteomic analysis of endometrium from fertile and infertile patients suggests a role for apolipoprotein A-I in embryo implantation failure and endometriosis. *Molecular Human Reproduction* 2009: **16(4)**; 273-285.

Bui BN, Kukushkina V, Meltsov A, Olsen C, van Hoogenhuijze N, Altmae S, Mol F, Teklenburg G, de Bruin JP, Besselink D*, et al.* The endometrial transcriptome of infertile women with and without implantation failure. *Acta Obstetricia et Gynecologica Scandinavica* 2024: **103(7)**; 1348-1365.

Busnelli A, Reschini M, Cardellicchio L, Vegetti W, Somigliana E, and Vercellini P. How common is real repeated implantation failure? An indirect estimate of the prevalence. *Reprod Biomed Online* 2020: **40(1)**; 91-97.

Çağlayan A, Horsanali MO, and Buyrukcu BA. The role of sperm DNA integrity in couples with recurrent implantation failure following IVF treatment. *Andrologia* 2022: **54(9)**; e14496.

Cai JY, Tang YY, Deng XH, Li YJ, Liang G, Meng YQ, and Zhou H. Recurrent Implantation Failure May Be Identified by a Combination of Diagnostic Biomarkers: An Analysis of Peripheral Blood Lymphocyte Subsets. *Frontiers in Endocrinology* 2022: **13**:865807.

Canan S, Inan MA, Erdem A, Demirdag E, Gunduz MI, Erdem O, and Erdem M. Evaluation of endometrial receptivity in recurrent pregnancy loss and recurrent implantation failure. *Turkish Journal of Obstetrics and Gynecology* 2024: **21(1)**; 22-27.

Canella PRBC, Vinces SS, Silva AAR, Sanches PHG, Barini R, Porcari ADM, Razolli DS, and Carvalho PDO. Altered profile of plasma phospholipids in woman with recurrent pregnancy loss and recurrent implantation failure treated with lipid emulsion therapy. *American journal of reproductive immunology* 2023: **89(3)**:e13673.

Cao Z, Wang X, Liu Y, Tang X, Wu M, Zhen X, Kang N, Ding L, Sun J, Cai X*, et al.* PRMT5 deficiency disturbs Nur77 methylation to inhibit endometrial stromal cell differentiation in recurrent implantation failure. *bioRxiv.* 2024:2024.02.06.579055.

Cao Z, Yan Q, Zhang M, Zhu Y, Liu J, Jiang Y, Zhen X, Xu M, Yue Q, Zhou J*, et al.* FHL1 mediates HOXA10 deacetylation via SIRT2 to enhance blastocyst-epithelial adhesion. *Cell Death Discov* 2022: **8(1)**; 461.

Cela V, Daniele S, Obino MER, Ruggiero M, Zappelli E, Ceccarelli L, Papini F, Marzi I, Scarfo G, Tosi F*, et al.* Endometrial Dysbiosis Is Related to Inflammatory Factors in Women with Repeated Implantation Failure: A Pilot Study. *Journal of Clinical Medicine* 2022: **11(9)**:2481; 16.

Celik O, Yurci A, Ersahin A, Gungor ND, Celik N, Ozcil MD, Dogan S, Dalkilic S, Dalkilic L, Ulug U*, et al.* Endometrial Injury Upregulates Expression of Receptivity Genes in Women with Implantation Failure. *International Journal of Environmental Research and Public Health* 2023: **20(5)**:3942.

Cernera G, Liguori R, Bruzzese D, Castaldo G, DePlacido G, Conforti A, Amato F, Alviggi C, and Comegna M. The relevance of prothrombotic genetic variants in women who experienced pregnancy loss or embryo implantation failure: A retrospective analysis of 1922 cases. *International Journal of Gynecology and Obstetrics* 2024: **165(1)**; 148-154.

Chaouat G, Menu E, Delage G, Moreau JF, Khrishnan L, Hui L, Meliani AA, Martal J, Raghupathy R, Lelaidier C*, et al.* Immuno-endocrine interactions in early pregnancy. *Human Reproduction* 1995: **10**; 55-59.

Chen CH, Lu F, Yang WJ, Yang PE, Chen WM, Kang ST, Huang YS, Kao YC, Feng CT, Chang PC*, et al.* A novel platform for discovery of differentially expressed microRNAs in patients with repeated implantation failure. *Fertil Steril* 2021: **116(1)**; 181-188.

Chen J, He A, Zhang Q, Zhao J, Fu J, Li H, and Li Y. The RNA-seq based endometrial receptivity test (rsERT) compared to pinopode: A better diagnostic tool for endometrial receptivity for patients with recurrent implantation failure in Chinese population. *Frontiers in Endocrinology* 2022: **13**:1009161.

Chen MY, Liao GD, Zhou B, Kang LN, He YM, and Li SW. Genome-Wide Profiling of Long Noncoding RNA Expression Patterns in Women With Repeated Implantation Failure by RNA Sequencing. *Reproductive sciences (Thousand Oaks, Calif.)* 2019: **26(1)**; 18‐25.

Chen P, Guo Y, Fang C, and Li T. Interaction Between Chronic Endometritis Caused Endometrial Microbiota Disorder and Endometrial Immune Environment Change in Recurrent Implantation Failure. *Frontiers in Immunology* 2021: **12**:748447.

Chen P, Jia L, Zhou Y, Guo Y, Fang C, and Li T. Interaction between endometrial microbiota and host gene regulation in recurrent implantation failure. *Journal of assisted reproduction and genetics* 2022: **39(9)**; 2169-2178.

Chen P, Li T, Guo Y, Jia L, Wang Y, and Fang C. Construction of Circulating MicroRNAs-Based Non-invasive Prediction Models of Recurrent Implantation Failure by Network Analysis. *Front Genet* 2021: **12**; 712150.

Chen PG, Li TT, Yang X, Chen LN, Guo YC, Chen PY, Liang XY, and Fang C. Association between monoaromatic hydrocarbons exposure and vaginal microbiota disorder in recurrent implantation failure. *Journal of Hazardous Materials Advances* 2023: **10**:100320; 9.

Chen PG, Yang M, Wang YF, Guo YC, Liu Y, Fang C, and Li TT. Aging endometrium in young women: molecular classification of endometrial aging-based markers in women younger than 35 years with recurrent implantation failure. *Journal of assisted reproduction and genetics* 2022: **39(9)**; 2143-2151.

Chen PP, Gao MZ, Zhao XM, Sun Y, Fang SP, and Mao L. Clinical significance of hysteroscopy after repeated implantation failure in in vitro fertilization and embryo transfer. *Journal of Shanghai Jiaotong University (Medical Science)* 2012: **32(8)**; 992-996.

Chen RZ, Wang YF, Chen PG, Fang C, and Li TT. Clinical application of the endometrial microbiota test combined with personalized treatment in patients with repeated implantation failure. *Reproductive and Developmental Medicine* 2024: **8(1)**; 18-23.

Chen W, Yang Q, Hu L, Wang M, Yang Z, Zeng X, and Sun Y. Shared diagnostic genes and potential mechanism between PCOS and recurrent implantation failure revealed by integrated transcriptomic analysis and machine learning. *Frontiers in Immunology* 2023: **14**:1175384.

Chen X, Diao L, Lian R, Qi L, Yu S, Liu S, Lin S, Xue Z, and Zeng Y. Potential impact of maternal vitamin D status on peripheral blood and endometrium cellular immunity in women with recurrent implantation failure. *Am J Reprod Immunol* 2020: **84(1)**; e13243.

Chen X, Mariee N, Jiang L, Liu Y, Wang CC, Li TC, and Laird S. Measurement of uterine natural killer cell percentage in the periimplantation endometrium from fertile women and women with recurrent reproductive failure: establishment of a reference range. *American Journal of Obstetrics and Gynecology* 2017: **217(6)**; 680.e681-680.e686.

Chen X, Qi L, Zhao C, Xue J, Chen M, Diao L, He W, Lv B, Zeng Y, and Xue Z. Decreased expression of SEMA4D in recurrent implantation failure induces reduction of trophoblast invasion and migration via the Met/PI3K/Akt pathway. *Journal of reproductive immunology* 2022: **153**:103657.

Chen X, Zhang W, Xu Y, Li W, Wang X, Shi S, and Liu X. Efficacy of the long-acting gonadotropin-releasing hormone agonist long protocol on IVF/ICSI outcomes of patients with repeated implantation failure. *International Journal of Gynecology and Obstetrics* 2024: **164(2)**; 563-570.

Cheng W, Wu YA, Wu HH, Zou QY, Meng QX, Wang FX, Li HM, Xu YL, Zhao NN, Zhou Y*, et al.* Improved pregnancy outcomes of cyclosporine A on patients with unexplained repeated implantation failure in IVF/ICSI cycles: A retrospective cohort study. *American journal of reproductive immunology* 2022: **87(4)**:e13525; 10.

Chin TH, Hsu YC, Soong YK, Lee CL, Wang HS, Huang HY, Wu HM, Yu HT, Huang SY, and Chang CL. Obstetric and perinatal outcomes of pregnancy in patients with repeated implantation failure. *Taiwan J Obstet Gynecol* 2019: **58(4)**; 487-491.

Cho SH, Chung KW, Kim JO, Jang H, Yoo JK, Choi Y, Ko JJ, Kim JH, Nishi Y, Yanase T*, et al.* Association of miR-146aC>G, miR-149C>T, miR-196a2T>C, and miR-499A>G polymorphisms with risk of recurrent implantation failure in Korean women. *Eur J Obstet Gynecol Reprod Biol* 2016: **202**; 14-19.

Cho SH, Kim JH, An HJ, Kim JO, Kim YR, Lee WS, and Kim NK. Association of methionine synthase (rs1805087), methionine synthase reductase (rs1801394), and methylenetetrahydrofolate dehydrogenase 1 (rs2236225) genetic polymorphisms with recurrent implantation failure. *Hum Fertil (Camb)* 2021: **24(3)**; 161-168.

Cho SH, Kim YM, An HJ, Kim JH, and Kim NK. miR-665-Mediated Regulation of AHCYL2 and BVES Genes in Recurrent Implantation Failure. *Genes* 2024: **15(2)**:244.

Choi Y, Kim HR, Lim EJ, Park M, Yoon JA, Kim YS, Kim EK, Shin JE, Kim JH, Kwon H*, et al.* Integrative Analyses of Uterine Transcriptome and MicroRNAome Reveal Compromised LIF-STAT3 Signaling and Progesterone Response in the Endometrium of Patients with Recurrent/Repeated Implantation Failure (RIF). *PLoS One* 2016: **11(6)**; e0157696.

Choi Y, Kim JO, Shim SH, Lee Y, Kim JH, Jeon YJ, Ko JJ, Lee WS, and Kim NK. Genetic Variation of Methylenetetrahydrofolate Reductase (MTHFR) and Thymidylate Synthase (TS) Genes Is Associated with Idiopathic Recurrent Implantation Failure. *PLoS One* 2016: **11(8)**; e0160884.

Chou PY, Wu MH, Pan HA, Hung KH, and Chang FM. Use of an oxytocin antagonist in in vitro fertilization-embryo transfer for women with repeated implantation failure: a retrospective study. *Taiwan J Obstet Gynecol* 2011: **50(2)**; 136-140.

Cicinelli E, Matteo M, Tinelli R, Lepera A, Alfonso R, Indraccolo U, Marrocchella S, Greco P, and Resta L. Prevalence of chronic endometritis in repeated unexplained implantation failure and the IVF success rate after antibiotic therapy. *Hum Reprod* 2015: **30(2)**; 323-330.

Cimadomo D, Capalbo A, Dovere L, Tacconi L, Soscia D, Giancani A, Scepi E, Maggiulli R, Vaiarelli A, Rienzi L*, et al.* Leave the past behind: women's reproductive history shows no association with blastocysts' euploidy and limited association with live birth rates after euploid embryo transfers. *Hum Reprod* 2021: **36(4)**; 929-940.

Cohen AM, Ye XY, Colgan TJ, Greenblatt EM, and Chan C. Comparing endometrial receptivity array to histologic dating of the endometrium in women with a history of implantation failure. *Syst Biol Reprod Med* 2020: **66(6)**; 347-354.

Cohen SB, Bookstein Peretz S, Hakim S, Orvieto R, and Burke YZ. Hysteroscopicmetroplasty as a treatment option for women with congenital dysmorphic uterus suffering from impaired reproductive performance. *Human fertility* 2023: **26(5)**; 1087-1092.

Coksuer H, Akdemir Y, and Barut MU. Improved in vitro fertilization success and pregnancy outcome with autologous platelet-rich plasma treatment in unexplained infertility patients that had repeated implantation failure history. *Gynecological endocrinology* 2019: **35(9)**; 815-818.

Comins-Boo A, Cristobal I, Fernandez-Arquero M, de Frias ER, Urrutia MC, Suarez LP, Escorial PG, Herraiz MA, and Sanchez-Ramon S. Functional NK surrogate biomarkers for inflammatory recurrent pregnancy loss and recurrent implantation failure. *American journal of reproductive immunology* 2021: **86(2)**:e13426.

Comins-Boo A, Valdeolivas L, Perez-Pla F, Cristobal I, Subhi-Issa N, Dominguez-Soto A, Pilar-Suarez L, Gasca-Escorial P, Calvo-Urrutia M, Fernandez-Arquero M*, et al.* Immunophenotyping of peripheral blood monocytes could help identify a baseline pro-inflammatory profile in women with recurrent reproductive failure. *Journal of reproductive immunology* 2022: **154**:103735.

Cooper S, Laird SM, Mariee N, Li TC, and Metwally M. The effect of prednisolone on endometrial uterine NK cell concentrations and pregnancy outcome in women with reproductive failure. A retrospective cohort study. *J Reprod Immunol* 2019: **131**; 1-6.

Coughlan C, Clarke H, Cutting R, Saxton J, Waite S, Ledger W, Li T, and Pacey AA. Sperm DNA fragmentation, recurrent implantation failure and recurrent miscarriage. *Asian J Androl* 2015: **17(4)**; 681-685.

Coughlan C, Sinagra M, Ledger W, Li TC, and Laird S. Endometrial integrin expression in women with recurrent implantation failure after in vitro fertilization and its relationship to pregnancy outcome. *Fertil Steril* 2013: **100(3)**; 825-830.

Coughlan C, Walters S, Ledger W, and Li TC. A comparison of psychological stress among women with and without reproductive failure. *Int J Gynaecol Obstet* 2014: **124(2)**; 143-147.

Coughlan C, Yuan X, Demirol A, Ledger W, and Li TC. Factors affecting the outcome of "endometrial scratch" in women with recurrent implantation failure. *J Reprod Med* 2014: **59(1-2)**; 39-43.

Coughlan C, Yuan X, Nafee T, Yan J, Mariee N, and Li T. The clinical characteristics of women with recurrent implantation failure. *Journal of Obstetrics and Gynaecology* 2013: **33(5)**; 494-498.

Coulam CB, Bilal M, Garcia MDS, Katukurundage D, Elazzamy H, Fernandez EF, Kwak-Kim J, Beaman K, and Dambaeva SV. Prevalence of HHV-6 in endometrium from women with recurrent implantation failure. *American journal of reproductive immunology* 2018: **80(1)**:e12862; 6.

Coulam CB, Jeyendran RS, Fishel LA, and Roussev R. Multiple thrombophilic gene mutations are risk factors for implantation failure. *Reprod Biomed Online* 2006: **12(3)**; 322-327.

Coulam CB, Jeyendran RS, and Roussev R. Association of progesterone receptor polymorphisms with recurrent implantation failure after in vitro fertilization and embryo transfer. *J Assist Reprod Genet* 2008: **25(4)**; 119-122.

Coutanceau B, Dos Santos E, Swierkowski Blanchard N, Sanchez Louboutin A, Boitrelle F, Margueritte F, Vialard F, Serazin V, and Fathallah K. Should the Treatment of Patients with Repeated Embryo Implantation Failure Be Adapted as a Function of the Endometrial Cytokine Profile? A Single-Center Experience. *Biomedicines* 2023: **11(3)**:817.

Cozzolino M, Diaz-Gimeno P, Pellicer A, and Garrido N. Evaluation of the endometrial receptivity assay and the preimplantation genetic test for aneuploidy in overcoming recurrent implantation failure. *J Assist Reprod Genet* 2020: **37(12)**; 2989-2997.

Curfs MHJM, Cohlen BJ, Slappendel EJ, Schoot DC, Derhaag JG, Van Golde RJT, Van Der Heijden GW, Baart EB, Smeenk JMJ, Ritfeld VEEG*, et al.* A multicentre double-blinded randomized controlled trial on the efficacy of laser-assisted hatching in patients with repeated implantation failure undergoing IVF or ICSI. *Human Reproduction* 2023: **38(10)**; 1952-1960.

Darabi N, Mashayekhy M, BorjianBoroujeni P, Mohtasebi P, RokhsatTalab Z, and Zamanian MR. APOE-E4 allele as a potential marker for implantation failure: A comparison between fertile women, ART success and RIF patients. *International Journal of Gynecology and Obstetrics.* 2024: **167(1)**; 169-176.

Dashti S, Mirzaei M, Eftekhar M, and Mangoli E. Is there any difference between the obstetric and perinatal complications of pregnancy in patients with and without repeated implantation failure in fresh and frozen-thawed embryo transfer cycles? *Middle east fertility society journal* 2022: **27(1)**:6; 7.

Davar R, Dashti S, and Omidi M. Endometrial preparation using gonadotropin-releasing hormone agonist prior to frozen-thawed embryo transfer in women with repeated implantation failure: AN RCT. *International journal of reproductive biomedicine* 2020: **18(5)**; 319‐326.

Davari-Tanha F, Shahrokh Tehraninejad E, Ghazi M, and Shahraki Z. The role of G-CSF in recurrent implantation failure: A randomized double blind placebo control trial. *Int J Reprod Biomed* 2016: **14(12)**; 737-742.

De Sutter P, Stadhouders R, Dutré M, Gerris J, and Dhont M. Prevalence of chromosomal abnormalities and timing of karyotype analysis in patients with recurrent implantation failure (RIF) following assisted reproduction. *Facts Views Vis Obgyn* 2012: **4(1)**; 59-65.

Debrock S, Spiessens C, Peeraer K, De Loecker P, Willemen D, and D'Hooghe TM. Higher implantation rate using modified quarter laser-assisted zona thinning in repeated implantation failure. *Gynecol Obstet Invest* 2009: **67(2)**; 127-133.

Decleer W, Osmanagaoglu K, and Devroey P. The role of oxytocin antagonists in repeated implantation failure. *Facts Views and Vision in Obgyn* 2012: **4(4)**; 227-229.

del Carmen Nogales M, Cruz M, de Frutos S, Martínez EM, Gaytán M, Ariza M, Bronet F, and Garcia-Velasco JA. Association between clinical and IVF laboratory parameters and miscarriage after single euploid embryo transfers. *Reproductive Biology and Endocrinology* 2021: **19(1)**:186.

Demirdag E, Guler I, Akdulum MFC, Sahin E, Erdem O, Erdem A, and Erdem M. Subsequent IVF outcomes following antibiotic therapy for chronic endometritis in patients with recurrent implantation failure. *Journal of Obstetrics and Gynaecology Research* 2021: **47(12)**; 4350-4356.

Desai N, AbdelHafez F, Bedaiwy MA, and Goldfarb J. Live births in poor prognosis IVF patients using a novel non-contact human endometrial co-cultural system. *Reproductive biomedicine online* 2008: **16(6)**; 869-874.

Dhaenens L, Lierman S, De Clerck L, Govaert E, Deforce D, Tilleman K, and De Sutter P. Endometrial stromal cell proteome mapping in repeated implantation failure and recurrent pregnancy loss cases and fertile women. *Reprod Biomed Online* 2019: **38(3)**; 442-454.

Diao LH, Li GG, Zhu YC, Tu WW, Huang CY, Lian RC, Chen X, Li YY, Zhang T, Huang Y*, et al.* Human chorionic gonadotropin potentially affects pregnancy outcome in women with recurrent implantation failure by regulating the homing preference of regulatory T cells. *Am J Reprod Immunol* 2017: **77(3)**:e12618.

Diaz-Martinez MC, Bernabeu A, Lledo B, Carratala-munuera C, Quesada JA, Lozano FM, Ruiz V, Morales R, Llacer J, Ten J*, et al.* Impact of the vaginal and endometrial microbiome pattern on assisted reproduction outcomes. *Journal of Clinical Medicine* 2021: **10(18)**:4063.

Dieamant F, Vagnini LD, Petersen CG, Mauri AL, Renzi A, Petersen B, Mattila MC, Nicoletti A, Oliveira JBA, Baruffi R*, et al.* New therapeutic protocol for improvement of endometrial receptivity (PRIMER) for patients with recurrent implantation failure (RIF) - A pilot study. *JBRA assisted reproduction* 2019: **23(3)**; 250‐254.

Ding JL, Diao LH, Yin TL, Huang CY, Yin B, Chen C, Zhang Y, Li J, Cheng YX, Zeng Y*, et al.* Aberrant expressions of endometrial Id3 and CTLA-4 are associated with unexplained repeated implantation failure and recurrent miscarriage. *Am J Reprod Immunol* 2017: **78(2)**:e12632.

Dolanbay EG, Yardimoglu M, Yalcinkaya E, Yazir Y, Aksoy A, Karaoz E, and Caliskan E. Expression of trophinin and dipeptidyl peptidase IV in endometrial co-culture in the presence of an embryo: A comparative immunocytochemical study. *Mol Med Rep* 2016: **13(5)**; 3961-3968.

Dong J, Fu J, Yan Z, Li L, Qiu Y, Zeng Y, Liu RY, Chen BB, Shi R, Diao FY*, et al.* Novel biallelic mutations in PADI6 in patients with early embryonic arrest. *Journal of Human Genetics* 2022: **67(5)**; 285-293.

Dong J, Zhang H, Mao XY, Zhu JH, Li D, Fu J, Hu JJ, Wu L, Chen BB, Sun YM*, et al.* Novel biallelic mutations in MEI1: expanding the phenotypic spectrum to human embryonic arrest and recurrent implantation failure. *Human Reproduction* 2021: **36(8)**; 2371-2381.

Dong X, Zhou M, Li X, Huang H, and Sun Y. Gene profiling reveals the role of inflammation, abnormal uterine muscle contraction and vascularity in recurrent implantation failure. *Frontiers in Genetics* 2023: **14**:1108805.

Dong Y, Liu D, Zou Y, Wan C, Chen C, Dong M, Huang Y, Huang C, Weng H, Zhu X*, et al.* Evaluate the risk in conventional IVF frozen human blastocysts undergoing PGT using a new quantification method for parental contamination testing (qPCT). *medRxiv* 2022:2022.01.23.22269520.

Donoghue JF, Paiva P, Teh WT, Cann LM, Nowell C, Rees H, Bittinger S, Obers V, Bulmer JN, Stern C*, et al.* Endometrial uNK cell counts do not predict successful implantation in an IVF population. *Hum Reprod* 2019: **34(12)**; 2456-2466.

Dons'koi BV, Osypchuk DV, Chernyshov VP, and Khazhylenko KG. Expression of natural cytotoxicity receptor NKp46 on peripheral blood natural killer cells in women with a history of recurrent implantation failures. *J Obstet Gynaecol Res* 2021: **47(3)**; 1009-1015.

Dos Santos E, Serazin V, Morvan C, Torre A, Wainer R, de Mazancourt P, and Dieudonné MN. Adiponectin and leptin systems in human endometrium during window of implantation. *Fertil Steril* 2012: **97(3)**; 771-778.e771.

Drissennek L, Baron C, Brouillet S, Entezami F, Hamamah S, and Haouzi D. Endometrial miRNome profile according to the receptivity status and implantation failure. *Human fertility* 2020: **25(2)**; 356-368.

Du Y, Guan Y, Li N, Shi C, Zhang Y, Ren B, Liu J, and Lou H. Is it necessary for young patients with recurrent implantation failure to undergo preimplantation genetic testing for aneuploidy? *Frontiers in Endocrinology* 2023: **14**:1020055.

Duan Y, Liu Y, Xu Y, and Zhou C. Bioinformatics Analysis Identifies Key Genes in Recurrent Implantation Failure Based on Immune Infiltration. *Reproductive Sciences* 2023: **30(3)**; 952-965.

Edimiris P, Doehmen C, Baston-Buest DM, Kruessel JS, and Bielfeld AP. One center experience with a personalized frozen-thawed embryo transfer in patients with recurrent implantation failure. *Journal of assisted reproduction and genetics* 2023: **40(7)**; 1639-1647.

Eftekhar M, Neghab N, and Khani P. Effectiveness of Autologous Platelet-Rich Plasma Therapy in Women with Repeated Implantation Failure: A Randomized Clinical Trial. *International journal of fertility and sterility* 2024: **18(2)**; 162-166.

Eker C, Basdas R, Balci BK, Bastu E, and Gunel T. The genomic analysis of endometrial mitochondrial DNA copy number variation on recurrent implantation failure. *J Gynecol Obstet Hum Reprod* 2021: **50(2)**:101945.

El Khattabi L, Dupont C, Sermondade N, Hugues JN, Poncelet C, Porcher R, Cedrin-Durnerin I, Lévy R, and Sifer C. Is intracytoplasmic morphologically selected sperm injection effective in patients with infertility related to teratozoospermia or repeated implantation failure? *Fertil Steril* 2013: **100(1)**; 62-68.

Elmahdy M, Elfourti I, and Maghrab H. Office hysteroscopy in cases of recurrent implantation failure; Do or not to do. *Clinical and Experimental Obstetrics and Gynecology* 2020: **47(5)**; 723-728.

Elmi M, Ghandil P, Hemadi M, Birgani MT, and Saberi A. Association of rs1570360 and rs2010963 in VEGF and rs2279744 in the MDM2 gene with Recurrent Implantation Failure in Iranian Women. *Jornal brasileiro de reproducao assistida* 2023: **27(3)**; 342-347.

Enatsu Y, Enatsu N, Kishi K, Otsuki J, Iwasaki T, Okamoto E, Kokeguchi S, and Shiotani M. Clinical outcome of intrauterine infusion of platelet-rich plasma in patients with recurrent implantation failure. *Reproductive Medicine and Biology* 2021: **21(1)**:e12417.

Engin-Ustun Y, Ozgu-Erdinc AS, Caglayan EK, Gulerman C, Sarikaya E, Aktulay A, Demirtas C, Erkaya S, and Yilmaz N. Sirtuin 1 levels in recurrent implantation failure. *Revista Brasileira de Ginecologia e Obstetricia* 2017: **39(10)**; 541-544.

Engin-Üstün Y, Özgü-Erdinç AS, Yilmaz N, Erkaya S, Aktulay A, Akgün N, and Demirtaş C. Human chitinase-3-like protein: A pathogenic role in recurrent implantation failure? *Medeniyet medical journal* 2018: **33(2)**; 100-104.

Ercelen N, Turtar E, Gultomruk M, Comert H, Coskun H, Mercan R, and Nuhoglu A. Successful preimplantation genetic aneuploidy screening in Turkish patients. *Genet Mol Res* 2011: **10(4)**; 4093-4103.

Ersahin A, Acet M, Acet T, and Yavuz Y. Disturbed endometrial NF-κB expression in women with recurrent implantation failure. *Eur Rev Med Pharmacol Sci* 2016: **20(24)**; 5037-5040.

Ersahin S, Ersahin A, Gungor ND, Gungor K, Yalçin D, Ersahin C, and Celik N. High serum AMH inhibits pathological growth of the low biomass endometrial microbiome. *European review for medical and pharmacological sciences* 2022: **26(20)**; 7600-7604.

Ersahin SS and Ersahin A. Endometrial injury concurrent with hysteroscopy increases the expression of Leukaemia inhibitory factor: a preliminary study. *Reproductive Biology and Endocrinology* 2022: **20**:11.

Ershadi S, Noori N, Dashipoor A, Ghasemi M, and Shamsa N. Evaluation of the effect of intrauterine injection of platelet-rich plasma on the pregnancy rate of patients with a history of implantation failure in the in vitro fertilization cycle. *Journal of Family Medicine and Primary Care* 2022: **11(5)**; 2162-2166.

Esmaeilzadeh S, Mohammadi A, Mahdinejad N, Ghofrani F, and Ghasemzadeh-Hasankolaei M. Receptivity markers in endometrial mesenchymal stem cells of recurrent implantation failure and non-recurrent implantation failure women: A pilot study. *J Obstet Gynaecol Res* 2020: **46(8)**; 1393-1402.

Fan L, Han HJ, Guan J, Zhang X, Cui Q, Shen H, and Shi C. Aberrantly expressed long noncoding RNAs in recurrent implantation failure: A microarray related study. *Systems Biology in Reproductive Medicine* 2017: **63(4)**; 269-278.

Fan L, Zhang F, Yao C, Nong L, Li J, and Huang W. Unraveling the H19/GAS1 axis in recurrent implantation failure: A potential biomarker for diagnosis and insight into immune microenvironment alteration. *PLoS One* 2024: **19(7)**; e0306244.

Fan X, Zhao Q, Li Y, Chen Z, Liao J, Chen H, Meng F, Lu GX, Lin G, and Gong F. Immune profiling and RNA-seq uncover the cause of partial unexplained recurrent implantation failure. *International immunopharmacology* 2023: **121**:110513.

Fan Y, Lee RWK, Ng XW, Gargett CE, and Chan JKY. Subtle changes in perivascular endometrial mesenchymal stem cells after local endometrial injury in recurrent implantation failure. *Scientific reports* 2023: **13(1)**; 225.

Fan Y, Shi C, Huang N, Fang F, Tian L, and Wang J. Recurrent Implantation Failure: Bioinformatic Discovery of Biomarkers and Identification of Metabolic Subtypes. *International Journal of Molecular Sciences* 2023: **24(17)**:13488.

Fang Q, Qiao Z, Luo L, Bai S, Chen M, Zhang X, Zong L, Tong XH, and Wu LM. Predictive models of recurrent implantation failure in patients receiving ART treatment based on clinical features and routine laboratory data. *Reproductive Biology and Endocrinology* 2024: **22(1)**:32.

Farhi J, Ben-Haroush A, Dresler H, Pinkas H, Sapir O, and Fisch B. Male factor infertility, low fertilisation rate following ICSI and low number of high-quality embryos are associated with high order recurrent implantation failure in young IVF patients. *Acta Obstet Gynecol Scand* 2008: **87(1)**; 76-80.

Farhi J, Weissman A, Nahum H, and Levran D. Zygote intrafallopian transfer in patients with tubal factor infertility after repeated failure of implantation with in vitro fertilization-embryo transfer. *Fertil Steril* 2000: **74(2)**; 390-393.

Farifteh F, Fazeli E, Hosseini SZ, Arefi SS, Moini A, Taheripanah R, Rouhollah F, Salehi M, Hosseini A, and Benkhalifa M. Intrauterine administration of autologous peripheral blood mononuclear cells regulates the endometrium estrogen and progesterone receptor expression: An RCT. *International journal of reproductive biomedicine* 2023: **21(4)**; 343-354.

Farzadi L, Fakour A, Ghasemzadeh A, Hamdi K, Fard SA, Nouri M, Niknafs B, and Shahnazi V. The effect of local endometrial injury and GnRH agonist on pregnancy rate in patients with recurrent implantation failure. *International Journal of Women's Health and Reproduction Sciences* 2016: **4(1)**; 34-37.

Feng C, Shen JM, Lv PP, Jin M, Wang LQ, Rao JP, and Feng L. Construction of implantation failure related lncRNA-mRNA network and identification of lncRNA biomarkers for predicting endometrial receptivity. *Int J Biol Sci* 2018: **14(10)**; 1361-1377.

Feng X, Meng X, Guo S, Li K, Wang L, and Ai J. Identification of key genes and immune cell infiltration in recurrent implantation failure: A study based on integrated analysis of multiple microarray studies. *American journal of reproductive immunology* 2022: **88(4)**:e13607.

Ferro J, Labarta E, Sanz C, Montoya P, and Remohi J. Reproductive outcomes after hysteroscopic metroplasty for women with dysmorphic uterus and recurrent implantation failure. *Facts Views Vis Obgyn* 2018: **10(2)**; 63-68.

Firouzabadi RD, Ghasemi N, Rozbahani MA, and Tabibnejad N. Association of p53 polymorphism with ICSI/IVF failure and recurrent pregnancy loss. *Aust N Z J Obstet Gynaecol* 2009: **49(2)**; 216-219.

Fodina V, Dudorova A, and Erenpreiss J. Evaluation of embryo aneuploidy (PGT-A) and endometrial receptivity (ERA) testing in patients with recurrent implantation failure in ICSI cycles. *Gynecol Endocrinol* 2021: **37(sup1)**; 17-20.

Foulk RA, Zdravkovic T, Genbacev O, and Prakobphol A. Expression of L-selectin ligand MECA-79 as a predictive marker of human uterine receptivity. *J Assist Reprod Genet* 2007: **24(7)**; 316-321.

Foyle KL, Sharkey DJ, Moldenhauer LM, Green ES, Wilson JJ, Roccisano CJ, Hull ML, Tremellen KP, and Robertson SA. Effect of Intralipid infusion on peripheral blood T cells and plasma cytokines in women undergoing assisted reproduction treatment. *Clinical and Translational Immunology* 2021: **10(8)**:e1328.

Fragouli E, Katz-Jaffe M, Alfarawati S, Stevens J, Colls P, Goodall NN, Tormasi S, Gutierrez-Mateo C, Prates R, Schoolcraft WB*, et al.* Comprehensive chromosome screening of polar bodies and blastocysts from couples experiencing repeated implantation failure. *Fertil Steril* 2010: **94(3)**; 875-887.

Freitag N, Pour SJ, Fehm TN, Toth B, Markert UR, Weber M, Togawa R, Kruessel JS, Baston-Buest DM, and Bielfeld AP. Are uterine natural killer and plasma cells in infertility patients associated with endometriosis, repeated implantation failure, or recurrent pregnancy loss? *Arch Gynecol Obstet* 2020: **302(6)**; 1487-1494.

Fu H, Tan W, Chen Z, Ye Z, Duan Y, Huang J, Qi H, and Liu X. TOP2A deficit-induced abnormal decidualization leads to recurrent implantation failure via the NF-κB signaling pathway. *Reprod Biol Endocrinol* 2022: **20(1)**; 142.

Fu YX, Yang HM, OuYang XE, Hu R, Hu T, and Wang FM. Assessment of Anti-Mullerian Hormone and Anti-Mullerian Hormone Type II Receptor Variants in Women with Repeated Implantation Failures. *Reprod Sci* 2021: **28(2)**; 406-415.

Fujii S and Oguchi T. The number of previous implantation failures is a critical determinant of intrauterine autologous platelet-rich plasma infusion success in women with recurrent implantation failure. *Reproductive Medicine and Biology* 2024: **23(1)**:e12565.

Gan D, Liu LY, Zhong Y, Wu YN, Lai YY, Wei W, Guo XL, Tian R, Yu SY, Liang FR*, et al.* Acupuncture benefits to women with recurrent implantation failure: A propensity score-matched cohort study. *Heliyon* 2023: **9(7)**; e18193.

Ganeva R, Parvanov D, Vidolova N, Ruseva M, Handzhiyska M, Arsov K, Decheva I, Metodiev D, Moskova-Doumanova V, and Stamenov G. Endometrial immune cell ratios and implantation success in patients with recurrent implantation failure. *Journal of reproductive immunology* 2023: **156**:103816.

Gao J, Guo H, Zhu L, Yang B, Zhang J, Xu K, and Hao C. Analysis of blocking antibodies and lymphocyte subsets in patients with recurrent reproductive failure. *Human fertility* 2022: **25(3)**; 569-574.

Gao J, Yuan Y, Li J, Tian T, Lian Y, Liu P, Li R, Qiao J, Long X, and Wang H. Sequential embryo transfer versus double cleavage-stage embryo or double blastocyst transfer in patients with recurrent implantation failure with frozen-thawed embryo transfer cycles: a cohort study. *Frontiers in Endocrinology* 2023: **14**:1238251.

Gao M, Sun Y, Xie H, Fang S, and Zhao X. Hysteroscopy prior to repeat embryo transfer may improve pregnancy outcomes for asymptomatic women with repeated implantation failure. *J Obstet Gynaecol Res* 2015: **41(10)**; 1569-1576.

Gao MZ, Xin MZ, Zhao XM, Zheng Z, Hong Y, Sun Y, and Zhang HQ. Endometrial Collagen Fibril Hyperplasia is Associated with Implantation Failure in Women Undergoing IVF-ET. *Journal of Reproduction and Contraception* 2009: **20(3)**; 131-144.

Gao Q, Ma C, Meng S, Wang G, Xing Q, Xu Y, He X, Wang T, and Cao Y. Exploration of molecular features of PCOS with different androgen levels and immune-related prognostic biomarkers associated with implantation failure. *Frontiers in Endocrinology* 2022: **13**:946504.

Garcia MDS, Sung N, Mullenix TM, Dambaeva S, Beaman K, Gilman-Sachs A, and Kwak-Kim J. Plasminogen Activator Inhibitor-1 4G/5G Polymorphism is Associated with Reproductive Failure: Metabolic, Hormonal, and Immune Profiles. *American journal of reproductive immunology* 2016: **76(1)**; 70-81.

Gat I, Levron J, Yerushalmi G, Dor J, Brengauz M, and Orvieto R. Should zygote intrafallopian transfer be offered to all patients with unexplained repeated in-vitro fertilization cycle failures? *J Ovarian Res* 2014: **7**; 7.

Germeyer A, von Wolff M, Jauckus J, Strowitzki T, Sharma T, and Grazul-Bilska AT. Changes in cell proliferation, but not in vascularisation are characteristic for human endometrium in different reproductive failures - a pilot study. *Reproductive Biology and Endocrinology* 2010: **8**:67.

Ghaebi M, Abdolmohammadi-Vahid S, Ahmadi M, Eghbal-Fard S, Dolati S, Nouri M, Talebi M, Hamdi K, Marofi F, Aghebati-Maleki L*, et al.* T cell Subsets in Peripheral Blood of Women with Recurrent Implantation Failure. *J Reprod Immunol* 2019: **131**; 21-29.

Ghaebi NK, Mahmoudiniya M, Najafi MN, Zohdi E, and Attaran M. Comparison of letrozole with gonadotropin-releasing hormone agonist in frozen embryo transfer after recurrent implantation failure: an RCT. *International journal of reproductive biomedicine* 2020: **18(2)**; 105‐112.

Ghasemnejad-Berenji H, Ghaffari Novin M, Hajshafiha M, Nazarian H, Hashemi SM, Ilkhanizadeh B, Ghasemnejad T, Sadeghpour S, and Ghasemnejad-Berenji M. Immunomodulatory effects of hydroxychloroquine on Th1/Th2 balance in women with repeated implantation failure. *Biomed Pharmacother* 2018: **107**; 1277-1285.

Ghobara TS, Cahill DJ, Ford WC, Collyer HM, Wilson PE, Al-Nuaim L, and Jenkins JM. Effects of assisted hatching method and age on implantation rates of IVF and ICSI. *Reprod Biomed Online* 2006: **13(2)**; 261-267.

Ghorbani S, Abedi P, Hekmat K, Ghanbari S, and Dibavand N. Recurrent implantation failure and sexual function in infertile Iranian women: a comparative cross sectional study. *Reproductive health* 2022: **19(1)**:103; 7.

Gil Laborda R, de Frias ER, Subhi-Issa N, de Albornoz EC, Melia E, Ordenes M, Verdu V, Vidal J, Suarez E, Santillan I*, et al.* Centromeric AA motif in KIR as an optimal surrogate marker for precision definition of alloimmune reproductive failure. *Scientific reports* 2024: **14(1)**; 3354.

Giulini S, Grisendi V, Sighinolfi G, Di Vinci P, Tagliasacchi D, Botticelli L, La Marca A, and Facchinetti F. Chronic endometritis in recurrent implantation failure: Use of prednisone and IVF outcome. *Journal of reproductive immunology* 2022: **153**:103673; 5.

Gong QQ, Zhu YJ, Pang NN, Ai HQ, Gong XY, La XL, and Ding JB. Increased levels of CCR7(lo) PD-1(hi) CXCR5(+) CD4(+) T cells, and associated factors Bcl-6, CXCR5, IL-21 and IL-6 contribute to repeated implantation failure. *Experimental and Therapeutic Medicine* 2017: **14(6)**; 5931-5941.

Goodman C, Jeyendran RS, and Coulam CB. Vascular endothelial growth factor gene polymorphism and implantation failure. *Reprod Biomed Online* 2008: **16(5)**; 720-723.

Goodman C, Jeyendran RS, and Coulam CB. P53 tumor suppressor factor, plasminogen activator inhibitor, and vascular endothelial growth factor gene polymorphisms and recurrent implantation failure. *Fertil Steril* 2009: **92(2)**; 494-498.

Govahi A, Nasr-Esfahani MH, Amjadi F, Mahdevar M, Mehdizadeh R, and Mehdizadeh M. Cutting-edge techniques provide insights regarding repeated implantation failure patients. *Reproductive biomedicine online* 2023: **46(4)**; 687-696.

Greco E, Bono S, Ruberti A, Lobascio AM, Greco P, Biricik A, Spizzichino L, Greco A, Tesarik J, Minasi MG*, et al.* Comparative genomic hybridization selection of blastocysts for repeated implantation failure treatment: a pilot study. *Biomed Res Int* 2014: **2014**:457913.

Gu RH, Fu J, Ge ND, Li ZC, Huang B, Xu Y, Zou YY, Li L, Sun YJ, and Sun XX. Preimplantation genetic testing for aneuploidy improves clinical outcomes in patients with repeated implantation failure. *Reproductive and Developmental Medicine* 2023: **7(1)**; 12-19.

Guenther V, Alkatout I, Meyerholz L, Maass N, Goerg S, von Otte S, and Ziemann M. Live Birth Rates after Active Immunization with Partner Lymphocytes. *Biomedicines* 2021: **9(10)**:1350; 13.

Guerif F, Bidault R, Gasnier O, Couet ML, Gervereau O, Lansac J, and Royere D. Efficacy of blastocyst transfer after implantation failure. *Reproductive biomedicine online* 2004: **9(6)**; 630-636.

Gunday OK, Aldemir O, Ozelci R, Dilbaz S, Baser E, and Tekin OM. Is Red Blood Cell Distribution Width (RDW) a Negative Predictor of Repeated Implantation Failure? *Clinical and Experimental Obstetrics and Gynecology* 2023: **50(10)**:214.

Guo F, Si CC, Zhou MJ, Wang JW, Zhang D, Leung PCK, Xu BF, and Zhang AJ. Decreased PECAM1-mediated TGF-beta 1 expression in the mid-secretory endometrium in women with recurrent implantation failure. *Human Reproduction* 2018: **33(5)**; 832-843.

Guo L, Guo AL, Yang F, Li L, Yan JH, Deng XH, Dai CF, and Li Y. Alterations of Cytokine Profiles in Patients With Recurrent Implantation Failure. *Frontiers in Endocrinology* 2022: **13**:949123; 9.

Guo L, Wang X, Wang Y, Sun F, Song Y, Chen C, Yan J, and Li Y. Impact of thyroid autoimmunity on pregnancy outcomes in euthyroid patients with recurrent implantation failure. *Reproductive biomedicine online* 2023: **47(3)**:103229.

Guo Q, Liu P, Zhou W, Xia M, Li J, Lu J, Ma JL, Chen ZJ, and Yan J. Growth hormone supplementation ameliorates blastocyst euploidy rates and improves pregnancy outcomes in women undergoing preimplantation genetic testing for aneuploidy cycles. *Frontiers in Endocrinology* 2023: **14**:1117706.

Guo Z, Shi P, and Hao C. Effect of GnRHa on Th17/Treg cells in peripheral blood of patients with unexplained repeated implantation failure. *Arch Gynecol Obstet* 2018: **298(6)**; 1211-1218.

Gürgan T, Kalem Z, Kalem MN, Ruso H, Benkhalifa M, and Makrigiannakis A. Systematic and standardized hysteroscopic endometrial injury for treatment of recurrent implantation failure. *Reproductive biomedicine online* 2019: **39(3)**; 477‐483.

Habibi B, Novin MG, Salehpour S, Novin MG, Yeganeh SM, and Nazarian H. Expression analysis of genes and MicroRNAs involved in recurrent implantation failure: New noninvasive biomarkers of implantation. *Biomedical and Biotechnology Research Journal* 2022: **6(1)**; 145-155.

Hamdi K, Danaii S, Farzadi L, Abdollahi S, Chalabizadeh A, and Abdollahi Sabet S. The Role of Heparin in Embryo Implantation in Women with Recurrent Implantation Failure in the Cycles of Assisted Reproductive Techniques (Without History of Thrombophilia). *J Family Reprod Health* 2015: **9(2)**; 59-64.

Han M, Cao Y, Zhou WJ, Zhou MJ, Zhou XW, Zhang D, Xu BF, and Zhang AJ. Increased expression of HMGB1 in the implantation phase endometrium is related to recurrent implantation failure. *Molecular Biology Reports* 2022: **49(3)**; 1701-1710.

Han S, Liu S, Jin NQ, Lv YS, Yang M, Ma S, Fu Y, Zhao SK, and Liu MH. Mitochondrial energy metabolism is downregulated in repeated implantation failure patients related to alteration of PGC-1alpha acetylation level. *Molecular Reproduction and Development* 2023: **90(6)**; 397-405.

Han S, Shu M, Song C, Chen F, Wang L, Yao S, Zhai X, Xiao X, Wang W, and Shang W. Effect of accurate assessment for uterine cavity environment on outcome of repeated transplantation failures. *Journal of Army Medical University* 2022: **44(6)**; 582-588.

Haouzi D, Entezami F, Torre A, Innocenti C, Antoine Y, Mauries C, Vincens C, Bringer-Deutsch S, Gala A, Ferrieres HA*, et al.* Customized Frozen Embryo Transfer after Identification of the Receptivity Window with a Transcriptomic Approach Improves the Implantation and Live Birth Rates in Patients with Repeated Implantation Failure. *Reproductive Sciences* 2021: **28(1)**; 69-78.

Hapangama DK, Turner MA, Drury JA, Martin-Ruiz C, Von Zglinicki T, Farquharson RG, and Quenby S. Endometrial telomerase shows specific expression patterns in different types of reproductive failure. *Reprod Biomed Online* 2008: **17(3)**; 416-424.

Harby IKA, Ahmed OI, Zaki WK, Faris MA, and El-Deen NNS. Correlation of TNF-α, IFN-γ and TGF-β Expression with In-vitro Fertilization Success Rates in Women with Recurrent Implantation Failure Undergoing Treatment with Intra-lipid Infusion. *Journal of Pure and Applied Microbiology* 2022: **16(2)**; 1307-1317.

Harrity C, Bereir MM, Walsh DJ, and Marron KD. Moving from peripheral blood to local uterine immunophenotype analysis in patients with poor reproductive history: pilot study of a novel technique. *Ir J Med Sci* 2019: **188(3)**; 893-901.

Hashimoto T, Koizumi M, Doshida M, Toya M, Sagara E, Oka N, Nakajo Y, Aono N, Igarashi H, and Kyono K. Efficacy of the endometrial receptivity array for repeated implantation failure in Japan: A retrospective, two-centers study. *Reprod Med Biol* 2017: **16(3)**; 290-296.

He A, Zou Y, Wan C, Zhao J, Zhang Q, Yao Z, Tian F, Wu H, Huang X, Fu J*, et al.* The role of transcriptomic biomarkers of endometrial receptivity in personalized embryo transfer for patients with repeated implantation failure. *Journal of translational medicine* 2021: **19(1)**; 176.

Heilmann L, Schorsch M, and Hahn T. SHORT COMMUNICATION: CD3− CD56+ CD16+ Natural Killer Cells and Improvement of Pregnancy Outcome in IVF/ICSI Failure After Additional IVIG-Treatment. *American journal of reproductive immunology* 2010: **63(3)**; 263-265.

Hirachan A and Bakshi R. Enhancing Embryo Implantation Success: The Role of Alpha Thymosin in Modulating Immune Response in Recurrent Implantation Failure. *Research Journal of Medical Sciences* 2024: **18(6)**; 306-310.

Hisano M, Nakagawa K, Kwak-Kim J, Sugiyama R, Sago H, and Yamaguchi K. Changes in the T-helper 1 and 2 cell populations during pregnancy in tacrolimus-treated women with repeated implantation failure and recurrent pregnancy loss. *Human fertility* 2021: **25(5)**; 975-982.

Ho YK, Chen HH, Huang CC, Lee CI, Lin PY, Lee MS, and Lee TH. Peripheral CD56+CD16+ NK Cell Populations in the Early Follicular Phase Are Associated With Successful Clinical Outcomes of Intravenous Immunoglobulin Treatment in Women With Repeated Implantation Failure. *Frontiers in Endocrinology* 2020: **10**:937.

Hosseini MA, Ebrahimi N, Mahdavi A, Aleyasin A, Safdarian L, Fallahi P, and Esfahani F. Hysteroscopy in patients with repeated implantation failure improves the outcome of assisted reproductive technology in fresh and frozen cycles. *J Obstet Gynaecol Res* 2014: **40(5)**; 1324-1330.

Hosseinirad H, Novin MG, Hosseini S, Nazarian H, Amidi F, Paktinat S, Azizi E, and Mofarahe ZS. Effect of 1,25(OH)2-vitamin D3 on expression and phosphorylation of progesterone receptor in cultured endometrial stromal cells of patients with repeated implantation failure. *Acta Histochem* 2020: **122(2)**; 151489.

Hosseinirad H, Novin MG, Hosseini S, Nazarian H, Safaei Z, Hashemi T, Paktinat S, and Mofarahe ZS. Evaluation of Expression and Phosphorylation of Progesterone Receptor in Endometrial Stromal Cells of Patients with Recurrent Implantation Failure Compared to Healthy Fertile Women. *Reprod Sci* 2021: **28(5)**; 1457-1465.

Hu C, Deng J, Liu M, Ni T, Chen ZJ, Yan J, and Li Y. Endometrial BMP2 Deficiency Impairs ITGB3-Mediated Trophoblast Invasion in Women With Repeated Implantation Failure. *Endocrinology* 2024: **165(3)**.

Hu S, Sun Z, Li B, Zhao H, Wang Y, Yao G, Li X, Bian X, Li TC, Vankelecom H*, et al.* iTRAQ-based Proteomic Analysis Unveils ACSL4 as a Novel Potential Regulator of Human Endometrial Receptivity. *Endocrinology (United States)* 2023: **164(3)**:164.

Huang C, Jiang Y, Zhou J, Yan Q, Jiang R, Cheng X, Xing J, Ding L, Sun J, Yan G*, et al.* Increased Krüppel-like factor 12 in recurrent implantation failure impairs endometrial decidualization by repressing Nur77 expression. *Reprod Biol Endocrinol* 2017: **15(1)**; 25.

Huang C, Liang P, Diao L, Liu C, Chen X, Li G, Chen C, and Zeng Y. Thyroid Autoimmunity is Associated with Decreased Cytotoxicity T Cells in Women with Repeated Implantation Failure. *Int J Environ Res Public Health* 2015: **12(9)**; 10352-10361.

Huang C, Sun H, Wang Z, Liu Y, Cheng X, Liu J, Jiang R, Zhang X, Zhen X, Zhou J*, et al.* Increased Krüppel-like factor 12 impairs embryo attachment via downregulation of leukemia inhibitory factor in women with recurrent implantation failure. *Cell Death Discov* 2018: **4**; 23.

Huang C, Xiang Z, Zhang Y, Li Y, Xu J, Zhang H, Zeng Y, and Tu W. NKG2D as a Cell Surface Marker on γδ-T Cells for Predicting Pregnancy Outcomes in Patients With Unexplained Repeated Implantation Failure. *Front Immunol* 2021: **12**; 631077.

Huang C, Zhang Q, Ni T, Zhou T, Lv C, Li Y, Yan J, and Chen ZJ. Deficiency of RARα Suppresses Decidualization via Downregulating CEBPB Transcription in Women With Recurrent Implantation Failure. *Front Endocrinol (Lausanne)* 2022: **13**; 753416.

Huang C, Zhang Y, Xiang Z, Li Y, Lin R, Xu J, Tu W, and Zeng Y. Granzyme B-expressing γδ-T and NK cells as a predictor of clinical pregnancy failure in patients with unexplained repeated implantation failure. *J Reprod Immunol* 2021: **144**; 103269.

Huang CC, Cheng TC, Chang HH, Chang CC, Chen CI, Liu JE, and Lee MS. Birth after the injection of sperm and the cytoplasm of tripronucleate zygotes into metaphase II oocytes in patients with repeated implantation failure after assisted fertilization procedures. *Fertility and sterility* 1999: **72(4)**; 702-706.

Huang J, Jin N, Qin H, Shi X, Liu Y, Cheung W, Wang CC, Chan TF, and Li TC. Transcriptomic profiles in peripheral blood between women with unexplained recurrent implantation failure and recurrent miscarriage and the correlation with endometrium: A pilot study. *PLoS One* 2017: **12(12)**; e0189159.

Huang J, Song N, Xia L, Tian L, Tan J, Chen Q, Zhu J, and Wu Q. Construction of lncRNA-related competing endogenous RNA network and identification of hub genes in recurrent implantation failure. *Reprod Biol Endocrinol* 2021: **19(1)**; 108.

Huang P, Yao C, Wei L, and Lin Z. The intrauterine perfusion of granulocyte-colony stimulating factor (G-CSF) before frozen-thawed embryo transfer in patients with two or more implantation failures. *Human fertility* 2022: **25(2)**; 301-305.

Huang Q, Mo L, Wang J, and Qin A. Oil-soluble contrast medium bathing attenuated endometrial inflammation and improved endometrial receptivity in women with recurrent implantation failure: a descriptive study. *BMC Women's Health* 2024: **24(1)**:326.

Huang Q, Wu H, Li M, Yang Y, and Fu X. Prednisone improves pregnancy outcome in repeated implantation failure by enhance regulatory T cells bias. *J Reprod Immunol* 2021: **143**; 103245.

Huang SY, Wang CJ, Soong YK, Wang HS, Wang ML, Lin CY, and Chang CL. Site-specific endometrial injury improves implantation and pregnancy in patients with repeated implantation failures. *Reproductive biology and endocrinology : RB&E* 2011: **9**; 140.

Hue HJ, Choi H, Lee HK, Lee JR, Jee BC, Choo CW, and Kim SK. Prevalence and confounders of chronic endometritis diagnosed using CD138 in patients with recurrent implantation failure. *Clin Exp Reprod Med* 2024: **51(2)**; 163-169.

Hur YJ, Yu EJ, Choe SA, Paek J, and Kim YS. Peripheral blood natural killer cell proportion and ovarian function in women with recurrent implantation failure. *Gynecol Endocrinol* 2020: **36(10)**; 922-925.

Hussain Mostafa M, Samir HM, and Sayed MS. Efficacy of sequential embryo transfer in improving pregnancy rate in women with repeated unexplained implantation failure. *Ginekologia i Poloznictwo* 2023: **18(1)**.

Hussaini S, Nayot D, Hartman M, and Dahan MH. Can endometrial thickness at baseline or maximum stimulated levels during IVF predict the presence of endometrial polyps in women with two failed embryo transfers? *Archives of Gynecology and Obstetrics* 2022: **307(5)**; 1645-1653.

Ichiyama T, Kuroda K, Nagai Y, Urushiyama D, Ohno M, Yamaguchi T, Nagayoshi M, Sakuraba Y, Yamasaki F, Hata K*, et al.* Analysis of vaginal and endometrial microbiota communities in infertile women with a history of repeated implantation failure. *Reprod Med Biol* 2021: **20(3)**; 334-344.

Inagaki N, Stern C, McBain J, Lopata A, Kornman L, and Wilkinson D. Analysis of intra-uterine cytokine concentration and matrix-metalloproteinase activity in women with recurrent failed embryo transfer. *Hum Reprod* 2003: **18(3)**; 608-615.

Ismail Madkour WA, Noah B, Zaheer H, Al-Bahr A, Abdelhamid AMS, Shaeer M, and Moawad A. Does sequential embryo transfer improve pregnancy rate in patients with repeated implantation failure? A randomized control study. *Middle east fertility society journal* 2015: **20(4)**; 255‐261.

Ivanov P, Ivanov P, Gacheva S, Konova E, and Komsa-Penkova R. [Implication of PAI-1 4G/5G polymorphism in recurrent implantation failure after IVF]. *Akush Ginekol (Sofiia)* 2014: **53(3)**; 25-29.

Ivanov P, Konova E, Gecheva S, Tavyatkovska T, Izmailov A, Komsa-Penkova R, Ivanov I, Golemanov G, Kovacheva K, and Simeonova M. [Factor V Leiden in women with repeated IVF failures]. *Akush Ginekol (Sofiia)* 2013: **52(6)**; 11-14.

Iwami N, Kawamata M, Ozawa N, Yamamoto T, Watanabe E, Mizuuchi M, Moriwaka O, and Kamiya H. Therapeutic intervention based on gene sequencing analysis of microbial 16S ribosomal RNA of the intrauterine microbiome improves pregnancy outcomes in IVF patients: a prospective cohort study. *Journal of assisted reproduction and genetics* 2023: **40(1)**; 125-135.

Iwasa T, Kuwahara A, Takeshita T, Taniguchi Y, Mikami M, and Irahara M. Preimplantation genetic testing for aneuploidy and chromosomal structural rearrangement: A summary of a nationwide study by the Japan Society of Obstetrics and Gynecology. *Reproductive Medicine and Biology* 2023: **22(1)**:e12518.

Jafarabadi M, Farbod Y, and Shariat M. Low-Level Laser Therapy for Improvement of In Vitro Fertilization Outcomes in Patients with Recurrent Implantation Failure: A Randomized Clinical Trial. *J Lasers Med Sci* 2024: **15**; e15.

Jahanbakhsh J, Mojtahedi MF, Moradi N, Fadaei R, Tehranian A, Rostami R, Kashani L, Moini A, Alizadeh-Fanalou S, Tond SB*, et al.* Circulating and Endometrial Profiles of miR-145, miR-155-5p, miR-224, MPP-5, and PECAM-1 Expression in Patients with Repeated Implantation Failure: A Case Control Study. *Cell Journal* 2023: **25(6)**; 427-436.

Ji M, Zhang L, Fu X, Xie W, Wu X, and Shu J. The outcomes of sequential embryo transfer in patients undergoing in vitro fertilization with frozen-thawed embryos: A retrospective study. *Journal of Obstetrics and Gynaecology Research* 2022: **48(10)**; 2563-2570.

Jia Y, Ai Z, Zhu X, Che Z, Pratikshya A, Tang S, and Zhang Q. Analysis of predictors of clinical pregnancy and live birth in patients with RIF treated with IVF-ET technology: a cohort study based on a propensity score approach. *Frontiers in Medicine* 2024: **11**:1348733.

Jia Y, Dong YJ, Sha YL, Cai SC, Diao LH, Qiu Z, Guo YH, Huang Y, Ye HX, and Liu S. Effectiveness comparison between endometrial receptivity array, immune profiling and the combination in treating patients with multiple implantation failure. *American journal of reproductive immunology* 2022: **87(3)**:e13513.

Jia Y, Huang Y, Ai ZH, Dong YJ, Guo YH, Yang Y, Zhang D, Ye HX, and Yang J. Exploring the effectiveness of endometrial receptivity array and immune profiling in patients with multiple implantation failure：A retrospective cohort study based on propensity score matching. *Journal of reproductive immunology* 2024: **163**:104218.

Jia Y, Sha YL, Qiu Z, Guo YH, Tan AX, Huang Y, Zhong Y, Dong YJ, and Ye HX. Comparison of the Effectiveness of Endometrial Receptivity Analysis (ERA) to Guide Personalized Embryo Transfer with Conventional Frozen Embryo Transfer in 281 Chinese Women with Recurrent Implantation Failure. *Medical Science Monitor* 2022: **28**:e935634.

Jiang H, Zhu M, Guo P, Bi K, Lu Z, Li C, Zhai M, Wang K, and Cao Y. Impaired myeloid-derived suppressor cells are associated with recurrent implantation failure: A case-control study. *J Reprod Immunol* 2021: **145**; 103316.

Jiang R, Ding L, Zhou J, Huang C, Zhang Q, Jiang Y, Liu J, Yan Q, Zhen X, Sun J*, et al.* Enhanced HOXA10 sumoylation inhibits embryo implantation in women with recurrent implantation failure. *Cell Death Discov* 2017: **3**; 17057.

Jiang RW, Yan GJ, Xing J, Wang ZL, Liu Y, Wu HY, Fan XS, Zhou JJ, Ding LJ, and Sun HX. Abnormal ratio of CD57(+) cells to CD56(+) cells in women with recurrent implantation failure. *American journal of reproductive immunology* 2017: **78(5)**:e12708; 8.

Johnston-MacAnanny EB, Hartnett J, Engmann LL, Nulsen JC, Sanders MM, and Benadiva CA. Chronic endometritis is a frequent finding in women with recurrent implantation failure after in vitro fertilization. *Fertil Steril* 2010: **93(2)**; 437-441.

Jung YW, Kim JO, Rah H, Kim JH, Kim YR, Lee Y, Lee WS, and Kim NK. Genetic variants of vascular endothelial growth factor are associated with recurrent implantation failure in Korean women. *Reprod Biomed Online* 2016: **32(2)**; 190-196.

Kadogami D, Nakaoka Y, and Morimoto Y. Use of a vaginal probiotic suppository and antibiotics to influence the composition of the endometrial microbiota. *Reprod Biol* 2020: **20(3)**; 307-314.

Kahraman S, Bahce M, Samli H, Imirzahoglu N, Yakisn K, Cengiz G, and Donmez E. Healthy births and ongoing pregnancies obtained by preimplantation genetic diagnosis in patients with advanced maternal age and recurrent implantation failure. *Human Reproduction* 2000: **15(9)**; 2003-2007.

Kahraman S, Benkhalifa M, Donmez E, Biricik A, Sertyel S, Findikli N, and Berkil H. The results of aneuploidy screening in 276 couples undergoing assisted reproductive techniques. *Prenat Diagn* 2004: **24(4)**; 307-311.

Kalem MN, Akgun N, Kalem Z, Bakirarar B, and Celik T. Chemokine (C-C motif) ligand-2 (CCL2) and oxidative stress markers in recurrent pregnancy loss and repeated implantation failure. *Journal of assisted reproduction and genetics* 2017: **34(11)**; 1501-1506.

Kalem Z, Kalem MN, Bakirarar B, Kent E, Makrigiannakis A, and Gurgan T. Intrauterine G-CSF Administration in Recurrent Implantation Failure (RIF): An Rct. *Scientific reports* 2020: **10(1)**:5139; 7.

Kanazawa E, Nakashima A, Yonemoto K, Otsuka M, Yoshioka N, Kuramoto T, Mitao H, Imaishi H, Komai K, and Ushijima K. Injury to the endometrium prior to the frozen–thawed embryo transfer cycle improves pregnancy rates in patients with repeated implantation failure. *Journal of Obstetrics and Gynaecology Research* 2017: **43(1)**; 128-134.

Kanyo K, Zeke J, Kriston R, Szücs Z, Cseh S, Somoskoi B, and Konc J. The impact of laser-assisted hatching on the outcome of frozen human embryo transfer cycles. *Zygote (Cambridge, England)* 2016: **24(5)**; 742‐747.

Karabulut S, Aksunger O, Korkmaz O, Eren Gozel H, and Keskin I. Intracytoplasmic morphologically selected sperm injection, but for whom? *Zygote* 2019: **27(5)**; 299-304.

Karaer A, Tuncay G, Uysal O, Semerci Sevimli T, Sahin N, Karabulut U, and Sariboyaci AE. The role of prokineticins in recurrent implantation failure. *J Gynecol Obstet Hum Reprod* 2020: **49(9)**; 101835.

Karimi A, Mokhtar S, Sadeghi MR, Zafardoust S, Ataei M, Ghoodjani A, Mohazzab A, Nazari F, and Ansaripour S. May intrauterine granulocyte colony stimulating factor improve clinical & ongoing pregnancy & live birth rates in unexplained repeated implantation failure patients? a randomized clinical trial. *European journal of molecular and clinical medicine* 2020: **7(2)**; 82‐87.

Karimzadeh MA, Ayazi Rozbahani M, and Tabibnejad N. Endometrial local injury improves the pregnancy rate among recurrent implantation failure patients undergoing in vitro fertilisation/intra cytoplasmic sperm injection: a randomised clinical trial. *Australian & New Zealand journal of obstetrics & gynaecology* 2009: **49(6)**; 677‐680.

Kasvandik S, Saarma M, Kaart T, Rooda I, Velthut-Meikas A, Ehrenberg A, Gemzell K, Lalitkumar PG, Salumets A, and Peters M. Uterine Fluid Proteins for Minimally Invasive Assessment of Endometrial Receptivity. *J Clin Endocrinol Metab* 2020: **105(1)**.

Kato K, Ezoe K, Onogi S, Ito S, Egawa R, Aoyama N, Kuroda T, Kuwahara A, Iwasa T, Takeshita T*, et al.* Comparison of 1-year cumulative live birth and perinatal outcomes following single blastocyst transfer with or without preimplantation genetic testing for aneuploidy: a propensity score-matched study. *Journal of assisted reproduction and genetics* 2023: **40(11)**; 2669-2680.

Kato K, Kuroda T, Yamadera-Egawa R, Ezoe K, Aoyama N, Usami A, Miki T, Yamamoto T, and Takeshita T. Preimplantation Genetic Testing for Aneuploidy for Recurrent Pregnancy Loss and Recurrent Implantation Failure in Minimal Ovarian Stimulation Cycle for Women Aged 35-42 Years: Live Birth Rate, Developmental Follow-up of Children, and Embryo Ranking. *Reproductive Sciences* 2023: **30(3)**; 974-983.

Kay C, Jeyendran RS, and Coulam CB. p53 tumour suppressor gene polymorphism is associated with recurrent implantation failure. *Reprod Biomed Online* 2006: **13(4)**; 492-496.

Keburiya LK, Smol’nikova VY, Priputnevich TV, Murav’eva VV, Trofimov DY, Shubina ES, and Kochetkova TO. Uterine microbiota and implantation failure: Is there a link? *Akusherstvo i Ginekologiya (Russian Federation)* 2021: **2021(7)**; 133-143.

Keburiya LK, Smolnikova VY, Priputnevich TV, Muravieva VV, Gordeev AB, Trofimov DY, Shubina ES, Kochetkova TO, Rogacheva MS, Kalinina EA*, et al.* Does the uterine microbiota affect the reproductive outcomes in women with recurrent implantation failures? *BMC Women's Health* 2022: **22(1)**:168.

Khairy M, Harb H, Eapen A, Melo P, Kazem R, Rajkhowa M, Ndukuwe G, and Coomarasamy A. The use of immunomodulation therapy in women with recurrent implantation failure undergoing assisted conception: A multicentre cohort study. *American journal of reproductive immunology* 2024: **91(2)**:e13819.

Khan NLA, Nafee T, Shao TT, Hart AR, Elliott S, Ola B, Heath PR, and Fazeli A. Dysregulation in Multiple Transcriptomic Endometrial Pathways Is Associated with Recurrent Implantation Failure and Recurrent Early Pregnancy Loss. *International Journal of Molecular Sciences* 2022: **23(24)**:16051; 20.

Kharamani A, Mashayekhi F, and Salehi Z. Association of Fibroblast Growth Factor-1 Promoter Polymorphism and its Serum Concentrations with Repeated Implantation Failure after In vitro Fertilisation: A Cross-sectional Study. *J Hum Reprod Sci* 2024: **17(2)**; 121-127.

Khayat S, Elliott B, and Dahan MH. Management of recurrent implantation failure by gonadotropin-releasing hormone agonist and aromatase inhibitor suppression, in women without evidence of endometriosis. *Gynecol Endocrinol* 2019: **35(3)**; 267-270.

Kim JO, Ahn EH, Sakong JH, An HJ, Park HS, Kim YR, Lee JR, Lee WS, and Kim NK. Association of miR-27aA>G, miR-423C>a, miR-449bA>G, and miR-604A>G Polymorphisms with Risk of Recurrent Implantation Failure. *Reprod Sci* 2020: **27(1)**; 29-38.

Kim JW, Lee SY, Hur CY, Lim JH, and Park CK. Clinical outcomes of preimplantation genetic testing for aneuploidy in high-risk patients: a retrospective cohort study. *Clin Exp Reprod Med* 2024: **51(1)**; 75-84.

Kitaya K and Ishikawa T. Genital tract dysbiosis in infertile women with a history of repeated implantation failure and pilot study for reproductive outcomes following oral enteric coating lactoferrin supplementation. *Archives of Gynecology and Obstetrics* 2022: **306(5)**; 1761-1769.

Kitaya K, Matsubayashi H, Takaya Y, Nishiyama R, Yamaguchi K, and Ishikawa T. Clinical background affecting pregnancy outcome following local endometrial injury in infertile patients with repeated implantation failure. *Gynecol Endocrinol* 2016: **32(7)**; 587-590.

Kitaya K, Matsubayashi H, Takaya Y, Nishiyama R, Yamaguchi K, Takeuchi T, and Ishikawa T. Live birth rate following oral antibiotic treatment for chronic endometritis in infertile women with repeated implantation failure. *Am J Reprod Immunol* 2017: **78(5)**.

Kitaya K, Nagai Y, Arai W, Sakuraba Y, and Ishikawa T. Characterization of Microbiota in Endometrial Fluid and Vaginal Secretions in Infertile Women with Repeated Implantation Failure. *Mediators Inflamm* 2019: **2019**; 4893437.

Kitaya K, Tada Y, Hayashi T, Taguchi S, Funabiki M, and Nakamura Y. Comprehensive endometrial immunoglobulin subclass analysis in infertile women suffering from repeated implantation failure with or without chronic endometritis. *Am J Reprod Immunol* 2014: **72(4)**; 386-391.

Kitaya K, Tanaka SE, Sakuraba Y, and Ishikawa T. Multi-drug-resistant chronic endometritis in infertile women with repeated implantation failure: trend over the decade and pilot study for third-line oral antibiotic treatment. *Journal of assisted reproduction and genetics* 2022: **39(8)**; 1839-1848.

Kling C and Kabelitz D. HCMV seroprevalence in couples under infertility treatment. *Arch Gynecol Obstet* 2015: **292(2)**; 439-443.

Kling C, Magez-Zunker J, Jenisch S, and Kabelitz D. Effect of allogeneic leukocyte immunization on consecutive IVF/ICSI-treatment for failure in the in-vitro-fertilization program. *Geburtshilfe und Frauenheilkunde* 2002: **62(7)**; 661-667.

Kling C, Schmutzler A, Wilke G, Hedderich J, and Kabelitz D. IVF prognosis after recurrent implantation failure: Experience of German centers. *Geburtshilfe und Frauenheilkunde* 2008: **68(5)**; 505-511.

Kling C, Schmutzler A, Wilke G, Hedderich J, and Kabelitz D. Two-year outcome after recurrent implantation failure: prognostic factors and additional interventions. *Arch Gynecol Obstet* 2008: **278(2)**; 135-142.

Ko EJ, Shin JE, Lee JY, Ryu CS, Hwang JY, Kim YR, Ahn EH, Kim JH, and Kim NK. Association of Polymorphisms in FSHR, INHA, ESR1, and BMP15 with Recurrent Implantation Failure. *Biomedicines* 2023: **11(5)**.

Kolanska K, Bendifallah S, Cohen J, Placais L, Selleret L, Johanet C, Suner L, Delhommeau F, Chabbert-Buffet N, Darai E*, et al.* Unexplained recurrent implantation failures: Predictive factors of pregnancy and therapeutic management from a French multicentre study. *Journal of reproductive immunology* 2021: **145**:103313; 7.

Kolanska K, Suner L, Cohen J, Ben Kraiem Y, Placais L, Fain O, Bornes M, Selleret L, Delhommeau F, Feger F*, et al.* Proportion of Cytotoxic Peripheral Blood Natural Killer Cells and T-Cell Large Granular Lymphocytes in Recurrent Miscarriage and Repeated Implantation Failure: Case–Control Study and Meta-analysis. *Archivum Immunologiae et Therapiae Experimentalis* 2019: **67(4)**; 225-236.

Koler M, Achache H, Tsafrir A, Smith Y, Revel A, and Reich R. Disrupted gene pattern in patients with repeated in vitro fertilization (IVF) failure. *Hum Reprod* 2009: **24(10)**; 2541-2548.

Koot YE, van Hooff SR, Boomsma CM, van Leenen D, Groot Koerkamp MJ, Goddijn M, Eijkemans MJ, Fauser BC, Holstege FC, and Macklon NS. An endometrial gene expression signature accurately predicts recurrent implantation failure after IVF. *Sci Rep* 2016: **6**; 19411.

Koot YEM, Hviid Saxtorph M, Goddijn M, de Bever S, Eijkemans MJC, Wely MV, van der Veen F, Fauser B, and Macklon NS. What is the prognosis for a live birth after unexplained recurrent implantation failure following IVF/ICSI? *Hum Reprod* 2019: **34(10)**; 2044-2052.

Koushaeian L, Ghorbani F, Ahmadi M, Eghbal-Fard S, Zamani M, Danaii S, Yousefi B, Jadidi-Niaragh F, Hamdi K, and Yousefi M. The role of IL-10-producing B cells in repeated implantation failure patients with cellular immune abnormalities. *Immunol Lett* 2019: **214**; 16-22.

Kuroda K, Horikawa T, Moriyama A, Ojiro Y, Takamizawa S, Watanabe H, Maruyama T, Nojiri S, Nakagawa K, and Sugiyama R. Therapeutic efficacy of the optimization of thyroid function, thrombophilia, immunity and uterine milieu (OPTIMUM) treatment strategy on pregnancy outcomes after single euploid blastocyst transfer in advanced age women with recurrent reproductive failure. *Reproductive Medicine and Biology* 2023: **22(1)**:e12554.

Kuroda K, Matsumura Y, Ikemoto Y, Segawa T, Hashimoto T, Fukuda J, Nakagawa K, Uchida T, Ochiai A, Horimoto Y*, et al.* Analysis of the risk factors and treatment for repeated implantation failure: OPtimization of Thyroid function, IMmunity, and Uterine Milieu (OPTIMUM) treatment strategy. *Am J Reprod Immunol* 2021: **85(5)**; e13376.

Kuroda K, Nakagawa K, Horikawa T, Moriyama A, Ojiro Y, Takamizawa S, Ochiai A, Matsumura Y, Ikemoto Y, Yamaguchi K*, et al.* Increasing number of implantation failures and pregnancy losses associated with elevated Th1/Th2 cell ratio. *Am J Reprod Immunol* 2021: **86(3)**; e13429.

Kuroshli Z, Gourabi H, Bazrgar M, Sanati M, and Zamani Esteki M. The Relationship between HLA-G Gene Polymorphisms and Repeated Implantation Failure in Infertile Couples Undergoing Assisted Reproductive Technique. *Iran J Allergy Asthma Immunol* 2015: **14(5)**; 535-542.

Kushniruk N, Stastna A, Fait T, and Lenertova T. Feasible Influence of G-CSF on Clinical Pregnancy Outcome in Oocyte Donation Cycles for Patients with Recurrent Implantation Failure. *Medicina (Kaunas)* 2024: **60(6)**.

Kusumi M, Ihana T, Kurosawa T, Ohashi Y, and Tsutsumi O. Intrauterine administration of platelet-rich plasma improves embryo implantation by increasing the endometrial thickness in women with repeated implantation failure: A single-arm self-controlled trial. *Reprod Med Biol* 2020: **19(4)**; 350-356.

Kwon MJ, Kim JH, Kim KJ, Ko EJ, Lee JY, Ryu CS, Ha YH, Kim YR, and Kim NK. Genetic Association between Inflammatory-Related Polymorphism in STAT3, IL-1β, IL-6, TNF-α and Idiopathic Recurrent Implantation Failure. *Genes (Basel)* 2023: **14(8)**.

Lacconi V, Massimiani M, Paglione L, Antonaci D, Meneghini C, Klinger FG, Fazleabas A, Stuhlmann H, Rago R, Ticconi C*, et al.* Characterization of epidermal growth factor-like domain 7 (EGFL7) expression in normal endometrium and in the endometrium of women with poor reproductive outcomes. *Human Reproduction* 2023: **38(7)**; 1345-1358.

Lai YY, Liu LY, Wu YN, Huang L, Zheng XY, Gan D, Yu SY, Zhong Y, Liang FR, Zhou Y*, et al.* Immediate Effect of Electro-acupuncture on Endometrial Blood Flow in Patients with Recurrent Implantation Failure: A Randomized Controlled Trial. *Chinese journal of integrative medicine* 2024: **30(4)**; 291-298.

Lai ZZ, Wang Y, Zhou WJ, Liang Z, Shi JW, Yang HL, Xie F, Chen WD, Zhu R, Zhang C*, et al.* Single-cell transcriptome profiling of the human endometrium of patients with recurrent implantation failure. *Theranostics* 2022: **12(15)**; 6527-6547.

Lai ZZ, Zhang J, Zhou WJ, Shi JW, Yang HL, Yang SL, Wu JN, Xie F, Zhang T, and Li MQ. Identification of potential biomarkers and immune infiltration characteristics in recurrent implantation failure using bioinformatics analysis. *Frontiers in Immunology* 2023: **14**:992765.

Lambert M, Hocké C, Jimenez C, Frantz S, Papaxanthos A, and Creux H. [Repeated in vitro fertilization failure: Abnormalities identified in the diagnostic assessment]. *Gynecol Obstet Fertil* 2016: **44(10)**; 565-571.

Landwchr C, Montag M, van der Ven K, and Weber RG. Rapid comparative genomic hybridization protocol for prenatal diagnosis and its application to aneuploidy screening of human polar bodies. *Fertility and sterility* 2008: **90(3)**; 488-496.

Lapides L, Klein M, Belusakova V, Csobonyeiova M, Varga I, and Babal P. Uterine Natural Killer Cells in the Context of Implantation: Immunohistochemical Analysis of Endometrial Samples from Women with Habitual Abortion and Recurrent Implantation Failure. *Physiological research* 2022: **71(Supplement 1)**; S99-S105.

Lapides L, Varga I, Klein M, Rybanska L, Belusakova V, and Babal P. When Less Is More - Pipelle Endometrial Sampling for Quantification of Uterine Natural Killer Cells in Patients With Recurrent Implantation Failure or Habitual Abortion. *Physiological research* 2022: **71(Supplement 1)**; S65-S73.

Lashley L, van der Keur C, van Beelen E, Schaap R, van der Westerlaken LAJ, Scherjon SA, and Claas FHJ. Stronger T-Cell Alloreactivity and Diminished Suppressive Capacity of Peripheral Regulatory T Cells in Infertile Women Undergoing In Vitro Fertilization. *American journal of reproductive immunology* 2015: **74(3)**; 268-278.

Lashley L, van der Westerlaken LAJ, Haasnoot GW, Drabbels JJM, Spruyt-Gerritse MJ, Scherjon SA, and Claas FHJ. Maternal HLA-C2 and 14bp insertion in HLA-G is associated with recurrent implantation failure after in vitro fertilization treatment. *Tissue Antigens* 2014: **84(6)**; 536-544.

Lédée N, Chaouat G, Serazin V, Lombroso R, Dubanchet S, Oger P, Louafi N, and Ville Y. Endometrial vascularity by three-dimensional power Doppler ultrasound and cytokines: a complementary approach to assess uterine receptivity. *Journal of reproductive immunology* 2008: **77(1)**; 57-62.

Lédée N, Munaut C, Aubert J, Sérazin V, Rahmati M, Chaouat G, Sandra O, and Foidart JM. Specific and extensive endometrial deregulation is present before conception in IVF/ICSI repeated implantation failures (IF) or recurrent miscarriages. *Journal of Pathology* 2011: **225(4)**; 554-564.

Lédée N, Petitbarat M, Prat-Ellenberg L, Dray G, Cassuto GN, Chevrier L, Kazhalawi A, Vezmar K, and Chaouat G. Endometrial Immune Profiling: A Method to Design Personalized Care in Assisted Reproductive Medicine. *Frontiers in Immunology* 2020: **11**:1032.

Lédée N, Vasseur C, Petitbarat M, Chevrier L, Vezmar K, Dray G, Chenière S, Lobersztajn A, Vitoux D, Cassuto GN*, et al.* Intralipid® may represent a new hope for patients with reproductive failures and simultaneously an over-immune endometrial activation. *Journal of reproductive immunology* 2018: **130**; 18-22.

Lédée-Bataille N, Bonnet-Chea K, Hosny G, Dubanchet S, Frydman R, and Chaouat G. Role of the endometrial tripod interleukin-18, -15, and -12 in inadequate uterine receptivity in patients with a history of repeated in vitro fertilization-embryo transfer failure. *Fertil Steril* 2005: **83(3)**; 598-605.

Lédée-Bataille N, Doumerc S, Olivennes F, Kadoch J, Chaouat G, and Frydman R. Ultrasonic diagnosis in repeated implantation failure in assisted reproduction medicine. *Journal de Gynecologie Obstetrique et Biologie de la Reproduction* 2001: **30(8)**; 747-752.

Lédée-Bataille N, Dubanchet S, Coulomb-L'hermine A, Durand-Gasselin I, Frydman R, and Chaouat G. A new role for natural killer cells, interleukin (IL)-12, and IL-18 in repeated implantation failure after in vitro fertilization. *Fertility and sterility* 2004: **81(1)**; 59‐65.

Lee CI, Wu CH, Pai YP, Chang YJ, Chen CI, Lee TH, and Lee MS. Performance of preimplantation genetic testing for aneuploidy in IVF cycles for patients with advanced maternal age, repeat implantation failure, and idiopathic recurrent miscarriage. *Taiwan J Obstet Gynecol* 2019: **58(2)**; 239-243.

Lee HA, Ahn EH, Jang HG, Kim JO, Kim JH, Lee YB, Lee WS, and Kim NK. Association Between miR-605A>G, miR-608G>C, miR-631I>D, miR-938C>T, and miR-1302-3C>T Polymorphisms and Risk of Recurrent Implantation Failure. *Reprod Sci* 2019: **26(4)**; 469-475.

Lee HK, Moon KY, Paik H, and Jee BC. Factors affecting the ongoing pregnancy rate in women with repeated implantation failure undergoing an endometrial receptivity array. *Clin Exp Reprod Med* 2023: **50(4)**; 277-284.

Lee JH, Ahn EH, Kwon MJ, Ryu CS, Ha YH, Ko EJ, Lee JY, Hwang JY, Kim JH, Kim YR*, et al.* Genetic Correlation of miRNA Polymorphisms and STAT3 Signaling Pathway with Recurrent Implantation Failure in the Korean Population. *International Journal of Molecular Sciences* 2023: **24(23)**:16794.

Lee JH, Han JE, Kim YS, Won HJ, Cho CH, and Kwak IP. Efficacy of Assisted Hatching by Laser in Human IVF-ET Program. *Korean journal of reproductive medicine* 2008: **35(3)**; 193‐202.

Lee JW, Cha JH, Shin SH, Kim YJ, Lee SK, Park CK, Pak KA, Yoon JS, and Park SY. Effects of laser-assisted thinning versus opening on clinical outcomes according to maternal age in patients with repeated implantation failure. *Lasers Med Sci* 2019: **34(9)**; 1889-1895.

Lee JY, Ahn EH, Kim JO, Park HS, Ryu CS, Kim JH, Kim YR, Lee WS, and Kim NK. Associations between microRNA (miR-25, miR-32, miR-125, and miR-222) polymorphisms and recurrent implantation failure in Korean women. *Hum Genomics* 2019: **13(1)**; 68.

Lee JY, Ahn EH, Park HW, Kim JH, Kim YR, Lee WS, and Kim NK. Association between HOX Transcript Antisense RNA Single-Nucleotide Variants and Recurrent Implantation Failure. *Int J Mol Sci* 2021: **22(6)**.

Lee S, Arffman RK, Komsi EK, Lindgren O, Kemppainen J, Kask K, Saare M, Salumets A, and Piltonen TT. Dynamic changes in AI-based analysis of endometrial cellular composition: Analysis of PCOS and RIF endometrium. *J Pathol Inform* 2024: **15**; 100364.

Lee S, Arffman RK, Komsi EK, Lindgren O, Kemppainen JA, Metsola H, Ahtikoski A, Kask K, Saare M, Salumets A*, et al.* AI-algorithm training and validation for endometrial CD138+ cells in infertility-associated conditions; polycystic ovary syndrome (PCOS) and recurrent implantation failure (RIF). *medRxiv.* 2023: **05**.

Lee Y, Ahn EH, Ryu CS, Kim JO, An HJ, Cho SH, Kim JH, Kim YR, Lee WS, and Kim NK. Association between microRNA machinery gene polymorphisms and recurrent implantation failure. *Exp Ther Med* 2020: **19(4)**; 3113-3123.

Levran D, Farhi J, Nahum H, Royburt M, Glezerman M, and Weissman A. Prospective evaluation of blastocyst stage transfer vs. zygote intrafallopian tube transfer in patients with repeated implantation failure. *Fertil Steril* 2002: **77(5)**; 971-977.

Li B, Yan YP, Liang C, He YY, Wang Y, Li MY, Chen ST, Li Y, Liu AX, Yan GJ*, et al.* Primary Cilia Restrain PI3K-AKT Signaling to Orchestrate Human Decidualization. *International Journal of Molecular Sciences* 2022: **23(24)**:15573.

Li F, Gao W, Li Y, Wang Y, Liu L, and Zhang X. Potential Biomarkers and Endometrial Immune Microenvironment in Recurrent Implantation Failure. *Biomolecules* 2023: **13(3)**:406.

Li HX, Su N, Zhu YQ, Wang W, Cai MH, Luo XH, Xia W, and Quan S. Growth hormone inhibits the JAK/STAT3 pathway by regulating SOCS1 in endometrial cells in vitro: A clue to enhance endometrial receptivity in recurrent implantation failure. *European Journal of Histochemistry* 2023: **67(1)**:3580; 8.

Li J, Li X, Ding J, Zhao J, Chen J, Guan F, Deng H, Zhou M, Han Y, Xiao Z*, et al.* Analysis of pregnancy outcomes in patients with recurrent implantation failure complicated with chronic endometritis. *Frontiers in Cell and Developmental Biology* 2023: **11**:1088586.

Li J, Mo S, Lin Z, and Shi Q. Atosiban application in fresh ET cycle is effective for women undergoing repeated embryo implantation failures, especially for advanced-age obese patients. *Scientific reports* 2023: **13(1)**:23044.

Li J, Qi J, Yao G, Zhu Q, Li X, Xu R, Zhu Z, Zhao H, Wang Y, Ding Y*, et al.* Deficiency of Sirtuin 1 Impedes Endometrial Decidualization in Recurrent Implantation Failure Patients. *Front Cell Dev Biol* 2021: **9**; 598364.

Li LF, Kou ZJ, Fu YJ, Liang LL, Liu L, and Zhang XH. Clinical outcomes of personalized frozen-thawed embryo transfer timing for patients with recurrent implantation failure. *Annals of Translational Medicine* 2022: **10(3)**:131; 8.

Li M, Hu J, Yao L, and Gao M. Decreased ANGPTL4 impairs endometrial angiogenesis during peri‐implantation period in patients with recurrent implantation failure. *Journal of Cellular and Molecular Medicine* 2020: **24(18)**; 10730-10743.

Li N, Guan YC, Liu JJ, Ren BN, Du YL, Wang KX, Zhang YJ, and Lou H. History of Recurrent Implantation Failure is Associated With the Incidence of Adverse Perinatal Outcomes in Singleton Live Births Following Frozen-Thawed Embryo Transfer Cycles. *Frontiers in Endocrinology* 2022: **12**:774646; 7.

Li N, Zhang Y, Chen Y, Huang L, Tan Z, Ban X, Zhou L, Xu C, Qiu Y, and Li R. Personalized embryo transfer guided by rsERT improves pregnancy outcomes in patients with repeated implantation failure. *Frontiers in Medicine* 2024: **11**:1369317.

Li Q, Zhang Y, and Zou L. The effect of low-molecular-weight heparin on immune balance of patients with repeated implantation failure during the implantation window. *Cellular and Molecular Biology* 2023: **69(4)**; 112-115.

Li S, Wang J, Cheng Y, Zhou D, Yin T, Xu W, Yu N, and Yang J. Intrauterine administration of hCG-activated autologous human peripheral blood mononuclear cells (PBMC) promotes live birth rates in frozen/thawed embryo transfer cycles of patients with repeated implantation failure. *J Reprod Immunol* 2017: **119**; 15-22.

Li T, Greenblatt EM, Shin ME, Brown TJ, and Chan C. Endometrial laminin subunit beta-3 expression associates with reproductive outcome in patients with repeated implantation failure. *J Assist Reprod Genet* 2021: **38(7)**; 1835-1842.

Li T, Liu X, Yang X, Wang N, Wang Y, Li W, Liang X, and Huang R. Comparison of the efficacy of depot GnRH agonist protocol and the GnRH antagonist protocol in patients with repeated IVF failure: a retrospective cohort study. *Human fertility* 2023: **26(5)**; 1299-1306.

Li X, Du Y, Han X, Wang H, Sheng Y, Lian F, and Lian Q. Efficacy of atosiban for repeated implantation failure in frozen embryo transfer cycles. *Scientific reports* 2023: **13(1)**; 9277.

Li X, Zeng C, Wu P, Shang J, and Xue Q. Predictive value of D-dimer in patients with unexplained recurrent implantation failure during freeze-thaw embryo transfer cycles. *American journal of reproductive immunology* 2023: **90(1)**:e13710.

Li X, Zeng Y, He J, Luo B, Lu X, Zhu L, Yang Z, Cai F, Chen SA, and Luo Y. The optimal frozen embryo transfer strategy for the recurrent implantation failure patient without blastocyst freezing: thawing day 3 embryos and culturing to day 5 blastocysts. *Zygote (Cambridge, England)* 2023: **31(6)**; 596-604.

Li Y, Wen Q, Hu J, Liao J, Fan X, Chen H, Zhao Q, Lu GX, Lin G, and Gong F. Histological endometrial dating: a reliable tool for personalized frozen-thawed embryo transfer in patients with repeated implantation failure in natural cycles. *BMC Pregnancy and Childbirth* 2023: **23(1)**:199.

Li Y, Yu S, Huang C, Lian R, Chen C, Liu S, Li L, Diao L, Markert UR, and Zeng Y. Evaluation of peripheral and uterine immune status of chronic endometritis in patients with recurrent reproductive failure. *Fertil Steril* 2020: **113(1)**; 187-196.e181.

Li Y, Zhang L, Yu P, Cai X, Li N, and Ma B. The efficacy of sequential day 3 embryo and blastocyst transfer in patients with repeated implantation failure. *European Journal of Obstetrics and Gynecology and Reproductive Biology* 2023: **283**; 32-36.

Li YY, Xu YH, Yu SY, Lin SL, Chen WR, Lian RC, Diao LH, Sun HX, Ding LJ, and Zeng Y. Chronic endometritis impairs embryo implantation in patients with repeated implantation failure: A retrospective study. *Taiwanese Journal of Obstetrics & Gynecology* 2022: **61(6)**; 984-988.

Li Z, Wang X, Guan Y, Yu X, Liu J, and Zhang Z. Uterine artery blood flow and microvessel density by vaginal color Doppler ultrasonography in embryo implantation failure. *Exp Ther Med* 2017: **14(5)**; 4797-4800.

Liang PY, Diao LH, Huang CY, Lian RC, Chen X, Li GG, Zhao J, Li YY, He XB, and Zeng Y. The pro-inflammatory and anti-inflammatory cytokine profile in peripheral blood of women with recurrent implantation failure. *Reprod Biomed Online* 2015: **31(6)**; 823-826.

Liang PY, Yin B, Cai J, Hu XD, Song C, Wu TH, Zhao J, Li GG, and Zeng Y. Increased circulating Th1/Th2 ratios but not other lymphocyte subsets during controlled ovarian stimulation are linked to subsequent implantation failure after transfer of in vitro fertilized embryos. *Am J Reprod Immunol* 2015: **73(1)**; 12-21.

Lin JZ and Lin N. Three Oxidative Stress-Related Genes That Associate Endometrial Immune Cells Are Considered as Potential Biomarkers for the Prediction of Unexplained Recurrent Implantation Failure. *Frontiers in Immunology* 2022: **13**:902268; 10.

Lin N and Lin JZ. Identification of long non-coding RNA biomarkers and signature scoring, with competing endogenous RNA networks- targeted drug candidates for recurrent implantation failure. *Human Fertiity* 2021: **25(5)**; 983-992.

Lin PY, Lee CI, Chen YC, Cheng EH, Huang CC, Chen CI, Lee TH, Lee YJ, and Lee MS. Factors Affecting the Potential Efficacy of Intrauterine Platelet-Rich Plasma Infusion on Thin Endometrium in Women with Recurrent Implantation Failure. *Journal of Personalized Medicine* 2023: **13(9)**:1419.

Liu B, Mariee N, Laird S, Smith J, Li J, and Li TC. The prognostic value of uNK cell count and histological dating in the mid-luteal phase of women with reproductive failure. *Eur J Obstet Gynecol Reprod Biol* 2014: **181**; 171-175.

Liu C, Li L, Wang M, Shui S, Yao H, Sui C, and Zhang H. Endometrial extracellular vesicles of recurrent implantation failure patients inhibit the proliferation, migration, and invasion of HTR8/SVneo cells. *Journal of assisted reproduction and genetics* 2021: **38(4)**; 825-833.

Liu C, Li Y, Li L, Shui S, Yang L, Sui C, and Zhang H. Aberrant expression of oxytocin receptor in endometrium and decidua in women who have experienced recurrent implantation failure. *F and S Science* 2020: **1(2)**; 183-187.

Liu C, Yao W, Yao J, Li L, Yang L, Zhang H, and Sui C. Endometrial extracellular vesicles from women with recurrent implantation failure attenuate the growth and invasion of embryos. *Fertil Steril* 2020: **114(2)**; 416-425.

Liu L, Liu XY, Luo S, Wang XH, Xu ZY, and Hao CF. Platelet-Rich Plasma Improves Pregnancy Rate and Repairs Endometrial Injury in Patients with Repeated Implantation Failure. *Journal of Biomaterials and Tissue Engineering* 2021: **11(5)**; 879-885.

Liu R, Chen L, Zhao X, Bao L, Wei R, and Wu X. MUC1 promotes RIF by regulating macrophage ROS-SHP2 signaling pathway to up-regulate inflammatory response and inhibit angiogenesis. *Aging* 2024: **16(4)**; 3790-3802.

Liu S, Wei H, Li Y, Huang C, Lian R, Xu J, Chen L, and Zeng Y. Downregulation of ILT4(+) dendritic cells in recurrent miscarriage and recurrent implantation failure. *Am J Reprod Immunol* 2018: **80(4)**; e12998.

Liu X, Ma D, Wang W, Qu Q, Zhang N, Wang X, Fang J, Ma Z, and Hao C. Intrauterine administration of human chorionic gonadotropin improves the live birth rates of patients with repeated implantation failure in frozen-thawed blastocyst transfer cycles by increasing the percentage of peripheral regulatory T cells. *Arch Gynecol Obstet* 2019: **299(4)**; 1165-1172.

Liu X, Zhao H, Li W, Bao H, Qu Q, and Ma D. Up-regulation of miR-145 may contribute to repeated implantation failure after IVF–embryo transfer by targeting PAI-1. *Reproductive biomedicine online* 2020: **40(5)**; 627-636.

Liu Y, Chen X, Huang J, Wang CC, Yu MY, Laird S, and Li TC. Comparison of the prevalence of chronic endometritis as determined by means of different diagnostic methods in women with and without reproductive failure. *Fertil Steril* 2018: **109(5)**; 832-839.

Liu Y, Lan X, Lu J, Zhang Q, Zhou T, Ni T, and Yan J. Preimplantation Genetic Testing for Aneuploidy Could Not Improve Cumulative Live Birth Rate Among 705 Couples with Unexplained Recurrent Implantation Failure. *Application of Clinical Genetics* 2024: **17**; 1-13.

Liu Z, Lai S, Qu Q, Liu X, Zhang W, Zhao D, He S, Sun Y, and Bao H. Analysis of weighted gene co-expression networks and clinical validation identify hub genes and immune cell infiltration in the endometrial cells of patients with recurrent implantation failure. *Front Genet* 2024: **15**; 1292757.

Lledo B, Turienzo A, Ortiz JA, Morales R, Ten J, Llácer J, and Bernabeu R. Negative effect of P72 polymorphism on p53 gene in IVF outcome in patients with repeated implantation failure and pregnancy loss. *J Assist Reprod Genet* 2014: **31(2)**; 169-172.

Lodigiani C, Di Micco P, Ferrazzi P, Librè L, Arfuso V, Polatti F, Benigna M, Rossini R, Morenghi E, Rota L*, et al.* Low-molecular-weight heparin in women with repeated implantation failure. *Womens Health (Lond)* 2011: **7(4)**; 425-431.

Long N, Liu N, Liu XL, Li J, Cai BY, and Cai X. Endometrial expression of telomerase, progesterone, and estrogen receptors during the implantation window in patients with recurrent implantation failure. *Genet Mol Res* 2016: **15(2)**.

Lozano FM, Lledó B, Morales R, Cascales A, Hortal M, Bernabeu A, and Bernabeu R. Characterization of the Endometrial Microbiome in Patients with Recurrent Implantation Failure. *Microorganisms* 2023: **11(3)**.

Lu XM, Xu C, Wang L, Sun NX, Zhang Q, Guan PF, and Li W. Effect of laser-assisted hatching on outcome of frozen-thawed embryo transfer for patients with previous repeated implantation failure. *Academic journal of second military medical university* 2016: **37(1)**; 106‐110.

Lu Y, Mao X, He Y, Wang Y, and Sun Y. Efficacy of endometrial receptivity testing for recurrent implantation failure in patients with euploid embryo transfers: study protocol for a randomized controlled trial. *Trials* 2024: **25(1)**:348.

Lu YK, Tu ZR, Wang LY, Gao RF, and Duan RY. Effects of laser-assisted hatching on the clinical outcome of freeze-thawedcleavage embryo transfer in patients with repeated implantation failure. *Chinese Journal of Clinical Research* 2022: **35(4)**; 472-476.

Luo J, Huang R, Xiao P, Xu A, Dong Z, Zhang L, Wu R, Qiu Y, Zhu L, Zhang R*, et al.* Construction of hub transcription factor-microRNAs-messenger RNA regulatory network in recurrent implantation failure. *Journal of assisted reproduction and genetics* 2024: **41(1)**; 3-13.

Luo JH, Zhu L, Zhou N, Zhang YY, Zhang LR, and Zhang RP. Construction of Circular RNA-MicroRNA-Messenger RNA Regulatory Network of Recurrent Implantation Failure to Explore Its Potential Pathogenesis. *Frontiers in Genetics* 2021: **11**:627459; 14.

Luo L, Li DH, Li XP, Zhang SC, Yan CF, Wu JF, Qi YH, and Zhao J. Polymorphisms in the nuclear factor kappa B gene association with recurrent embryo implantation failure. *Genet Mol Res* 2016: **15(2)**; 1-7.

Lvy Y, Zhang F, Cai Z, Zhong D, and Xing L. Correlation among irrational parenthood cognitions, fertility stress, and social support in patients with repeated implantation failure and the mediating effect of fertility stress: a cross-sectional survey. *Journal of assisted reproduction and genetics* 2024: **41(1)**; 205-212.

Ma N, Li J, Zhang J, Jin Y, Wang J, Qin W, Hang F, and Qin A. Combined oral antibiotics and intrauterine perfusion can improve in vitro fertilization and embryo transfer pregnancy outcomes in patients with chronic endometritis and repeated embryo implantation failure. *BMC Women's Health* 2023: **23(1)**:344.

Madkour A, Bouamoud N, Louanjli N, Kaarouch I, Copin H, Benkhalifa M, and Sefrioui O. Intrauterine insemination of cultured peripheral blood mononuclear cells prior to embryo transfer improves clinical outcome for patients with repeated implantation failures. *Zygote* 2016: **24(1)**; 58-69.

Maftei R, Doroftei B, Popa R, Harabor V, Adam AM, Popa C, Harabor A, Adam G, Nechita A, Vasilache IA*, et al.* The Influence of Maternal KIR Haplotype on the Reproductive Outcomes after Single Embryo Transfer in IVF Cycles in Patients with Recurrent Pregnancy Loss and Implantation Failure-A Single Center Experience. *Journal of Clinical Medicine* 2023: **12(5)**:1905.

Magdi Y, El-Damen A, Fathi AM, Abdelaziz AM, Abd-Elfatah Youssef M, Abd-Allah AA, Ahmed Elawady M, Ahmed Ibrahim M, and Edris Y. Revisiting the management of recurrent implantation failure through freeze-all policy. *Fertility and sterility* 2017: **108(1)**; 72‐77.

Mahdian S, Pirjani R, Favaedi R, Movahedi M, Moini A, and Shahhoseini M. Platelet-activating factor and antiphospholipid antibodies in recurrent implantation failure. *J Reprod Immunol* 2021: **143**; 103251.

Makrigiannakis A, Benkhalifa M, Vrekoussis T, Mahjub S, Kalantaridou SN, and Gurgan T. Repeated implantation failure: a new potential treatment option. *European journal of clinical investigation* 2015: **45(4)**; 380‐384.

Mantzouratou A, Mania A, Fragouli E, Xanthopoulou L, Tashkandi S, Fordham K, Ranieri DM, Doshi A, Nuttall S, Harper JC*, et al.* Variable aneuploidy mechanisms in embryos from couples with poor reproductive histories undergoing preimplantation genetic screening. *Hum Reprod* 2007: **22(7)**; 1844-1853.

Manzur NF, Gluska H, Feferkorn I, Skvirsky S, Ben-Shlomo I, and Wiener-Megnazi Z. Homocysteine serum levels correlate with the number of failed IVF cycles even when within normal range. *Archives of Gynecology and Obstetrics* 2023: **307(6)**; 1975-1982.

Mao X, Zhang J, Chen Q, Kuang Y, and Zhang S. Short-term copper intrauterine device placement improves the implantation and pregnancy rates in women with repeated implantation failure. *Fertil Steril* 2017: **108(1)**; 55-61.e51.

Mariee N, Li TC, and Laird SM. Expression of leukaemia inhibitory factor and interleukin 15 in endometrium of women with recurrent implantation failure after IVF; correlation with the number of endometrial natural killer cells. *Hum Reprod* 2012: **27(7)**; 1946-1954.

Mariee NG, Tuckerman E, Laird S, and Li TC. The correlation of autoantibodies and uNK cells in women with reproductive failure. *J Reprod Immunol* 2012: **95(1-2)**; 59-66.

Marin NSI, Fuente-Munoz EDL, Gil-Laborda R, Villegas A, Alonso-Arenilla B, Cristobal I, Pilar-Suarez L, Jimenez-Huete A, Calvo M, Sarria B*, et al.* Myeloid-derived suppressor cells as a potential biomarker for recurrent pregnancy loss and recurrent implantation failure: Increased levels of MDSCs in recurrent reproductive failure. *American journal of reproductive immunology* 2023: **90(5)**:e13783.

Marron K and Harrity C. Endometrial lymphocyte concentrations in adverse reproductive outcome populations. *J Assist Reprod Genet* 2019: **36(5)**; 837-846.

Marron K and Harrity C. Potential utility of a non-invasive menstrual blood immunophenotype analysis in reproductive medicine. *Reprod Fertil* 2022: **3(4)**; 255-261.

Marron K, Walsh D, and Harrity C. Detailed endometrial immune assessment of both normal and adverse reproductive outcome populations. *J Assist Reprod Genet* 2019: **36(2)**; 199-210.

Martínez-Zamora MA, Creus M, Tassies D, Reverter JC, Civico S, Carmona F, and Balasch J. Reduced plasma fibrinolytic potential in patients with recurrent implantation failure after IVF and embryo transfer. *Hum Reprod* 2011: **26(3)**; 510-516.

Martínez-Zamora MA, Tàssies D, Reverter JC, Creus M, Casals G, Cívico S, Carmona F, and Balasch J. Increased circulating cell-derived microparticle count is associated with recurrent implantation failure after IVF and embryo transfer. *Reprod Biomed Online* 2016: **33(2)**; 168-173.

Martini AE, Jasulaitis S, Fogg LF, Uhler ML, and Hirshfeld Cytron JE. Evaluating the utility of intralipid infusion to improve live birth rates in patients with recurrent pregnancy loss or recurrent implantation failure. *Journal of human reproductive sciences* 2018: **11(3)**; 261-268.

Matteo M, Greco P, Levi Setti PE, Morenghi E, De Rosario F, Massenzio F, Albani E, Totaro P, and Liso A. Preliminary evidence for high anti-PLAC1 antibody levels in infertile patients with repeated unexplained implantation failure. *Placenta* 2013: **34(4)**; 335-339.

Mehrafza M, Kabodmehri R, Nikpouri Z, Pourseify G, Raoufi A, Eftekhari A, Samadnia S, and Hosseini A. Comparing the Impact of Autologous Platelet-rich Plasma and Granulocyte Colony Stimulating Factor on Pregnancy Outcome in Patients with Repeated Implantation Failure. *J Reprod Infertil* 2019: **20(1)**; 35-41.

Mehrafza M, Raoufi A, Hosseinzadeh E, Pourseify GR, Yousefi TZ, Shakery T, and Tamimi A. The Effect of Intrauterine Infusion of Plasma Enriched Platelet on Live Birth Rate in Patients with Implantation Failure: A Retrospective Uncontrolled Study. *Journal of Obstetrics, Gynecology and Cancer Research* 2022: **7(6)**; 518-523.

Mei J, Yan Y, Jiang R, Zhu YC, Ding L, and Sun H. Clinical outcome of intrauterine administration of peripheral mononuclear cells or human chorionic gonadotropin in unexplained implantation failure. *American journal of reproductive immunology* 2022: **87(5)**:e13529.

Mei Y, Wang Y, He L, Zheng J, Lin Y, and Wang F. Performance of preimplantation genetic testing for aneuploidy for patients with unexplained recurrent pregnancy loss and repeated implantation failure. *Heliyon* 2024: **10(11)**; e31983.

Melkozerova OA, Bashmakova NV, Malgina GB, Bragina EE, Michelson AA, and Chistyakova GN. Ultrastructural markers of tissue endometrial receptivity in patients with recurrent implantation failure. *Gynecol Endocrinol* 2019: **35(sup1)**; 45-48.

Meltsov A, Saare M, Teder H, Paluoja P, Arffman RK, Piltonen T, Laudanski P, Wielgos M, Gianaroli L, Koel M*, et al.* Targeted gene expression profiling for accurate endometrial receptivity testing. *Scientific reports* 2023: **13(1)**; 13959.

Mercader A, Simón C, Galán A, Herrer R, Albert C, Remohí J, and Pellicer A. An analysis of spontaneous hatching in a human endometrial epithelial coculture system: Is assisted hatching justified? *Journal of assisted reproduction and genetics* 2001: **18(6)**; 315-319.

Meybodi NF, Eftekhar M, and Gandom B. Intrauterine autologous platelet-rich plasma treatment in women with at least two implantation failures: A retrospective cohort study. *International journal of reproductive biomedicine* 2024: **22(1)**; 9-16.

Mohammadzadeh M, Ghorbian S, and Nouri M. Evaluation of clinical utility of P53 gene variations in repeated implantation failure. *Mol Biol Rep* 2019: **46(3)**; 2885-2891.

Mohtasebi P, Eslami M, Ramezanali F, Borjian Boroujeni P, Rokhsat Talab Z, and Zamanian M. APOE polymorphism status (E4) may help in predicting the risk of recurrent implantation failure. *Int J Gynaecol Obstet* 2021: **158(2)**; 447-452.

Moini A, Zafarani F, Jahangiri N, Jahanian Sadatmahalleh SH, Sadeghi M, Chehrazi M, and Ahmadi F. The Effect of Vaginal Sildenafil on The Outcome of Assisted Reproductive Technology Cycles in Patients with Repeated Implantation Failures: A Randomized Placebo-Controlled Trial. *Int J Fertil Steril* 2020: **13(4)**; 289-295.

Moraru M, Carbone J, Alecsandru D, Castillo-Rama M, García-Segovia A, Gil J, Alonso B, Aguarón A, Ramos-Medina R, Martínez de María J*, et al.* Intravenous immunoglobulin treatment increased live birth rate in a Spanish cohort of women with recurrent reproductive failure and expanded CD56(+) cells. *Am J Reprod Immunol* 2012: **68(1)**; 75-84.

Motak-Pochrzȩst H and Malinowski A. Does autoimmunity play a role in the risk of implantation failures? *Neuroendocrinology Letters* 2017: **38(8)**; 575-578.

Mousavi-Salehi A, Ghafourian M, Amari A, and Zargar M. Evaluation of NKT Cell Percentage and Function and Its Relationship with Serum IFN-γ and Vitamin D Levels in Women with Recurrent Spontaneous Abortion and Recurrent Implantation Failure. *Journal of Obstetrics and Gynecology of India* 2023: **74(5)**; 391-397.

Mrozikiewicz AE, Kurzawinska G, Ozarowski M, Walczak M, Ozegowska K, and Jedrzejczak P. Polymorphic Variants of Genes Encoding Angiogenesis-Related Factors in Infertile Women with Recurrent Implantation Failure. *International Journal of Molecular Sciences* 2023: **24(5)**:4267.

Mutlu AE. Assessment of in vitro fertilization outcomes after hysteroscopic isthmoplasty in recurrent implantation failure. *Journal of Obstetrics and Gynaecology Research* 2022: **48(7)**; 1829-1835.

Mutlu AE. IVF outcomes after T-shaped uterine metroplasty in patients with recurrent implantation failure. *Journal of Gynecology Obstetrics and Human Reproduction* 2022: **51(5)**:102354; 5.

Naghi Jafarabadi M, Hadavi F, Ahmadi M, Masoumi M, and Zabihzadeh S. Intravaginal probiotics before embryo transfer do not improve pregnancy rates in recurrent implantation failure cases: An RCT. *Int J Reprod Biomed* 2024: **22(5)**; 363-374.

Nakagawa K, Horikawa T, Orita Y, Yamashiro E, Watanabe H, Shirai A, Ogata S, Kataoka H, Kuroda K, Takamizawa S*, et al.* Hyaluronan-enriched transfer medium (HETM) can improve the implantation rate in morphologically poor euploid blastocyst transfer. *Archives of Gynecology and Obstetrics* 2023: **308(2)**; 611-619.

Nakagawa K, Juen H, Nishi Y, Sugiyama R, Motoyama H, Kuribayashi Y, Inoue M, Akira S, and Sugiyama R. Advanced scheduling for zygote intrafallopian transfer is possible via the use of a hormone replacement cycle for patients who have experienced repeated implantation failures. *Archives of Gynecology and Obstetrics* 2014: **290(5)**; 1031-1035.

Nakagawa K, Kwak-Kim J, Kuroda K, Sugiyama R, and Yamaguchi K. Immunosuppressive treatment using tacrolimus promotes pregnancy outcome in infertile women with repeated implantation failures. *American journal of reproductive immunology* 2017: **78(3)**:e12682.

Nakagawa K, Kwak-Kim J, Ota K, Kuroda K, Hisano M, Sugiyama R, and Yamaguchi K. Immunosuppression with tacrolimus improved reproductive outcome of women with repeated implantation failure and elevated peripheral blood TH1/TH2 cell ratios. *Am J Reprod Immunol* 2015: **73(4)**; 353-361.

Nami S, Govahi A, Najjar N, Ghasemi S, Rezaei F, Amjadi F, and Taheripak G. Metabolomic profiling of embryo culture media in patients with repeated implantation failure during assisted reproductive technology cycles. *Clin Exp Reprod Med* 2024: **51(3)**; 260-267.

Nazarenko TA, Kalinina EA, Knyazeva EA, Kiselev VI, Smolnikova VY, and Sukhikh GT. The role of abnormal hypermethylation of the HOXA10 and HOXA11 promoters in implantation failures in IVF programs. *Gynecological endocrinology* 2019: **35(sup1)**; 31-34.

Nazari L, Salehpour S, Hoseini S, Zadehmodarres S, and Ajori L. Effects of autologous platelet-rich plasma on implantation and pregnancy in repeated implantation failure: A pilot study. *Int J Reprod Biomed* 2016: **14(10)**; 625-628.

Nazari L, Salehpour S, Hosseini MS, and Hashemi Moghanjoughi P. The effects of autologous platelet-rich plasma in repeated implantation failure: a randomized controlled trial. *Human fertility* 2020: **23(3)**; 209‐213.

Nazari L, Salehpour S, Hosseini S, Sheibani S, and Hosseinirad H. The Effects of Autologous Platelet-Rich Plasma on Pregnancy Outcomes in Repeated Implantation Failure Patients Undergoing Frozen Embryo Transfer: A Randomized Controlled Trial. *Reprod Sci* 2021: **29(3)**; 993-1000.

Negm SM, Kamel RA, and Abuhamila FA. Three-dimensional sonohysterography compared with vaginoscopic hysteroscopy for evaluation of the uterine cavity in patients with recurrent implantation failure in in vitro fertilization cycles. *J Minim Invasive Gynecol* 2012: **19(4)**; 503-508.

Nenonen H, Kondic A, Henic E, and Hjelmer I. Recurrent implantation failure and inflammatory markers in serum and follicle fluid of women undergoing assisted reproduction. *Journal of reproductive immunology* 2024: **162**:104209.

Nguyen TTN, MacDougall M, Kwok YSS, Russell SJ, and Librach CL. Human platelet lysates stimulate in vitro proliferation of human endometrial cells from patients with a history of recurrent implantation failure. *F S Sci* 2022: **3(1)**; 64-73.

Ni T, Zhang Q, Li Y, Huang C, Zhou T, Yan J, and Chen ZJ. CircSTK40 contributes to recurrent implantation failure via modulating the HSP90/AKT/FOXO1 axis. *Mol Ther Nucleic Acids* 2021: **26**; 208-221.

Ni Y, Huang L, Zhang E, Xu L, Tong C, Qian W, Zhang A, and Fang Q. Psychosocial correlates of fertility-related quality of life among infertile women with repeated implantation failure: The mediating role of resilience. *Frontiers in Psychiatry* 2022: **13**:1019922.

Niu Z, Zhou M, Xia L, Zhao S, and Zhang A. Uterine cytokine profiles after low-molecular-weight heparin administration are associated with pregnancy outcomes of patients with repeated implantation failure. *Frontiers in Endocrinology* 2022: **13**:1008923.

Nobijari FF, Arefi SS, Moini A, Taheripanah R, Fazeli E, Kharazi H, Hosseini SZ, Hosseini A, Valojerdi MR, Copin H*, et al.* Endometrium immunomodulation by intrauterine insemination administration of treated peripheral blood mononuclear cell prior frozen/thawed embryos in patients with repeated implantation failure. *Zygote* 2019: **27(4)**; 214-218.

Nosenko OM and Demydchyk RY. Melatonin Exchange in Infertile Women with Repeated Implantation Failures. *Reproductive Endocrinology* 2024: **71**; 94-102.

Noushin MA, Ashraf M, Thunga C, Singh S, Singh S, Basheer R, Ashraf R, and Jayaprakasan K. A comparative evaluation of subendometrial and intrauterine platelet-rich plasma treatment for women with recurrent implantation failure. *F S Sci* 2021: **2(3)**; 295-302.

Nowak I, Bylińska A, Wilczyńska K, Wiśniewski A, Malinowski A, Wilczyński JR, Radwan P, Radwan M, Barcz E, Płoski R*, et al.* The methylenetetrahydrofolate reductase c.c.677 C>T and c.c.1298 A>C polymorphisms in reproductive failures: Experience from an RSA and RIF study on a Polish population. *PLoS One* 2017: **12(10)**; e0186022.

Nowak I, Wilczyńska K, Radwan P, Wiśniewski A, Krasiński R, Radwan M, Wilczyński JR, Malinowski A, and Kuśnierczyk P. Association of Soluble HLA-G Plasma Level and HLA-G Genetic Polymorphism With Pregnancy Outcome of Patients Undergoing in vitro Fertilization Embryo Transfer. *Frontiers in Immunology* 2020: **10**:2982.

Ocal P, Cift T, Bulut B, Balcan E, Cepni I, Aydogan B, and Irez T. Recurrent implantation failure is more frequently seen in female patients with poor prognosis. *Int J Fertil Steril* 2012: **6(2)**; 71-78.

Ohara Y, Matsubayashi H, Suzuki Y, Takaya Y, Yamaguchi K, Doshida M, Takeuchi T, Ishikawa T, Handa M, Miyake T*, et al.* Clinical relevance of a newly developed endometrial receptivity test for patients with recurrent implantation failure in Japan. *Reproductive Medicine and Biology* 2022: **21(1)**:e12444; 9.

Ohgi S, Taga Y, Anakubo H, Kurata Y, Hatakeyama S, and Yanaihara A. Morphologically poor blastocysts could affect the implantation rate of a morphologically good blastocyst during a double-blastocyst transfer for patients who have experienced repeated implantation failures. *Reprod Med Biol* 2018: **17(3)**; 249-254.

Oikonomou G, Vlachadis N, Tsamadias V, Lambrinoudaki I, Deligeoroglou E, Vlahos NF, and Economou E. Human Leukocyte Antigen Alleles Compatibility and Immunophenotypic Profile Associations in Infertile Couples. *Cureus* 2023: **15(3)**; e36584.

Okitsu O, Kiyokawa M, Oda T, Miyake K, Sato Y, and Fujiwara H. Intrauterine administration of autologous peripheral blood mononuclear cells increases clinical pregnancy rates in frozen/thawed embryo transfer cycles of patients with repeated implantation failure. *J Reprod Immunol* 2011: **92(1-2)**; 82-87.

Oliveira JBA, Cavagna M, Petersen CG, Mauri AL, Massaro FC, Silva LFI, Baruffi RLR, and Franco Jr JG. Pregnancy outcomes in women with repeated implantation failures after intracytoplasmic morphologically selected sperm injection (IMSI). *Reproductive Biology and Endocrinology* 2011: **9**; 1-7.

Orazov MR, Radzinskiy VE, Kostin IN, Gagaev CG, and Orekhov RE. Endometrial asynchrony in pathogenesis of implantation impairment in women with infertility associated with endometriosis. *Gynecological endocrinology* 2021: **37(S1)**; 1-3.

Oskouei BS, Shahabi P, Babaei H, and Farshbaf-Khalili A. The effect of luteal phase prolongation with medroxyprogesterone acetate on endometrial thickness and pregnancy rate in women following recurrent implantation failure. *Journal of Research in Clinical Medicine* 2024: **12(1)**:2.

Ozaltin S, Celik HG, Takmaz O, Yagmur E, Ozbasli E, Gungor M, Yeh J, and Bastu E. Is endometrial receptivity assay (era) useful in patients with repeated implantation failure undergoing single, autologous euploid embryo transfer? *Clinical and Experimental Obstetrics and Gynecology* 2022: **49(9)**:4909198.

Ozer G, Akca A, Yuksel B, Duzguner I, Pehlivanli AC, and Kahraman S. Prediction of risk factors for first trimester pregnancy loss in frozen-thawed good-quality embryo transfer cycles using machine learning algorithms. *Journal of assisted reproduction and genetics* 2023: **40(2)**; 279-288.

Ozgu-Erdinc AS, Oskovi-Kaplan ZA, Engin-Ustun Y, Kiyak Caglayan E, Yilmaz S, Yilmaz N, Gocmen AY, Erkaya S, and Delibas N. Recurrent implantation failure is associated with increased levels of fetuin-A/alpha2-Heremans-Schmid-glycoprotein. *Eur J Obstet Gynecol Reprod Biol* 2020: **248**; 1-4.

Pabuccu EG, Yalcin I, Bodur T, Caglar GS, and Pabuccu R. Impact of office hysteroscopy in repeated implantation failure: Experience of a single center. *Journal of the Turkish-German Gynecological Association* 2016: **17(4)**; 197-200.

Pagidas K, Ying Y, and Keefe D. Predictive value of preimplantation genetic diagnosis for aneuploidy screening in repeated IVF-ET cycles among women with recurrent implantation failure. *J Assist Reprod Genet* 2008: **25(2-3)**; 103-106.

Pan D, Yang J, Zhang N, Wang L, Li N, Shi JZ, and Zhou HY. Gonadotropin-releasing hormone agonist downregulation combined with hormone replacement therapy improves the reproductive outcome in frozen-thawed embryo transfer cycles for patients of advanced reproductive age with idiopathic recurrent implantation failure. *Reproductive Biology and Endocrinology* 2022: **20(1)**:26; 8.

Pan X, Qing Q, Zhou J, Sun H, Li L, Cao W, Ye F, Zhu J, Sun Y, and Wang L. Effect of Chinese patent medicine Kunling Pill on endometrial receptivity: A clinical trial, network pharmacology, and animal-based study. *Drug Discoveries and Therapeutics* 2023: **17(4)**; 257-269.

Pantos K, Simopoulou M, Maziotis E, Rapani A, Grigoriadis S, Tsioulou P, Giannelou P, Nitsos N, Tzonis P, Koutsilieris M*, et al.* Introducing intrauterine antibiotic infusion as a novel approach in effectively treating chronic endometritis and restoring reproductive dynamics: a randomized pilot study. *Scientific reports* 2021: **11(1)**:15581; 10.

Pantou A, Mitrakos A, Kokkali G, Petroutsou K, Tounta G, Lazaros L, Dimopoulos A, Sfakianoudis K, Pantos K, Koutsilieris M*, et al.* The impact of preimplantation genetic testing for aneuploidies (PGT-A) on clinical outcomes in high risk patients. *Journal of assisted reproduction and genetics* 2022: **39(6)**; 1341-1349.

Pantou A, Simopoulou M, Sfakianoudis K, Giannelou P, Rapani A, Maziotis E, Grigoriadis S, Tsioulou P, Syrkos S, Souretis K*, et al.* The Role of Laparoscopic Investigation in Enabling Natural Conception and Avoiding in vitro Fertilization Overuse for Infertile Patients of Unidentified Aetiology and Recurrent Implantation Failure Following in vitro Fertilization. *J Clin Med* 2019: **8(4)**:548.

Papuchova H, Saxtorph MH, Hallager T, Jepsen IE, Eriksen JO, Persson G, Funck T, Weisdorf I, Macklon N, Larsen LG*, et al.* Opposing impacts of HLA-G haplotypes PROMO-G010104-UTR-3 and PROMO-G010101b/c-UTR-4 on risk of recurrent implantation failure. *Reproductive biomedicine online* 2023: **47(3)**:103225.

Papúchová H, Saxtorph MH, Hallager T, Jepsen IE, Eriksen JO, Persson G, Funck T, Weisdorf I, Macklon NS, Larsen LG*, et al.* Endometrial HLA-F expression is influenced by genotypes and correlates differently with immune cell infiltration in IVF and recurrent implantation failure patients. *Hum Reprod* 2022: **37(8)**; 1816-1834.

Park HS, Kim JO, An HJ, Ryu CS, Ko EJ, Kim YR, Ahn EH, Lee WS, Kim JH, and Kim NK. Genetic polymorphisms of the cobalamin transport system are associated with idiopathic recurrent implantation failure. *J Assist Reprod Genet* 2019: **36(7)**; 1513-1522.

Parvanov D, Ganeva R, Arsov K, Decheva I, Handzhiyska M, Ruseva M, Vidolova N, Scarpellini F, Metodiev D, and Stamenov G. Association between endometrial senescent cells and immune cells in women with repeated implantation failure. *Journal of assisted reproduction and genetics* 2023: **40(7)**; 1631-1638.

Parvanov D, Ganeva R, Vidolova N, and Stamenov G. Decreased number of p16-positive senescent cells in human endometrium as a marker of miscarriage. *Journal of assisted reproduction and genetics* 2021: **38(8)**; 2087-2095.

Patel J, Patel A, Banker J, Shah S, and Banker M. Personalized embryo transfer helps in improving In vitro fertilization/ICSI outcomes in patients with recurrent implantation failure. *Journal of human reproductive sciences* 2019: **12(1)**; 59-66.

Patel N, Patel N, Pal S, Nathani N, Pandit R, Patel M, Patel N, Joshi C, and Parekh B. Distinct gut and vaginal microbiota profile in women with recurrent implantation failure and unexplained infertility. *BMC Womens Health* 2022: **22(1)**:113; 15.

Pathare ADS and Hinduja I. Aberrant DNA methylation profiling affecting the endometrial receptivity in recurrent implantation failure patients undergoing in vitro fertilization. *Am J Reprod Immunol* 2020: **83(1)**; e13196.

Pathare ADS and Hinduja I. Endometrial Expression of Cell Adhesion Genes in Recurrent Implantation Failure Patients in Ongoing IVF Cycle. *Reproductive Sciences* 2022: **29(2)**; 513-523.

Pecorino B, Scibilia G, Rapisarda F, Borzi P, Vento ME, Teodoro MC, and Scollo P. Evaluation of implantation and clinical pregnancy rates after endometrial scratching in women with recurrent implantation failure. *Italian journal of gynaecology and obstetrics* 2018: **30(2)**; 39‐44.

Pehlivan T, Rubio C, Rodrigo L, Romero J, Remohi J, Simón C, and Pellicer A. Impact of preimplantation genetic diagnosis on IVF outcome in implantation failure patients. *Reproductive biomedicine online* 2003: **6(2)**; 232-237.

Peivandi S, Mortazavi L, Gordani N, Zamaniyan M, Asgarian-Omran H, Ajami A, and Khademloo M. Effect of Intralipid Infusion on Pregnancy Outcome in Infertile Women with History of Implantation Failure: A Single Blind Randomized Clinical Trial. [Persian]. *Journal of Mazandaran University of Medical Sciences* 2022: **32(208)**; 16-26.

Pérez-Debén S, Bellver J, Alamá P, Salsano S, Quiñonero A, Sebastian-Leon P, Díaz-Gimeno P, and Domínguez F. iTRAQ comparison of proteomic profiles of endometrial receptivity. *J Proteomics* 2019: **203**; 103381.

Petersen CG, Mauri AL, Baruffi RL, Oliveira JB, Massaro FC, Elder K, and Franco JG. Implantation failures: success of assisted hatching with quarter-laser zona thinning. *Reproductive biomedicine online* 2005: **10(2)**; 224‐229.

Piekarska K, Dratwa M, Radwan P, Radwan M, Bogunia-Kubik K, and Nowak I. Pro- and anti-inflammatory cytokines and growth factors in patients undergoing in vitro fertilization procedure treated with prednisone. *Frontiers in Immunology* 2023: **14(no pagination)**:1250488.

Piekarska K, Radwan P, Tarnowska A, Radwan M, Wilczynski JR, Malinowski A, and Nowak I. ERAP/HLA-C and KIR Genetic Profile in Couples with Recurrent Implantation Failure. *International Journal of Molecular Sciences* 2022: **23(20)**:12518.

Piekarska K, Radwan P, Tarnowska A, Wiśniewski A, Radwan M, Wilczyński JR, Malinowski A, and Nowak I. ERAP, KIR, and HLA-C Profile in Recurrent Implantation Failure. *Front Immunol* 2021: **12**; 755624.

Ping P, Liu Y, Zheng Z, Ma Y, Dong F, and Chen X. Association of embryo aneuploidy and sperm DNA damage in unexplained recurrent implantation failure patients under NGS-based PGT-A cycles. *Archives of Gynecology and Obstetrics* 2023: **308(3)**; 997-1005.

Pirtea P, De Ziegler D, Tao X, Sun L, Zhan Y, Ayoubi JM, Seli E, Franasiak JM, and Scott RT, Jr. Rate of true recurrent implantation failure is low: results of three successive frozen euploid single embryo transfers. *Fertil Steril* 2021: **115(1)**; 45-53.

Platteau P, Staessen C, Michiels A, Van Steirteghem A, Liebaers I, and Devroey P. Which patients with recurrent implantation failure after IVF benefit from PGD for aneuploidy screening? *Reprod Biomed Online* 2006: **12(3)**; 334-339.

Pontré JC, Ryan JP, Tan A, and Hart RJ. The interval transfer of a frozen-thawed embryo is more successful than a fresh embryo transfer for women undergoing IVF with recurrent implantation failure after cleavage stage embryo biopsy. *Aust N Z J Obstet Gynaecol* 2019: **59(1)**; 134-139.

Pourmoghadam Z, Soltani-Zangbar MS, Sheikhansari G, Azizi R, Eghbal-Fard S, Mohammadi H, Siahmansouri H, Aghebati-Maleki L, Danaii S, Mehdizadeh A*, et al.* Intrauterine administration of autologous hCG- activated peripheral blood mononuclear cells improves pregnancy outcomes in patients with recurrent implantation failure; A double-blind, randomized control trial study. *Journal of reproductive immunology* 2020: **142**; 103182.

Primi MP, Senn A, Montag M, Van der Ven H, Mandelbaum J, Veiga A, Barri P, and Germond M. A European multicentre prospective randomized study to assess the use of assisted hatching with a diode laser and the benefit of an immunosuppressive/antibiotic treatment in different patient populations. *Human reproduction (Oxford, England)* 2004: **19(10)**; 2325‐2333.

Qi R, Guan R, Cai S, Xu M, Yang WJ, and Wang CC. Comprehensive molecular expression profiling of SARS-CoV-associated factors in the endometrium across the menstrual cycle and elevated susceptibility in women with recurrent pregnancy loss. *Frontiers in Genetics* 2023: **14**:1246725.

Qi R, Zhang T, Zhang Y, Chung JPW, Yang WJ, and Wang CC. Association of angiotensin II and receptors in peri-implantation endometrium with microvessel density and pregnancy outcomes of women with recurrent implantation failure after embryo transfer. *Frontiers in Endocrinology* 2023: **14**:1206326.

Qiu Q, Li Y, Fong SW, Lee KC, Chen ACH, Ruan H, Lee KF, Li RHW, Ng EHY, Yeung WSB*, et al.* Endometrial stromal cells from women with repeated implantation failure display impaired invasion towards trophoblastic spheroids. *Reproduction* 2023: **165(3)**; 335-346.

Quenby S, Anim-Somuah M, Kalumbi C, Farquharson R, and Aplin JD. Different types of recurrent miscarriage are associated with varying patterns of adhesion molecule expression in endometriu. *Reproductive biomedicine online* 2007: **14(2)**:2542; 224-234.

Quenby S, Nik H, Innes B, Lash G, Turner M, Drury J, and Bulmer J. Uterine natural killer cells and angiogenesis in recurrent reproductive failure. *Human Reproduction* 2009: **24(1)**; 45-54.

Quintero-Ronderos P, Jiménez KM, Esteban-Pérez C, Ojeda DA, Bello S, Fonseca DJ, Coronel MA, Moreno-Ortiz H, Sierra-Díaz DC, Lucena E*, et al.* FOXD1 mutations are related to repeated implantation failure, intra-uterine growth restriction and preeclampsia. *Mol Med* 2019: **25(1)**; 37.

Rageh K. Prp in recurrent implantation failure, hope or hype? *Arab Gulf Journal of Scientific Research* 2020: **38(special issue)**; 24.

Rahman A, Francomano D, Sagnella F, Lisi F, and Manna C. The effect on clinical results of adding recombinant LH in late phase of ovarian stimulation of patients with repeated implantation failure: a pilot study. *European review for medical and pharmacological sciences* 2017: **21(23)**; 5485‐5490.

Rajaei S, Mirahmadian M, Jeddi-Tehrani M, Tavakoli M, Zonoobi M, Dabbagh A, and Zarnani AH. Effect of 1,25(OH)(2) vitamin D-3 on cytokine production by endometrial cells of women with repeated implantation failure. *Gynecological endocrinology* 2012: **28(11)**; 906-911.

Rajaei S, Zarnani AH, Jeddi-Tehrani M, Tavakoli M, Mohammadzadeh A, Dabbagh A, and Mirahmadian M. Cytokine profile in the endometrium of normal fertile and women with repeated implantation failure. *Iran J Immunol* 2011: **8(4)**; 201-208.

Ramos-Medina R, García-Segovia A, Gil J, Carbone J, Aguarón de la Cruz A, Seyfferth A, Alonso B, Alonso J, León JA, Alecsandru D*, et al.* Experience in IVIg therapy for selected women with recurrent reproductive failure and NK cell expansion. *Am J Reprod Immunol* 2014: **71(5)**; 458-466.

Raziel A, Friedler S, Schachter M, Kasterstein E, Strassburger D, and Ron-El R. Increased frequency of female partner chromosomal abnormalities in patients with high-order implantation failure after in vitro fertilization. *Fertil Steril* 2002: **78(3)**; 515-519.

Reilly SJ, Glanville EJ, Dhorepatil B, Prentice LR, Mol BW, and Johnson NP. The IVF-LUBE trial - a randomized trial to assess Lipiodol® uterine bathing effect in women with endometriosis or repeat implantation failure undergoing IVF. *Reproductive biomedicine online* 2019: **38(3)**; 380‐386.

Revel A, Achache H, Stevens J, Smith Y, and Reich R. MicroRNAs are associated with human embryo implantation defects. *Hum Reprod* 2011: **26(10)**; 2830-2840.

Rigos I, Athanasiou V, Vlahos N, Papantoniou N, Profer D, and Siristatidis C. The Addition of Endometrial Injury to Freeze-All Strategy in Women with Repeated Implantation Failures. *J Clin Med* 2021: **10(10)**:2162.

Robeva R, Marinova E, Andonova S, Nikolaev G, Savov A, Tanev D, Nikolov G, and Konakchieva R. Melatonin Receptor 1B and Corticosteroid Receptor Polymorphisms in Infertile Women with Implantation Failure and Miscarriages. *Frontiers in bioscience (Landmark edition)* 2023: **28(6)**; 122.

Rockenbach MK, Fraga LR, Kowalski TW, and Sanseverino MTV. Revealing the expression profile of genes that encode the Subcortical Maternal Complex in human reproductive failures. *Genetics and Molecular Biology* 2023: **46(3 Supplement 1)**:e20230141.

Rogenhofer N, Markoff A, Ennerst X, Bogdanova N, and Thaler C. Maternal and paternal carriage of the annexin A5 M2 haplotype: a possible risk factor for recurrent implantation failure (RIF). *J Assist Reprod Genet* 2021: **38(1)**; 235-242.

RoyChoudhury S, Singh A, Gupta NJ, Srivastava S, Joshi MV, Chakravarty B, and Chaudhury K. Repeated implantation failure versus repeated implantation success: discrimination at a metabolomic level. *Hum Reprod* 2016: **31(6)**; 1265-1274.

Rubio C, Gil-Salom M, Simon C, Rodrigo L, Minguez Y, Remohi J, and Pellicer A. Incidence of sperm chromosomal abnormalities in a risk population: relationship with sperm quality and ICSI outcome. *Human Reproduction* 2001: **16(10)**; 2084-2092.

Rufas-Sapir O, Stein A, Orvieto R, Avrech OM, Kotler N, Pinkas H, Bar J, and Fisch B. Is assisted hatching beneficial in patients with recurrent implantation failures? *Clinical and experimental obstetrics & gynecology* 2004: **31(2)**; 110‐112.

Ruiz-Alonso M, Blesa D, Díaz-Gimeno P, Gómez E, Fernández-Sánchez M, Carranza F, Carrera J, Vilella F, Pellicer A, and Simón C. The endometrial receptivity array for diagnosis and personalized embryo transfer as a treatment for patients with repeated implantation failure. *Fertility and sterility* 2013: **100(3)**; 818‐824.

Russell SJ, Kwok YSS, Nguyen TTN, and Librach C. Autologous platelet-rich plasma improves the endometrial thickness and live birth rate in patients with recurrent implantation failure and thin endometrium. *J Assist Reprod Genet* 2022: **39(6)**; 1305-1312.

Ryu CS, Kim YR, Kim JO, An HJ, Cho SH, Ahn EH, Kim JH, Lee WS, and Kim NK. The association of AGO1 (rs595961G>A, rs636832A>G) and AGO2 (rs11996715C>A, rs2292779C>G, rs4961280C>A) polymorphisms and risk of recurrent implantation failure. *Biosci Rep* 2019: **39(11)**:BSR20190342.

Sacks G and Zhang J. Prednisolone and enoxaparin (clexane) therapy (‘the Bondi protocol’) for repeated IVF failure. *American journal of reproductive immunology* 2022: **88(5)**:e13616.

Sadeghpour S, Berenji MG, Nazarian H, Ghasemnejad T, Nematollahi MH, Abroon S, Paktinat S, Khoei HH, Berenji HG, and Novin MG. Effects of treatment with hydroxychloroquine on the modulation of Th17/Treg ratio and pregnancy outcomes in women with recurrent implantation failure: clinical trial. *Immunopharmacology and Immunotoxicology* 2020: **42(6)**; 632-642.

Safdarian L, Aleyasin A, Aghahoseini M, Lak P, Mosa SH, Sarvi F, Mandavi A, Najafian A, Falahi P, and Khazaei S. Efficacy of the Intrauterine Infusion of Platelet-Rich Plasma on Pregnancy Outcomes in Patients With Repeated Implantation Failure: A Randomized Control Trial. *International Journal of Womens Health and Reproduction Sciences* 2022: **10(1)**; 38-44.

Safdarian L, Najmi Z, Aleyasin A, Aghahosseini M, Rashidi M, and Asadollah S. Recurrent IVF failure and hereditary thrombophilia. *Iran J Reprod Med* 2014: **12(7)**; 467-470.

Safran A, Lewin A, Simon A, Holzer H, and Laufer N. Blastocyst culture in evaluating embryos of reduced quality. *Reproductive Technologies* 2000: **10(3)**; 154-157.

Sak ME, Gul T, Evsen MS, Soydinc HE, Sak S, Ozler A, and Alabalik U. Fibroblast growth factor-1 expression in the endometrium of patients with repeated implantation failure after in vitro fertilization. *Eur Rev Med Pharmacol Sci* 2013: **17(3)**; 398-402.

Salazar LA, Inostroza M, Jara C, Vega F, García R, Ciuffardi I, and Guzmán N. Association of -765G>C polymorphism of the COX-2 gene with recurrent embryo implantation failure in Southern Chilean women. *Clin Chim Acta* 2010: **411(21-22)**; 1822-1824.

Salazar MD, Wang WJ, Skariah A, He QH, Field K, Nixon M, Reed R, Dambaeva S, Beaman K, Gilman-Sachs A*, et al.* Post-hoc evaluation of peripheral blood natural killer cell cytotoxicity in predicting the risk of recurrent pregnancy losses and repeated implantation failures. *Journal of reproductive immunology* 2022: **150**:103487; 7.

Salehi AM, Ghafourian M, Amari A, and Zargar M. Evaluation of CD3+ T Cell Percentage, Function and its Relationship with Serum Vitamin D Levels in Women with Recurrent Spontaneous Abortion and Recurrent Implantation Failure. *Iranian Journal of Immunology* 2022: **19(4)**; 369-377.

Salehpour S, Hosseini S, Razghandi Z, Hosseinirad H, and Ziaee H. Comparing the effect of sequential embryo transfer versus double blastocyst embryo transfer on pregnancy outcomes in intracytoplasmic sperm injection (ICSI) cycles in patients with repeated implantation failure: A randomized controlled trial. *Taiwanese Journal of Obstetrics and Gynecology* 2023: **62(2)**; 264-269.

Salehpour S, Zamaniyan M, Saharkhiz N, Modares SZ, Hosieni S, Seif S, Malih N, Rezapoor P, and Sohrabi MR. Does intrauterine saline infusion by intrauterine insemination (IUI) catheter as endometrial injury during IVF cycles improve pregnancy outcomes among patients with recurrent implantation failure?: An RCT. *International journal of reproductive biomedicine* 2016: **14(9)**; 583-588.

Santhosh A, S.P SP, Gunasheela D, Nayak R, and Shetty S. HLA allele frequency of HLA-A, -B, -C, -DRB1 and -DQB1 in Indian recurrent implantation failure and recurrent pregnancy loss couples - A retrospective study. *Journal of reproductive immunology* 2024: **163**:104225.

Santiago KY, Porchia LM, and López-Bayghen E. Endometrial preparation with etanercept increased embryo implantation and live birth rates in women suffering from recurrent implantation failure during IVF. *Reprod Biol* 2021: **21(1)**; 100480.

Santillán I, Lozano I, Illán J, Verdú V, Coca S, Bajo-Arenas JM, and Martinez F. Where and when should natural killer cells be tested in women with repeated implantation failure? *J Reprod Immunol* 2015: **108**; 142-148.

Sato T, Sugiura-Ogasawara M, Ozawa F, Yamamoto T, Kato T, Kurahashi H, Kuroda T, Aoyama N, Kato K, Kobayashi R*, et al.* Preimplantation genetic testing for aneuploidy: a comparison of live birth rates in patients with recurrent pregnancy loss due to embryonic aneuploidy or recurrent implantation failure. *Hum Reprod* 2019: **34(12)**; 2340-2348.

Sauer R, Roussev R, Jeyendran RS, and Coulam CB. Prevalence of antiphospholipid antibodies among women experiencing unexplained infertility and recurrent implantation failure. *Fertil Steril* 2010: **93(7)**; 2441-2443.

Saxtorph MH, Hallager T, Persson G, Petersen KB, Eriksen JO, Larsen LG, Hviid TV, and Macklon N. Assessing endometrial receptivity after recurrent implantation failure: a prospective controlled cohort study. *Reprod Biomed Online* 2020: **41(6)**; 998-1006.

Scarpellini F and Sbracia M. Modification of peripheric Treg and CD56(bright)NK levels in RIF women after egg donation, treated with GM-CSF or placebo. *J Reprod Immunol* 2023: **158**; 103983.

Schachter-Safrai N, Karavani G, Reuveni-Salzman A, Gil M, and Ben-Meir A. Which semen analysis correlates with favorable Intracytoplasmic morphologically selected sperm injection (IMSI) outcomes? *European Journal of Obstetrics and Gynecology and Reproductive Biology* 2019: **234**; 85-88.

Sebastian-Leon P, Garrido N, Remohí J, Pellicer A, and Diaz-Gimeno P. Asynchronous and pathological windows of implantation: two causes of recurrent implantation failure. *Hum Reprod* 2018: **33(4)**; 626-635.

Seles L, Zaha IA, Luncan M, Bodog A, Sachelarie L, Sandor M, Macovei IC, Bimbo-Szuhai E, and Huniadi A. Immunomodulatory Treatment Impact on IVF Outcomes in KIR AA Genotype: Personalized Fertility Insights. *Medicina (Kaunas)* 2024: **60(6)**:948.

Seval MM, Şükür YE, Özmen B, Kan Ö, Sönmezer M, Berker B, and Atabekoğlu C. Does adding endometrial scratching to diagnostic hysteroscopy improve pregnancy rates in women with recurrent in-vitro fertilization failure? *Gynecol Endocrinol* 2016: **32(12)**; 957-960.

Shah J, Gangadharan A, and Shah V. Effect of intrauterine instillation of granulocyte colonystimulating factor on endometrial thickness and clinical pregnancy rate in women undergoing in vitro fertilization cycles: An observational cohort study. *International Journal of Infertility and Fetal Medicine* 2014: **5(3)**; 100-106.

Shahrokh Tehraninejad E, Azimi Nekoo E, Ghaffari F, Hafezi M, Karimian L, and Arabipoor A. Zygote intrafallopian tube transfer versus intrauterine cleavage or blastocyst stage transfer after intracytoplasmic sperm injection cycles in patients with repeated implantation failure: A prospective follow-up study. *J Obstet Gynaecol Res* 2015: **41(11)**; 1779-1784.

Shahrokh-Tehraninejad E, Dashti M, Hossein-Rashidi B, Azimi-Nekoo E, Haghollahi F, and Kalantari V. A randomized trial to evaluate the effect of local endometrial injury on the clinical pregnancy rate of frozen embryo transfer cycles in patients with repeated implantation failure. *Journal of family and reproductive health* 2016: **10(3)**; 108‐114.

Shang J, Cheng YF, Li M, Wang H, Zhang JN, Guo XM, Cao DD, and Yao YQ. Identification of key endometrial microRNAs and their target genes associated with pathogenesis of recurrent implantation failure by integrated bioinformatics analysis. *Frontiers in Genetics* 2022: **13**:919301.

Sharif KW and Ghunaim S. Management of 273 cases of recurrent implantation failure: results of a combined evidence-based protocol. *Reprod Biomed Online* 2010: **21(3)**; 373-380.

Sheikhansari G, Soltani-Zangbar MS, Pourmoghadam Z, Kamrani A, Azizi R, Aghebati-Maleki L, Danaii S, Koushaeian L, Hojat-Farsangi M, and Yousefi M. Oxidative stress, inflammatory settings, and microRNA regulation in the recurrent implantation failure patients with metabolic syndrome. *Am J Reprod Immunol* 2019: **82(4)**; e13170.

Shen L, Zhang YR, Chen WF, and Yin XH. The Application of Artificial Intelligence in Predicting Embryo Transfer Outcome of Recurrent Implantation Failure. *Frontiers in Physiology* 2022: **13**:885661; 12.

Shi C, Han HJ, Fan LJ, Guan J, Zheng XB, Chen X, Liang R, Zhang XW, Sun KK, Cui QH*, et al.* Diverse endometrial mRNA signatures during the window of implantation in patients with repeated implantation failure. *Hum Fertil (Camb)* 2018: **21(3)**; 183-194.

Shi C, Shen H, Fan LJ, Guan J, Zheng XB, Chen X, Liang R, Zhang XW, Cui QH, Sun KK*, et al.* Endometrial MicroRNA Signature during the Window of Implantation Changed in Patients with Repeated Implantation Failure. *Chin Med J (Engl)* 2017: **130(5)**; 566-573.

Shi MF, Zhou T, Wang Y, Chen GH, Zhu M, Ou J, Du H, Yan TF, Song D, Zheng JF*, et al.* Revised super-long down-regulation protocol improves the outcome of infertile patients with repeated implantation failure in IVF/ICSI-ET. *Journal of Reproduction and Contraception* 2014: **25(2)**; 89-96.

Shi X, Tang Y, Liu C, Li W, Lin H, Mao W, Huang M, Chu Q, Wang L, Quan S*, et al.* Effects of NGS-based PGT-a for idiopathic recurrent pregnancy loss and implantation failure: a retrospective cohort study. *Systems Biology in Reproductive Medicine* 2023: **69(5)**; 354-365.

Shim SH, Kim JO, Jeon YJ, An HJ, Lee HA, Kim JH, Ahn EH, Lee WS, and Kim NK. Association between vascular endothelial growth factor promoter polymorphisms and the risk of recurrent implantation failure. *Exp Ther Med* 2018: **15(2)**; 2109-2119.

Shirmohamadi M, Mashayekhy M, Alipourfard I, Fazeli J, and Ghasemi N. Effect of heparin on recurrent IVF-ET failure patients. *Asian Pacific Journal of Reproduction* 2023: **12(2)**; 64-70.

Shiva M, Ahmadi F, Arabipoor A, Oromiehchi M, and Chehrazi M. Accuracy of Two-Dimensional Transvaginal Sonography and Office Hysteroscopy for Detection of Uterine Abnormalities in Patients with Repeated Implantation Failures or Recurrent Pregnancy Loss. *Int J Fertil Steril* 2018: **11(4)**; 287-292.

Shohayeb A and El-Khayat W. Does a single endometrial biopsy regimen (S-EBR) improve ICSI outcome in patients with repeated implantation failure? A randomised controlled trial. *European journal of obstetrics, gynecology, and reproductive biology* 2012: **164(2)**; 176‐179.

Shu M, Yao S, Zhong W, Song C, Chen F, and Shang W. Personalized embryo transfer based on RNA sequencing endometrial receptivity test in repeated implantation failure patients: artificial cycle versus natural cycle. *Gynecological endocrinology* 2023: **39(1)**:2181639.

Shu MM, Zhou YJ, Liang H, Han HM, Zhong W, Yao S, Ruan ZL, Yu D, and Shang W. Reproductive Outcomes of in Vitro Fertilization and Embryo Transfer in Women with Unexplained Repeated Implantation Failure are Significantly Improved with Intravenous Immunoglobulins. *Clinical and experimental obstetrics & gynecology* 2023: **50(7)**:137; 8.

Shuai Z, Li X, Tang X, Lian F, and Sun Z. Transcutaneous electrical acupuncture point stimulation improves pregnancy outcomes in patients with recurrent implantation failure undergoing in vitro fertilisation and embryo transfer: a prospective, randomised trial. *Acupuncture in medicine* 2019: **37(1)**; 33‐39.

Singh N, Bhat MA, and Ghosh D. Prediction of pregnancy outcome of IVF-ET cycles following endometrial injury in women with previously failed implantation based on endometrial transcriptomics: A preliminary report. *Journal of Reproductive Health and Medicine* 2016: **2(2)**; 83-92.

Singh N, Dogra Y, Kumar P, Mathur S, Sharma A, and Patel G. Establishment of Cut-off Values for Uterine and Peripheral Blood Natural Killer Cells During the Peri-implantation Period in Fertile Controls and Women with Unexplained Recurrent Implantation Failure. *Journal of reproduction and infertility* 2023: **24(4)**; 248-256.

Singh PN, Pathak AM, Singh P, and Desai M. Selecting euploid embryos for transfer by preimplantation genetic testing with the help of next-generation sequencing in poor prognosis patients: A retrospective cohort analysis. *Journal of human reproductive sciences* 2022: **15(2)**; 157-162.

Siristatidis C, Dafopoulos K, El-Khayat W, Salamalekis G, Anifandis G, Vrantza T, Elsadek M, and Papantoniou N. Administration of prednisolone and low molecular weight heparin in patients with repeated implantation failures: a cohort study. *Gynecol Endocrinol* 2018: **34(2)**; 136-139.

Siristatidis C, Kreatsa M, Koutlaki N, Galazios G, Pergialiotis V, and Papantoniou N. Endometrial injury for RIF patients undergoing IVF/ICSI: a prospective nonrandomized controlled trial. *Gynecological endocrinology* 2017: **33(4)**; 297‐300.

Soczewski E, Murrieta-Coxca JM, Miranda L, Fuentes-Zacarias P, Gutierrez-Samudio R, Grasso E, Marti M, PerezLeiros C, Morales-Prieto D, Markert UR*, et al.* miRNAs associated with endoplasmic reticulum stress and unfolded protein response during decidualization. *Reproductive biomedicine online* 2023: **47(5)**:103289.

Somigliana E, Vigano P, Busnelli A, Paffoni A, Vegetti W, and Vercellini P. Repeated implantation failure at the crossroad between statistics, clinics and over-diagnosis. *Reprod Biomed Online* 2018: **36(1)**; 32-38.

Stamenov G, Penkova K, Chaushev T, Persenska S, Dzhambazov B, Iliev I, and Baltadjieva D. Endometrial NK cell subpopulations CD16- CD56bright and CD16- CD56dim in women with recurrent implantation failure. *Biotechnology and Biotechnological Equipment* 2013: **27(5)**; 4123-4126.

Stamenov GS, Parvanov DA, and Chaushev TA. Mixed double-embryo transfer: A promising approach for patients with repeated implantation failure. *Clin Exp Reprod Med* 2017: **44(2)**; 105-110.

Stein A, Rufas O, Amit S, Avrech O, Pinkas H, Ovadia J, and Fisch B. Assisted hatching by partial zona dissection of human pre-embryos in patients with recurrent implantation failure after in vitro fertilization. *Fertility and sterility* 1995: **63(4)**; 838-841.

Steiner N, Shrem G, Tannus S, Dahan SY, Balayla J, Volodarsky-Perel A, Tan SL, and Dahan MH. Effect of GnRH agonist and letrozole treatment in women with recurrent implantation failure. *Fertil Steril* 2019: **112(1)**; 98-104.

Stevens Brentjens L, Habets D, Den Hartog J, Al-Nasiry S, Wieten L, Morré S, Van Montfoort A, Romano A, and van Golde R. Endometrial factors in the implantation failure spectrum: protocol of a MUltidisciplinary observational cohort study in women with Repeated Implantation failure and recurrent Miscarriage (MURIM Study). *BMJ open* 2022: **12(6)**; e056714.

Stocker L, Cagampang F, and Cheong Y. Identifying stably expressed housekeeping genes in the endometrium of fertile women, women with recurrent implantation failure and recurrent miscarriages. *Sci Rep* 2017: **7(1)**; 14857.

Stocker LJ, Cagampang FR, Lu S, Ladyman T, and Cheong YC. Is sleep deficit associated with infertility and recurrent pregnancy losses? Results from a prospective cohort study. *Acta Obstet Gynecol Scand* 2021: **100(2)**; 302-313.

Sudoma I, Goncharova Y, Dons'koy B, and Mykytenko D. Immune phenotype of the endometrium in patients with recurrent implantation failures after the transfer of genetically tested embryos in assisted reproductive technology programs. *Journal of reproductive immunology* 2023: **157**:103943.

Sudoma IO, Goncharova YO, and Dons’Koy BV. Immunomodulatory effect of granulocyte colony-stimulating factor in repeated implantation failures in embryo transfer programs. *Reproductive Health of Woman* 2023: **2023(1)**; 73-81.

Sui YL, Li L, and Sun XX. Integrated bioinformatics analysis to identify key genes and pathways involved in the endometria of patients with recurrent implantation failure undergoing in vitro fertilization and embryo transfer. *Reproductive and Developmental Medicine* 2023: **7(4)**; 230-237.

Sulima AN, Voronaya VV, Davydova AA, Rybalka AN, and Dizha MA. The predictively favorable factors of clinical pregnancy at frozen embryo transfer protocols in patients with repeated implantation failures. *New Armenian Medical Journal* 2019: **13(2)**; 87-95.

Sun Y, Cui L, Lu Y, Tan J, Dong X, Ni T, Yan J, Guan Y, Hao G, Liu JY*, et al.* Prednisone vs Placebo and Live Birth in Patients With Recurrent Implantation Failure Undergoing In Vitro Fertilization: A Randomized Clinical Trial. *Jama* 2023: **329(17)**; 1460-1468.

Sun Y, Zhang Y, Ma X, Jia W, and Su Y. Determining Diagnostic Criteria of Unexplained Recurrent Implantation Failure: A Retrospective Study of Two vs Three or More Implantation Failure. *Front Endocrinol (Lausanne)* 2021: **12**; 619437.

Sung N, Khan SA, Yiu ME, Jubiz G, Salazar MD, Skariah A, Dambaeva S, and Kwak-Kim J. Reproductive outcomes of women with recurrent pregnancy losses and repeated implantation failures are significantly improved with immunomodulatory treatment. *J Reprod Immunol* 2021: **148**; 103369.

Tada Y, Kitaya K, Amano N, Kobatake M, Hayashi T, Taguchi S, Funabiki M, and Nakamura Y. A pilot survey on obstetric complications in pregnant women with a history of repeated embryo implantation failure and those undergoing single local endometrial injury. *Clin Exp Obstet Gynecol* 2015: **42(2)**; 176-178.

Taheripanah R, Zamaniyan M, Akhoondzadeh S, Taheripanah A, and Malih N. Uterine and serum glycodelin concentration in recurrent implantation failure versus normal fertile women on implantation window. *International Journal of Women's Health and Reproduction Sciences* 2017: **5(2)**; 103-106.

Takimoto K, Yamada H, Shimada S, Fukushi Y, and Wada S. Chronic Endometritis and Uterine Endometrium Microbiota in Recurrent Implantation Failure and Recurrent Pregnancy Loss. *Biomedicines* 2023: **11(9)**:2391.

Tan J, Kan A, Hitkari J, Taylor B, Tallon N, Warraich G, Yuzpe A, and Nakhuda G. The role of the endometrial receptivity array (ERA) in patients who have failed euploid embryo transfers. *J Assist Reprod Genet* 2018: **35(4)**; 683-692.

Tang CL, Li QY, Chen FL, Cai CT, Dong YY, Wu YY, Yang JZ, Zhao M, Chi FL, Hong L*, et al.* A randomized double blind comparison of atosiban in patients with recurrent implantation failure undergoing IVF treatment. *Reproductive Biology and Endocrinology* 2022: **20(1)**:124.

Tangal S, Caglar GS, Papuccu EG, Keskin M, and Haliloglu AH. Intracytoplasmic sperm injection outcomes after anti-oxidant treatment in repeated implantation failure. *European Research Journal* 2019: **5(5)**; 868-872.

Tapia A, Gangi LM, Zegers-Hochschild F, Balmaceda J, Pommer R, Trejo L, Pacheco IM, Salvatierra AM, Henríquez S, Quezada M*, et al.* Differences in the endometrial transcript profile during the receptive period between women who were refractory to implantation and those who achieved pregnancy. *Hum Reprod* 2008: **23(2)**; 340-351.

Tapia-Pizarro A, Figueroa P, Brito J, Marín JC, Munroe DJ, and Croxatto HB. Endometrial gene expression reveals compromised progesterone signaling in women refractory to embryo implantation. *Reprod Biol Endocrinol* 2014: **12**; 92.

Tapilskaya NI, Dzhemlikhanova L, Krikheli IO, Mekina ID, Lesik EA, Komarova EM, Gzgzyan AM, and Kogan I. Combined use of granulocyte colony stimulating factor in repeated implantation failure. *Russian Journal of Human Reproduction* 2020: **26(2)**; 62-68.

Taranissi M, El-Toukhy T, Gorgy A, and Verlinsky Y. Influence of maternal age on the outcome of PGD for aneuploidy screening in patients with recurrent implantation failure. *Reprod Biomed Online* 2005: **10(5)**; 628-632.

Tehraninejad ES, Kashani NG, Hosseini A, and Tarafdari A. Autologous platelet-rich plasma infusion does not improve pregnancy outcomes in frozen embryo transfer cycles in women with history of repeated implantation failure without thin endometrium. *J Obstet Gynaecol Res* 2021: **47(1)**; 147-151.

Tehraninejad ES, Raisi E, Bakhtiyari Ghaleh F, Hossein Rashidi B, Aziminekoo E, Kalantari V, Haghollahi F, and Shariat M. The sequential embryo transfer compared to blastocyst embryo transfer in in vitro fertilization (IVF) cycle in patients with the three repeated consecutive IVF. A randomized controlled trial. *Gynecological endocrinology* 2019: **35(11)**; 955-959.

Tempest N, Batchelor E, Hill CJ, Al-Lamee H, Drury J, Drakeley AJ, and Hapangama DK. Anterior Gradient Protein 3 and S100 Calcium-Binding Protein P Levels in Different Endometrial Epithelial Compartments May Play an Important Role in Recurrent Pregnancy Failure. *Int J Mol Sci* 2021: **22(8)**:3835.

Tersoglio AE, Salatino DR, Reinchisi G, Gonzalez A, Tersoglio S, and Marlia C. Repeated implantation failure in oocyte donation. What to do to improve the endometrial receptivity? *JBRA Assist Reprod* 2015: **19(2)**; 44-52.

Tersoglio AE, Salatino DR, Tersoglio S, Castro M, and Gonzalez A. Normalization of endometrial histopathology and endometrial NK cells concentration predict successful pregnancy in repeated implantation failure. *JBRA Assist Reprod* 2021: **25(1)**; 59-70.

Tersoglio AE, Tersoglio S, Salatino DR, Castro M, Gonzalez A, Hinojosa M, and Castellano O. Regenerative therapy by endometrial mesenchymal stem cells in thin endometrium with repeated implantation failure. A novel strategy. *JBRA Assist Reprod* 2020: **24(2)**; 118-127.

Tesarik J, Galán-Lázaro M, Conde-López C, Chiara-Rapisarda AM, and Mendoza-Tesarik R. The Effect of GH Administration on Oocyte and Zygote Quality in Young Women With Repeated Implantation Failure After IVF. *Front Endocrinol (Lausanne)* 2020: **11**; 519572.

Tian J, Zhang Z, Mei J, Kong N, Yan Y, Shen X, Zhou J, Zhang Y, Kang N, Zhen X*, et al.* Dysregulation of endometrial stromal serotonin homeostasis leading to abnormal phosphatidylcholine metabolism impairs decidualization in patients with recurrent implantation failure. *Hum Reprod Open* 2024: **2024(3)**:hoae042.

Tian Y, Huang J, Wang CC, Lin H, Huang X, Zhao Y, Liu L, and Zhang S. The impact of endometrial scratch performed in mid-luteal phase on the endometrium whole genome transcriptomic profiles in following menstrual cycle. *Human fertility* 2023: **26(4)**; 733-741.

Tohma YA, Musabak U, Gunakan E, Akilli H, Onalan G, and Zeyneloglu HB. The Role of Analysis of NK Cell Subsets in Peripheral Blood and Uterine Lavage Samples in Evaluation of Patients with Recurrent Implantation Failure. *J Gynecol Obstet Hum Reprod* 2020: **49(9)**; 101793.

Tong J, Niu YC, Wan AR, and Zhang T. Next-Generation Sequencing (NGS)-Based Preimplantation Genetic Testing for Aneuploidy (PGT-A) of Trophectoderm Biopsy for Recurrent Implantation Failure (RIF) Patients: a Retrospective Study. *Reproductive Sciences* 2021: **28(7)**; 1923-1929.

Torky H, Ahmad A, Hussein A, El-Desouky ES, Aly R, Ragab M, and Abo-Louz A. Comparing sequential vs day 3 vs day 5 embryo transfers in cases with recurrent implantation failure: randomized controlled trial. *JBRA Assist Reprod* 2021: **25(2)**; 185-192.

Torky H, El-Desouky ES, El-Baz A, Aly R, El-Taher O, Shata A, Hussein A, Marie H, Deif O, Eldemery A*, et al.* Effect of Intra Uterine Granulocyte Colony Stimulating Factor vs. Human Chorionic Gonadotropin at Ovum Pick Up Day on Pregnancy Rate in IVF/ICSI Cases With Recurrent Implantation Failure. *JBRA Assist Reprod* 2021: **26(2)**:274.

Tran HP, Ly LT, Do VN, Hoang TT, Tran TT, Le HN, Nguyen PT, Nguyen NA, and Huynh TN. Assessing the Impact of Genratest on Women With Recurrent Implantation Failure: A Single-Center Study. *Cureus* 2024: **16(1)**; e52256.

Tsai HW, Wang PH, Hsu PT, Chen SN, Lin LT, Li CJ, and Tsui KH. Laser irradiation pretreatment improves endometrial preparation of frozen-thawed embryo transfer in recurrent implantation failure patients. *Gynecological endocrinology* 2020: **36(8)**; 734‐738.

Tuckerman E, Mariee N, Prakash A, Li TC, and Laird S. Uterine natural killer cells in peri-implantation endometrium from women with repeated implantation failure after IVF. *J Reprod Immunol* 2010: **87(1-2)**; 60-66.

Turgut A, Goruk NY, Tunc SY, Agaçayak E, Alabalik U, Yalinkaya A, and Gül T. Expression of extracellular matrix metalloproteinase inducer (EMMPRIN) in the endometrium of patients with repeated implantation failure after in vitro fertilization. *Eur Rev Med Pharmacol Sci* 2014: **18(2)**; 275-280.

Turienzo A, Lledó B, Ortiz JA, Morales R, Sanz J, Llácer J, and Bernabeu R. Prevalence of candidate single nucleotide polymorphisms on p53, IL-11, IL-10, VEGF and APOE in patients with repeated implantation failure (RIF) and pregnancy loss (RPL). *Hum Fertil (Camb)* 2020: **23(2)**; 117-122.

Turkyilmaz E, Guner H, Erdem M, Erdem A, Biri AA, Konac E, Alp E, Onen HI, and Menevse S. NLF2 gene expression in the endometrium of patients with implantation failure after IVF treatment. *Gene* 2012: **508(1)**; 140-143.

Udumudi A and Lava C. Genetic markers for inherited thrombophilia related pregnancy loss and implantation failure in Indian population - implications for diagnosis and clinical management. *J Matern Fetal Neonatal Med* 2022: **35(25)**; 9406-9414.

Urman B, Ata B, Yakin K, Alatas C, Aksoy S, Mercan R, and Balaban B. Luteal phase empirical low molecular weight heparin administration in patients with failed ICSI embryo transfer cycles: a randomized open-labeled pilot trial. *Human reproduction (Oxford, England)* 2009: **24(7)**; 1640‐1647.

Vagnini LD, Nascimento AM, Canas MDCT, Renzi A, Oliveira-Pelegrin GR, Petersen CG, Mauri AL, Oliveira JBA, Baruffi RLR, Cavagna M*, et al.* The relationship between vascular endothelial growth factor 1154G/A polymorphism and recurrent implantation failure. *Medical Principles and Practice* 2015: **24(6)**; 533-537.

Valojerdi MR, Eftekhari-Yazdi P, Karimian L, and Ashtiani SK. Effect of laser zona pellucida opening on clinical outcome of assisted reproduction technology in patients with advanced female age, recurrent implantation failure, or frozen-thawed embryos. *Fertility and sterility* 2008: **90(1)**; 84‐91.

Valojerdi MR, Karimian L, Yazdi PE, Gilani MA, Madani T, and Baghestani AR. Efficacy of a human embryo transfer medium: a prospective, randomized clinical trial study. *Journal of assisted reproduction and genetics* 2006: **23(5)**; 207‐212.

van den Heuvel MJ, Peralta CG, Hatta K, Han VK, and Clark DA. Decline in number of elevated blood CD3(+) CD56(+) NKT cells in response to intravenous immunoglobulin treatment correlates with successful pregnancy. *Am J Reprod Immunol* 2007: **58(5)**; 447-459.

Vaquero E, Lazzarin N, Caserta D, Valensise H, Baldi M, Moscarini M, and Arduini D. Diagnostic evaluation of women experiencing repeated in vitro fertilization failure. *Eur J Obstet Gynecol Reprod Biol* 2006: **125(1)**; 79-84.

Varla-Leftherioti M, Keramitsoglou T, Parapanissiou E, Kurpisz M, Kontopoulou-Antonopoulou V, Tsekoura C, Kamieniczna M, Novokowska B, Paparistidis N, Vrani V*, et al.* HLA-DQA1*0505 sharing and killer immunoglobulin-like receptors in sub fertile couples: report from the 15th International Histocompatibility Workshop. *Tissue Antigens* 2010: **75(6)**; 668-672.

Vialard F, Hammoud I, Molina-Gomes D, Wainer R, Bergere M, Albert M, Bailly M, De Mazancourt P, and Selva J. Gamete cytogenetic study in couples with implantation failure: Aneuploidy rate is increased in both couple members. *Journal of assisted reproduction and genetics* 2008: **25(11-12)**; 539-545.

Vialard F, Lombroso R, Bergere M, Gomes DM, Hammond I, Bailly M, and Selva J. Oocyte aneuploidy mechanisms are different in two situations of increased chromosomal risk: older patients and patients with recurrent implantation failure after in vitro fertilization. *Fertility and sterility* 2007: **87(6)**; 1333-1339.

Vitale SG, Palumbo M, Conde-López C, Mendoza N, Mendoza-Tesarik R, and Tesarik J. Effect of growth hormone administration on ICSI outcomes in patients with polycystic ovary syndrome and recurrent implantation failure: A retrospective cross-over study. *Int J Gynaecol Obstet* 2021: **153(2)**; 357-358.

Vitale SG, Palumbo M, Rapisarda AMC, Carugno J, Conde-López C, Mendoza N, Mendoza-Tesarik R, and Tesarik J. Use of pentoxifylline during ovarian stimulation to improve oocyte and embryo quality: A retrospective study. *Journal of Gynecology Obstetrics and Human Reproduction* 2022: **51(6)**:102398.

Volovsky M, Healey M, MacLachlan V, and Vollenhoven BJ. Should intrauterine human chorionic gonadotropin infusions ever be used prior to embryo transfer? *Journal of assisted reproduction and genetics* 2018: **35(2)**; 273‐278.

Vomstein K, Reider S, Böttcher B, Watschinger C, Kyvelidou C, Tilg H, Moschen AR, and Toth B. Uterine microbiota plasticity during the menstrual cycle: Differences between healthy controls and patients with recurrent miscarriage or implantation failure. *Journal of reproductive immunology* 2022: **151**:103634.

Vomstein K, Voss P, Molnar K, Ainsworth A, Daniel V, Strowitzki T, Toth B, and Kuon RJ. Two of a kind? Immunological and clinical risk factors differ between recurrent implantation failure and recurrent miscarriage. *J Reprod Immunol* 2020: **141**; 103166.

von Grothusen C, Frisendahl C, Modhukur V, Lalitkumar PG, Peters M, Faridani OR, Salumets A, Boggavarapu NR, and Gemzell-Danielsson K. Uterine fluid microRNAs are dysregulated in women with recurrent implantation failure. *Human Reproduction* 2022: **37(4)**; 734-746.

Voullaire L, Collins V, Callaghan T, McBain J, Williamson R, and Wilton L. High incidence of complex chromosome abnormality in cleavage embryos from patients with repeated implantation failure. *Fertil Steril* 2007: **87(5)**; 1053-1058.

Voullaire L, Wilton L, McBain J, Callaghan T, and Williamson R. Chromosome abnormalities identified by comparative genomic hybridization in embryos from women with repeated implantation failure. *Mol Hum Reprod* 2002: **8(11)**; 1035-1041.

Wan S, Sun Y, Zong J, Meng W, Yan J, Chen K, Wang S, Guo D, Xiao Z, Zhou Q*, et al.* METTL3-dependent m(6)A methylation facilitates uterine receptivity and female fertility via balancing estrogen and progesterone signaling. *Cell Death and Disease* 2023: **14(6)**:349.

Wang B, Gao M, Yao Y, Shen H, Li H, Sun J, Wang L, and Zhang X. Enhancing endometrial receptivity: the roles of human chorionic gonadotropin in autophagy and apoptosis regulation in endometrial stromal cells. *Reproductive Biology and Endocrinology* 2024: **22(1)**:37.

Wang C, Feng Y, Zhou WJ, Cheng ZJ, Jiang MY, Zhou Y, and Fei XY. Screening and identification of endometrial proteins as novel potential biomarkers for repeated implantation failure. *PeerJ* 2021: **9**; e11009.

Wang F and Liu Y. Identification of key genes, regulatory factors, and drug target genes of recurrent implantation failure (RIF). *Gynecol Endocrinol* 2020: **36(5)**; 448-455.

Wang J and Duan S. Effect of human chorionic gonadotropin on endometrial receptivity of infertile patients with repeated implantation failure. *Journal of Xinxiang Medical University* 2023: **40(11)**; 1024-1031.

Wang L, Ma J, Diao L, Chen J, Cheng Y, Yang J, and Li L. Endometrial proteomic profile of patients with repeated implantation failure. *Frontiers in Endocrinology* 2023: **14**:1144393.

Wang M, Deng H, and Ye H. Intrauterine injection of human chorionic gonadotropin improves pregnancy outcome in patients with repeated implantation failure in frozen-thawed embryo transfer. *Zhong nan da xue xue bao. Yi xue ban [Journal of Central South University. Medical sciences]* 2019: **44(11)**; 1247‐1251.

Wang S, Liu L, Ma M, Wang H, Han Y, Guo X, Yeung WSB, Cheng Y, Zhang H, Dong F*, et al.* Preimplantation genetic testing for aneuploidy helps to achieve a live birth with fewer transfer cycles for the blastocyst FET patients with unexplained recurrent implantation failure. *Archives of Gynecology and Obstetrics* 2023: **308(2)**; 599-610.

Wang WJ, Zhang H, Chen ZQ, Zhang W, Liu XM, Fang JY, Liu FJ, and Kwak-Kim J. Endometrial TGF-β, IL-10, IL-17 and autophagy are dysregulated in women with recurrent implantation failure with chronic endometritis. *Reprod Biol Endocrinol* 2019: **17(1)**; 2.

Weissman A, Eldar I, Ravhon A, Biran G, Farhi J, Nahum H, Golan A, and Levran D. Timing intra-Fallopian transfer procedures. *Reprod Biomed Online* 2007: **15(4)**; 445-450.

Weissman A, Horowitz E, Ravhon A, Nahum H, Golan A, and Levran D. Zygote intrafallopian transfer among patients with repeated implantation failure. *Int J Gynaecol Obstet* 2013: **120(1)**; 70-73.

Wilton L, Voullaire L, Sargeant P, Williamson R, and McBain J. Preimplantation aneuploidy screening using comparative genomic hybridization or fluorescence in situ hybridization of embryos from patients with recurrent implantation failure. *Fertil Steril* 2003: **80(4)**; 860-868.

Woon EV, Nikolaou D, MacLaran K, Norman-Taylor J, Bhagwat P, Cuff AO, Johnson MR, and Male V. Uterine NK cells underexpress KIR2DL1/S1 and LILRB1 in reproductive failure. *Frontiers in Immunology* 2023: **13**:1108163.

Wu F, Chen X, Liu Y, Liang B, Xu H, Li TC, and Wang CC. Decreased MUC1 in endometrium is an independent receptivity marker in recurrent implantation failure during implantation window. *Reprod Biol Endocrinol* 2018: **16(1)**; 60.

Wu F, Mao D, Liu Y, Chen X, Xu H, Li TC, and Wang CC. Localization of Mucin 1 in endometrial luminal epithelium and its expression in women with reproductive failure during implantation window. *J Mol Histol* 2019: **50(6)**; 563-572.

Wu J, Lin S, Huang P, Qiu L, Jiang Y, Zhang Y, Meng N, Meng M, Wang L, Deng W*, et al.* Maternal anxiety affects embryo implantation via impairing adrenergic receptor signaling in decidual cells. *Communications biology* 2022: **5(1)**; 840.

Xi HT, Qiu L, Yao YX, Luo LZ, Sui LC, Fu YH, Weng QY, Wang J, Zhao JZ, and Zhao YZ. Noninvasive Chromosome Screening for Evaluating the Clinical Outcomes of Patients With Recurrent Pregnancy Loss or Repeated Implantation Failure. *Frontiers in Endocrinology* 2022: **13**:896357; 9.

Xia L, Tian L, Zhang S, Huang J, and Wu Q. Hormonal Replacement Treatment for Frozen-Thawed Embryo Transfer With or Without GnRH Agonist Pretreatment: A Retrospective Cohort Study Stratified by Times of Embryo Implantation Failures. *Frontiers in Endocrinology* 2022: **13**:803471.

Xia QC, Gao SZ, Song JY, Du DQ, Li CJ, Zhou Y, Zhang XB, Dong ZB, Ma YN, and Ma YX. Effectiveness of herb-partitioned moxibustion on the navel for pregnancy outcomes in patients with recurrent implantation failure undergoing in vitro fertilization and embryo transfer: a study protocol for a randomized controlled trial. *Trials* 2022: **23(1)**:217; 8.

Xia T, Liu Q, Ye Q, Xing W, Wang D, Li J, and Zang ZJ. Serum oxytocin profiles in patients with repeated implantation failure during IVF cycles. *Gynecol Endocrinol* 2018: **34(12)**; 1048-1052.

Xiu YL, Sun KX, Zhang Q, Xiao YH, Bai X, Chen Y, Zhao MS, and Yu YX. Outcome of Different Endometrial Preparation Protocols Prior to Frozen-Thawed Embryo Transfer on Pregnancy Outcomes in Women with Repeated Implantation Failure. *International Journal of Women's Health* 2023: **15**; 1835-1844.

Xu H, Zhou M, Cao Y, Zhang D, Han M, Gao X, Xu B, and Zhang A. Genome-wide analysis of long noncoding RNAs, microRNAs, and mRNAs forming a competing endogenous RNA network in repeated implantation failure. *Gene* 2019: **720**; 144056.

Xu Y, Du J, Zou Y, Lin X, Chen Y, Ma L, Jiang S, and Lin X. Precise hourly personalized embryo transfer significantly improves clinical outcomes in patients with repeated implantation failure. *Front Endocrinol (Lausanne)* 2024: **15**; 1408398.

Xu YY, Hao CF, Fang JY, Liu XQ, Xue PP, and Miao RC. Intrauterine Perfusion of Autologous Platelet-Rich Plasma Before Frozen-Thawed Embryo Transfer Improves the Clinical Pregnancy Rate of Women With Recurrent Implantation Failure. *Frontiers in Medicine* 2022: **9**:850002; 5.

Xue HM, Li YM, Chen YT, Li C, Zhang Y, Jin P, Li ML, and Lin Y. [Effect of staging treatment of Tongyuan acupuncture on pregnancy outcome in patients with recurrent implantation failure of thin endometrium type]. *Zhongguo Zhen Jiu* 2021: **41(12)**; 1338-1342.

Xue PP, Zhou WB, Fan WQ, Jiang JY, Kong CC, Zhou W, Zhou JM, Huang XY, Yang HY, Han Q*, et al.* Increased METTL3-mediated m(6)A methylation inhibits embryo implantation by repressing HOXA10 expression in recurrent implantation failure. *Reproductive Biology and Endocrinology* 2021: **19(1)**:187; 11.

Yakin K, Ata B, Ercelen N, Balaban B, and Urman B. The effect of preimplantation genetic screening on the probability of live birth in young women with recurrent implantation failure; a nonrandomized parallel group trial. *Eur J Obstet Gynecol Reprod Biol* 2008: **140(2)**; 224-229.

Yakin K, Balaban B, Isiklar A, and Urman B. Oocyte dysmorphism is not associated with aneuploidy in the developing embryo. *Fertility and sterility* 2007: **88(4)**; 811-816.

Yamamoto S, Umeki M, Kodoma M, Hamano T, and Matsusita F. Beneficial effect of long zona dissection on frozen-thawed blastocysts at a young age. *Reproductive Medicine and Biology* 2007: **6(4)**; 211-218.

Yamazaki A, Kuroda T, Kawasaki N, Kato K, Shimojima Yamamoto K, Iwasa T, Kuwahara A, Taniguchi Y, Takeshita T, Kita Y*, et al.* Preimplantation genetic testing using comprehensive genomic copy number analysis is beneficial for balanced translocation carriers. *Journal of Human Genetics* 2024: **69(1)**; 41-45.

Yan Q, Zhao M, Hao F, Zhao R, Teng X, He B, Zhu C, Chen Z, and Li K. Effect of hyaluronic acid-enriched transfer medium on frozen-thawed embryo transfer outcomes in RIF patients: a single-centre retrospective study. *Frontiers in Endocrinology* 2023: **14**:1170727.

Yang J, Lu Y, Zhang Y, Zhou C, Liang Q, and Liang T. Acupuncture combined with gonadotropin-releasing hormone agonists improves endometrial receptivity and pregnancy outcome in patients with recurrent implantation failure of in vitro fertilization-embryo transfer. *J Assist Reprod Genet* 2024: **41(8)**; 2185-2192.

Yang KM, Ntrivalas E, Cho HJ, Kim NY, Beaman K, Gilman-Sachs A, and Kwak-Kim J. Women with multiple implantation failures and recurrent pregnancy losses have increased peripheral blood T cell activation. *Am J Reprod Immunol* 2010: **63(5)**; 370-378.

Yang R, Du X, Wang Y, Song X, Yang Y, and Qiao J. The hysteroscopy and histological diagnosis and treatment value of chronic endometritis in recurrent implantation failure patients. *Arch Gynecol Obstet* 2014: **289(6)**; 1363-1369.

Yang W, Sun Q, Zhou ZH, Gao Y, Shi F, Wu XY, Yang Y, Feng W, Wu Z, and Kang XM. Coagulation parameters predictive of repeated implantation failure in Chinese women A retrospective study. *Medicine* 2020: **99(48)**:e23320; 6.

Yang X, Huang R, Wang Y, and Liang X. Pituitary suppression before frozen embryo transfer is beneficial for patients suffering from idiopathic repeated implantation failure. *Journal of Huazhong University of Science and Technology* 2016: **36**; 127-131.

Yang Y, Chen X, Saravelos SH, Liu Y, Huang J, Zhang J, and Li TC. HOXA-10 and E-cadherin expression in the endometrium of women with recurrent implantation failure and recurrent miscarriage. *Fertil Steril* 2017: **107(1)**; 136-143.e132.

Yang ZY, Li Q, Yuan F, Wang MY, Zhang RZ, Chen YW, Fu WT, Yang QL, and Hu LL. Decreased NOTCH1 signaling activated autophagy in the mid-secretory endometrium of patients with recurrent implantation failure<SUP>†</SUP>. *Biology of Reproduction* 2023: **108(6)**; 974-987.

Yin B, Zeng Y, Wu TH, Yu SY, Xu J, Liu S, Diao LH, Zhao ZF, Liang DS, and Li YY. Functional properties of peripheral CD8+ T cells in patients with repeated implantation failure. *American journal of reproductive immunology* 2017: **78(2)**:e12704; 6.

Yoshii N, Hamatani T, Inagaki N, Hosaka T, Inoue O, Yamada M, Machiya R, Yoshimura Y, and Odawara Y. Successful implantation after reducing matrix metalloproteinase activity in the uterine cavity. *Reprod Biol Endocrinol* 2013: **11**; 37.

You XM, Zhang JX, Yang J, Lin QP, Lin J, Qiu JH, Gao HH, and Xu JB. [Clinical study on the effect of acupuncture on pregnancy outcome in patients with prethrombotic infertility after repeated implantation failure]. *Zhen Ci Yan Jiu* 2023: **48(9)**; 939-945.

Yu G, Xiang Y, Li Y, Chu X, Tong L, Dou Q, Lu N, and Tan L. Effects of human autologous mononuclear cells on expression of leukemia inhibitory factor and its receptor in co-culture model of embryo and endometrium in vitro. *Chinese Journal of Reproduction and Contraception* 2020: **40(2)**; 124-128.

Yu L, Wang L, Yan S, Chen S, Xu Q, Su D, and Wang X. Identification and validation of immune cells and hub genes alterations in recurrent implantation failure: A GEO data mining study. *Frontiers in Genetics* 2023: **13**:1094978.

Yu L, Ye J, Chen Q, and Hong Q. lncRNA TTTY14 participates in the progression of repeated implantation failure by regulating the miR-6088/SEMA5A axis. *Journal of assisted reproduction and genetics* 2024: **41(3)**; 727-737.

Yu N, Zhang B, Xu M, Wang S, Liu R, Wu J, Yang J, and Feng L. Intrauterine administration of autologous peripheral blood mononuclear cells (PBMCs) activated by HCG improves the implantation and pregnancy rates in patients with repeated implantation failure: a prospective randomized study. *American journal of reproductive immunology (New York, N.Y. : 1989)* 2016: **76(3)**; 212‐216.

Yu S, Kuang YP, and Qi C. Effects of Yupei Qisun Sequential Method of Chinese medicine on correlated indices of repeated implantation failure patients in the fresh cycle. *Zhongguo zhong xi yi jie he za zhi zhongguo zhongxiyi jiehe zazhi = chinese journal of integrated traditional and western medicine* 2012: **32(6)**; 763‐765.

Yu S, Lian R, Chen C, Chen X, Xu J, Zeng Y, and Li Y. Impact of body mass index on peripheral and uterine immune status in the window of implantation in patients with recurrent reproductive failure. *Human fertility* 2023: **26(5)**; 1322-1333.

Yu SL, Jeong DU, Noh EJ, Jeon HJ, Lee DC, Kang M, Kim TH, Lee SK, Han AR, Kang J*, et al.* Exosomal miR-205-5p Improves Endometrial Receptivity by Upregulating E-Cadherin Expression through ZEB1 Inhibition. *International Journal of Molecular Sciences* 2023: **24(20)**:15149.

Yu X, Gao C, Dai C, Yang F, and Deng X. Endometrial injury increases expression of hypoxia-inducible factor and angiogenesis in the endometrium of women with recurrent implantation failure. *Reproductive biomedicine online* 2019: **38(5)**; 761‐767.

Yuan B, Luo SH, Mao JB, Luo BB, and Wang JL. Effects of intrauterine infusion of platelet-rich plasma on hormone levels and endometrial receptivity in patients with repeated embryo implantation failure. *American Journal of Translational Research* 2022: **14(8)**; 5651-5659.

Yurci A, Dokuzeylul Gungor N, and Gurbuz T. Spectroscopy analysis of endometrial metabolites is a powerful predictor of success of embryo transfer in women with implantation failure: a preliminary study. *Gynecol Endocrinol* 2021: **37(5)**; 415-421.

Zahiri Z, Sarrafzadeh Y, Leili EKN, and Sheibani A. Success Rate of Hysteroscopy and Endometrial Scratching in Repeated Implantation Failure: A Randomized Controlled Clinical Trial. *Galen Medical Journal* 2021: **10**:e1399; 10.

Zamaniyan M, Peyvandi S, Gorji HH, Moradi S, Jamal J, Aghmashhadi FYP, and Mohammadi MH. Effect of platelet-rich plasma on pregnancy outcomes in infertile women with recurrent implantation failure: a randomized controlled trial. *Gynecological endocrinology* 2021: **37(2)**; 141-145.

Zargar M, Ghafourian M, Behrahi F, Nikbakht R, and Salehi AM. Association of recurrent implantation failure and recurrent pregnancy loss with peripheral blood natural killer cells and interferon-gamma level. *Obstet Gynecol Sci* 2024: **67(1)**; 112-119.

Zargar M, Ghafourian M, Nikbakht R, Hosseini VM, and Choghakabodi PM. Evaluating Chronic Endometritis in Women with Recurrent Implantation Failure and Recurrent Pregnancy Loss by Hysteroscopy and Immunohistochemistry. *Journal of Minimally Invasive Gynecology* 2020: **27(1)**; 116-121.

Zeng H, Fan X, and Liu N. Expression of H19 imprinted gene in patients with repeated implantation failure during the window of implantation. *Arch Gynecol Obstet* 2017: **296(4)**; 835-839.

Zeng H, Fu Y, Shen L, and Quan S. MicroRNA signatures in plasma and plasma exosome during window of implantation for implantation failure following in-vitro fertilization and embryo transfer. *Reproductive Biology and Endocrinology* 2021: **19(1)**:180.

Zeng H, Fu Y, Shen L, and Quan S. Integrated Analysis of Multiple Microarrays Based on Raw Data Identified Novel Gene Signatures in Recurrent Implantation Failure. *Front Endocrinol (Lausanne)* 2022: **13**; 785462.

Zeyneloglu HB, Tohma YA, Onalan G, and Moran U. Granulocyte colony-stimulating factor for intracytoplasmic sperm injection patients with repeated implantation failure: which route is best?dagger. *Journal of Obstetrics and Gynaecology* 2020: **40(4)**; 526-530.

Zhai J, Li S, Hu J, Gao M, Sun Y, Chen ZJ, Giudice LC, and Du Y. In Silico, In Vitro, and In Vivo Analysis Identifies Endometrial Circadian Clock Genes in Recurrent Implantation Failure. *J Clin Endocrinol Metab* 2021: **106(7)**; 2077-2091.

Zhai J, Ma LN, Chang ZY, and Yu T. Increased expression of prokineticin 2 and its receptor in endometrium of recurrent implantation failure patients decreased the expression of MMP9 important for decidualization. *Reproductive Biology and Endocrinology* 2022: **20(1)**:76; 11.

Zhai XY, Shu MM, Guo YM, Yao S, Wang YR, Han SJ, Song CL, Chuai Y, Wang QH, Ma F*, et al.* Efficacy of low-dose hCG on FET cycle in patients with recurrent implantation failure. *Frontiers in Endocrinology* 2022: **13**:1053592; 11.

Zhang F, Wang Z, Lian R, Diao L, Li Y, Wu Y, Yin T, and Huang C. Intrauterine perfusion of dexamethasone improves pregnancy outcomes in recurrent reproductive failure patients with elevated uterine natural killer cells. A retrospective cohort study. *American journal of reproductive immunology* 2023: **90(6)**:e13796.

Zhang H, Huang C, Chen X, Li L, Liu S, Li Y, Zhang Y, Zeng Y, and Hu L. The number and cytotoxicity and the expression of cytotoxicity-related molecules in peripheral natural killer (NK) cells do not predict the repeated implantation failure (RIF) for the in vitro fertilization patients. *Genes and Diseases* 2020: **7(2)**; 283-289.

Zhang H, Kong L, Cao Z, Zhu Y, Jiang Y, Wang X, Jiang R, Liu Y, Zhou J, Kang Y*, et al.* EHD1 impaired decidualization of endometrial stromal cells in recurrent implantation failure: role of SENP1 in modulating progesterone receptor signalling. *Biology of Reproduction* 2024: **110(3)**; 536-547.

Zhang H, Zhang C, and Zhang S. Single-Cell RNA Transcriptome of the Human Endometrium Reveals Epithelial Characterizations Associated with Recurrent Implantation Failure. *Advanced biology* 2023: **8(1)**:e2300110.

Zhang H, Zou H, Zhang C, and Zhang S. Chronic endometritis and the endometrial microbiota: implications for reproductive success in patients with recurrent implantation failure. *Annals of Clinical Microbiology and Antimicrobials* 2024: **23(1)**:49.

Zhang M, Ge T, Zhang Y, and La X. Identification of MARK2, CCDC71, GATA2, and KLRC3 as candidate diagnostic genes and potential therapeutic targets for repeated implantation failure with antiphospholipid syndrome by integrated bioinformatics analysis and machine learning. *Frontiers in Immunology* 2023: **14**:1126103.

Zhang Q, Ni T, Dang Y, Ding L, Jiang J, Li J, Xia M, Yu N, Ma J, Yan J*, et al.* MiR-148a-3p may contribute to flawed decidualization in recurrent implantation failure by modulating HOXC8. *J Assist Reprod Genet* 2020: **37(10)**; 2535-2544.

Zhang Q, Zhang B, Yan J, Zhang C, Tang R, Sheng Y, Wang N, and Chen ZJ. Intracavitary physiotherapy is not inferior to endometrial scratching in patients with recurrent implantation failure. *Arch Gynecol Obstet* 2015: **291(1)**; 173-177.

Zhang R, Xu A, Wang Q, Zhang L, Zhu L, Zhao S, and Xiong W. Fertiloscopy improves in vitro fertilization for women with repeated implantation failure. *J Gynecol Obstet Hum Reprod* 2017: **46(10)**; 743-746.

Zhang T, Huang C, Du Y, Lian R, Mo M, Zeng Y, and Mor G. Successful treatment with intrauterine delivery of dexamethasone for repeated implantation failure. *Am J Reprod Immunol* 2017: **78(6)**:e12766.

Zhang WB, Li H, Lu X, Chen JL, Li L, Chen JC, Wu H, and Sun XX. The clinical efficiency of transcriptome-based endometrial receptivity assessment (Tb-ERA) in Chinese patients with recurrent implantation failure (RIF): A study protocol for a prospective randomized controlled trial. *Contemp Clin Trials Commun* 2022: **28**; 100928.

Zhang WB, Li J, Li Q, Lu X, Chen JL, Li L, Chen H, Fu W, Chen JC, Lu BJ*, et al.* Endometrial transcriptome profiling of patients with recurrent implantation failure during hormone replacement therapy cycles. *Frontiers in Endocrinology* 2024: **14**:1292723; 17.

Zhang X, Gao Y, Liu W, Liu J, Wu L, Xiong S, Zhu J, Han W, Wang J, Hao X*, et al.* Frozen blastocyst embryo transfer vs. frozen cleavage-stage embryo transfer in couples with recurrent implantation failure: a cohort study. *Human fertility* 2021: **24(4)**; 284-289.

Zhang X, Guo F, Wang Q, Bai W, and Zhao A. Low-dose aspirin treatment improves endometrial receptivity in the midluteal phase in unexplained recurrent implantation failure. *Int J Gynaecol Obstet* 2021: **156(2)**; 225-230.

Zhang XL, Fu YL, Kang Y, Qi C, Zhang QH, and Kuang YP. Clinical observations of sequential therapy with Chinese medicine and hysteroscopic mechanical stimulation of the endometrium in infertile patients with repeated implantation failure undergoing frozen-thawed embryo transfer. *Chinese journal of integrative medicine* 2015: **21(4)**; 249‐253.

Zhang XX and Wu XH. Decreased CD56+CD16-CD94+uNK cells in the mid-luteal phase in women with recurrent implantation failure are associated with IL-15 deficiency. *American journal of reproductive immunology* 2023: **90(6)**:e13794.

Zhang XX, Zhang ZC, Liu YS, Zhou L, Hu YQ, Zhang CH, Song WH, and Wu XH. Bioinformatic Analysis of the Significance of the KIR2DL4 Gene in Recurrent Implantation Failure. *Biochemical Genetics.* 2024.

Zhang Y, Gong X, Zhang M, Zhu Y, Wang P, Wang Z, Liu C, La X, and Ding J. Establishment and validation of a nomogram for subsequent first-cycle live births in patients diagnosed with recurrent implantation failure: a population-based analysis. *Frontiers in Endocrinology* 2024: **15**:1334599.

Zhang Y, Luo H, Shi R, Zhang Y, Ma J, and Zhang Y. Clinical research of sequential embryo transfer in frozen thawed cycles of patients with recurrent implantation failure. *Chinese Journal of Reproduction and Contraception* 2020: **40(11)**; 893-898.

Zhang Y, Xu H, Liu Y, Zheng S, Zhao W, Wu D, Lei L, and Chen G. Confirmation of chronic endometritis in repeated implantation failure and success outcome in IVF-ET after intrauterine delivery of the combined administration of antibiotic and dexamethasone. *Am J Reprod Immunol* 2019: **82(5)**; e13177.

Zhang YL, Yin BN, Li SC, Cui YY, and Liu JR. Friend leukemia integration 1 overexpression decreases endometrial receptivity and induces embryo implantation failure by promoting PART1 transcription in the endometrial epithelial cells. *PeerJ* 2023: **11**:e16105.

Zhao F, Guo Y, Shi Z, Wu M, Lv Y, and Song W. hsa_circ_001946 elevates HOXA10 expression and promotes the development of endometrial receptivity via sponging miR-135b. *Diagn Pathol* 2021: **16(1)**; 44.

Zhao F, Lv YZ, Wang J, Jiang Y, and Tian KK. Effect of active immunotherapy with lymphocytes on repeated implantation failure. *Chinese Journal of Tissue Engineering Research* 2014: **18(51)**; 8281-8285.

Zhao FY, Chen T, Zhao XH, Wang Q, Lan YL, Liang Y, Li Y, Wang SY, Yang Y, and Yang XK. LINC02190 inhibits the embryo-endometrial attachment by decreasing ITGAD expression. *Reproduction* 2022: **163(2)**; 107-118.

Zhao H, Chen L, Shan Y, Chen G, Chu Y, Dai H, Liu X, and Bao H. Hsa_circ_0038383-mediated competitive endogenous RNA network in recurrent implantation failure. *Aging (Albany NY)* 2021: **13(4)**; 6076-6090.

Zhao H, Lv N, Cong J, Chen G, Bao H, and Liu X. Upregulated RPA2 in endometrial tissues of repeated implantation failure patients impairs the endometrial decidualization. *Journal of assisted reproduction and genetics* 2023: **40(11)**; 2739-2750.

Zhao H, Yu M, Li Q, Chen G, Liu X, and Bao H. Excessive proliferating cell nuclear antigen attenuates endometrial adhesive capacity and decidualization in patients with recurrent implantation failure. *Human Reproduction* 2024: **39(7)**; 1533-1547.

Zhao HT, Hu SG, Qi J, Wang Y, Ding Y, Zhu QL, He YQ, Lu Y, Yao Y, Wang SY*, et al.* Increased expression of HOXA11-AS attenuates endometrial decidualization in recurrent implantation failure patients. *Molecular Therapy* 2022: **30(4)**; 1706-1720.

Zhao J, Hao J, Xu B, and Li Y. Recurrent implantation failure versus recurrent implantation success: a preliminary study at proteomic level. *Gynecological endocrinology* 2023: **39(1)**:2217261.

Zhao J, Luo L, Wang HF, Yan CF, Li DH, Wang YY, Zhang P, Wang LP, and Cai FM. Effect of uterine natural killer cells in the window endometrium of implantation on angiogenesis and relationship between uterine artery pulsation index and IVF treatment outcome. *Journal of Xi'an Jiaotong University (Medical Sciences)* 2014: **35(3)**; 385-389.

Zhao QH, Song YW, Chen J, Zhou X, Xie JL, Yao QP, Dong QY, Feng C, Zhou LM, Fu WP*, et al.* Embryo Transfer Strategies for Women with Recurrent Implantation Failure During the Frozen-thawed Embryo Transfer Cycles: Sequential Embryo Transfer or Double-blastocyst Transfer? *Current medical science* 2024: **44(1)**; 212-222.

Zhao XX, Zhao Y, Jiang YP, and Zhang Q. Deciphering the endometrial immune landscape of RIF during the window of implantation from cellular senescence by integrated bioinformatics analysis and machine learning. *Frontiers in Immunology* 2022: **13**:952708; 18.

Zhao Y, He D, Zeng H, Luo J, Yang S, Chen J, Abdullah RK, and Liu N. Expression and significance of miR-30d-5p and SOCS1 in patients with recurrent implantation failure during implantation window. *Reprod Biol Endocrinol* 2021: **19(1)**; 138.

Zheng J, Tang X, Han TL, Zhang C, and Zhang S. Metabolomics analysis of serum metabolites during endometrial transformation: association with recurrent implantation failure in hormonal replacement therapy-frozen embryo transfers cycles. *Journal of assisted reproduction and genetics* 2023: **40(10)**; 2473-2483.

Zhou H, Zheng XY, Xia WT, Ma QH, Li JM, Zeng Q, and Huang JZ. The Efficacy and Safety of the Zhuyun Formula and Auricular Acupressure for the Infertile Women with Recurrent Implantation Failure: A Randomized Controlled Trial. *Evidence-based Complementary and Alternative Medicine* 2022: **2022**:5274638; 15.

Zhou M, Gao Y, Wu S, Wang Y, and Yang J. USP22 is required for human endometrial stromal cell proliferation and decidualization by deubiquitinating FoxM1. *Cellular Signalling* 2024: **121**:111265.

Zhou M, Xu H, Zhang D, Si C, Zhou X, Zhao H, Liu Q, Xu B, and Zhang A. Decreased PIBF1/IL6/p-STAT3 during the mid-secretory phase inhibits human endometrial stromal cell proliferation and decidualization. *J Adv Res* 2021: **30**; 15-25.

Zhou Q, Yan G, Ding L, Liu J, Yu X, Kong S, Zhang M, Wang Z, Liu Y, Jiang Y*, et al.* EHD1 impairs decidualization by regulating the Wnt4/β-catenin signaling pathway in recurrent implantation failure. *EBioMedicine* 2019: **50**; 343-354.

Zhou T, Ni T, Li Y, Zhang Q, Yan J, and Chen ZJ. circFAM120A participates in repeated implantation failure by regulating decidualization via the miR-29/ABHD5 axis. *FASEB Journal* 2021: **35(9)**:e21872.

Zhou X, Duan J, Zhou W, Zhang A, and Chen Q. Upregulated α-actinin-1 impairs endometrial epithelial cell adhesion by downregulating NEBL in recurrent implantation failure. *iScience* 2024: **27(3)**; 109046.

Zhou X, Xu B, Zhang D, Jiang X, Chang HM, Leung PCK, Xia X, and Zhang A. Loss of CDYL Results in Suppression of CTNNB1 and Decreased Endometrial Receptivity. *Front Cell Dev Biol* 2020: **8**; 105.

Zhou XW, Cao Y, Zhou MJ, Han M, Liu MY, Hu YQ, Xu BF, and Zhang AJ. Decreased CD44v3 expression impairs endometrial stromal cell proliferation and decidualization in women with recurrent implantation failure. *Reproductive Biology and Endocrinology* 2022: **20(1)**:170; 11.

Zhu M, Yi S, Huang X, Meng J, Sun H, and Zhou J. Human chorionic gonadotropin improves endometrial receptivity by increasing the expression of homeobox A10. *Mol Hum Reprod* 2020: **26(6)**; 413-424.

Zhu Y, Wu T, Ye L, Li G, Zeng Y, and Zhang Y. Prevalent genotypes of methylenetetrahydrofolate reductase (MTHFR) in recurrent miscarriage and recurrent implantation failure. *J Assist Reprod Genet* 2018: **35(8)**; 1437-1442.

Zhu YC, Zhang ZQ, Ma ZH, Deng WF, Zhang YU, and Wu QF. Autophagy markers are dysregulated in the endometrial tissues of patients with unexplained repeated implantation failure. *Molecular Reproduction and Development* 2022: **89(12)**; 655-660.

Zou W, Liu D, Peng J, Tang Z, Li Y, Zhang J, and Liu Z. Sequential embryo transfer combined with intrauterine perfusion improved pregnancy outcomes in patients with recurrent implantation failure. *BMC Women's Health* 2024: **24(1)**:126.

Zou Y, Liu X, Chen P, Wang Y, Li W, and Huang R. The endometrial microbiota profile influenced pregnancy outcomes in patients with repeated implantation failure: a retrospective study. *Journal of reproductive immunology* 2023: **155**:103782.

Zou Y, Ming L, Ding J, Xiao Z, Li S, Yang J, Bao A, and Zhang Y. Low dosage of prednisone acetate combined with doxycycline in the treatment of chronic endometritis in patients with repeated implantation failure. *American journal of reproductive immunology* 2023: **89(6)**:e13713.
