# Supplementary material for: Variability and implications of recurrent implantation failure definitions used in the scientific literature: a systematic review
Source: Hum Reprod Open. 2025 Jun 18;2025(3):hoaf033. doi: 10.1093/hropen/hoaf033 (PMC12321291; doi:10.1093/hropen/hoaf033)
Supplement: hoaf033_Supplementary_Data [file hoaf033_supplementary_data.zip › Supplementary_Figure_S2-post adjudication clean.docx]

**Supplementary Figure S2.** Number of specifiers used for the definitions of recurrent implantation failure in the eligible studies
